# Interpretation of the efficacy-oriented components of decoction pieces in compounds based on the spectrum-effect relationship: Huangqin Qingfei decoction as an example

**DOI:** 10.1186/s13020-026-01341-z

**Published:** 2026-02-09

**Authors:** Yanping Liu, Zhe Jia, Yanan Song, Lin Yan, Ying Liu, Qing Zhang, Yun Wang, Cun Zhang

**Affiliations:** 1 State Key Laboratory for Quality Ensurance and Sustainable Use of Dao-Di Herbs, Institute of Chinese Materia Medica, China Academy of Chinese Medical Sciences, Beijing, 100700 China; 2 Department of Pharmacy and Health Management, Hebei Chemical and Pharmaceutical College, Shijiazhuang, 050026 China; 3 College of Pharmacy, Henan University of Chinese Medicine, Zhengzhou, 450008 China

**Keywords:** Acute lung injury, Efficacy-oriented components, Huangqin Qingfei decoction, Spectrum-effect relationship, Ultra-high performance liquid chromatography-orbitrap high-Resolution mass spectrometry

## Abstract

**Background:**

Acute lung injury (ALI) is a severe respiratory inflammatory disorder with high morbidity and mortality. Huangqin Qingfei Decoction (HQQFD), a classic traditional Chinese medicine formulation composed of *Scutellaria baicalensis* Georgi (Scutellariae Radix, SR) and *Gardenica jasminoides* Ellis (Gardeniae Fructus, GF), is clinically used for treating respiratory inflammation including ALI. However, the pharmacodynamic basis of HQQFD and the optimal combination of raw and processed forms of SR and GF remain unclear.

**Purpose of the research:**

This study aimed to evaluate the therapeutic differences among four clinical variants of HQQFD and identify key bioactive components using a lipopolysaccharide (LPS)-induced ALI rat model.

**Methods:**

ALI rats were induced by LPS and treated with four HQQFD combinations (different raw/processed forms of SR and GF) at low or high doses. Therapeutic effects were assessed by lung histopathological observation, injury scoring, determination of inflammatory cytokine levels, lung wet/dry (W/D) ratio, and bronchoalveolar lavage fluid (BALF) analysis. Ultra-high performance liquid chromatography-Orbitrap high-resolution mass spectrometry (UHPLC-Orbitrap HRMS) was used to characterize the chemical profiles of different HQQFD variants. Pearson correlation analysis, gray relational analysis (GRA), and Orthogonal partial least squares (OPLS) spectrum-effect relationship analyses were performed to identify efficacy-oriented components.

**Results:**

A total of 113 compounds were identified in HQQFD, among which 49 showed significant differences between raw and processed combinations. High-dose HQQFD groups exhibited superior therapeutic effects compared to low-dose groups, with the combination of wine Scutellariae Radix (WSR) and Gardeniae Fructus Praeparatus (GFP) being the most effective variant. This optimal combination significantly alleviated lung tissue damage, reduced inflammatory cytokine levels, improved lung W/D ratio, and attenuated BALF abnormalities in ALI rats. Spectrum-effect relationship analysis revealed 20 key bioactive components correlated with pharmacodynamic effects, in which 8 compounds include: geniposidic acid, genipin 1-gentiobioside, geniposide, baicalin, glychionide A, wogonoside, oroxylin A and crocetin was detected in lung tissues, these components are more closely related and may synergise more targets in the treatment of ALI.

**Conclusion:**

These findings demonstrate that HQQFD ameliorates LPS-induced ALI in rats by regulating the inflammatory response. The combination of WSR and GFP at high dose shows the best therapeutic effect, and the 20 identified compounds are potential pharmacodynamic substances of HQQFD. This study provides experimental evidence for the clinical optimization and quality control of HQQFD in the treatment of ALI.

**Supplementary Information:**

The online version contains supplementary material available at 10.1186/s13020-026-01341-z.

## Introduction

Acute lung injury (ALI) is a life-threatening respiratory disorder triggered by direct or indirect cardiogenic factors including bacterial infections, contusions, burns, aspiration, and hemorrhagic shock [1, 2]. Characterized by acute hypoxic respiratory failure, increased alveolar permeability, and severe alveolar edema, ALI can rapidly progress to acute respiratory distress syndrome (ARDS) without timely and effective intervention. Clinically, ARDS is associated with high morbidity, poor prognosis, and substantial medical costs in intensive care units (ICUs). A large-scale multicenter trial involving 459 ICUs across 50 countries reported a 43% 90-day hospital mortality rate among patients with moderate-to-severe ARDS [3], highlighting an urgent need for the development of safe and effective therapeutic strategies.

Traditional Chinese medicine (TCM) has a long-standing clinical history of over thousands of years in China and other East Asian countries for treating respiratory diseases [4, 5]. Huangqin Qingfei Decoction (HQQFD), formulation documented in the ancient medical text “Wei Sheng Bao Jian” (*卫生宝鉴*), edited by Luo Tianyi during the Yuan Dynasty (1220–1290), consists of two medicinal herbs: *Scutellaria baicalensis* Georgi (Scutellariae Radix, SR) and *Gardenica jasminoides* Ellis (Gardeniae Fructus, GF). Clinically, HQQFD is primarily used for treating the TCM syndrome of “lung heat toxin accumulation,” whose traditional indications—including fever, asthma, and cough with yellow or purulent sputum—are highly consistent with the clinical manifestations of respiratory inflammatory diseases such as pneumonia and acute bronchitis in modern medicine. From the perspective of TCM pathogenesis, the core pathological changes of ALI (e.g., acute inflammatory infiltration, alveolar edema, and tissue damage) are closely aligned with the “intense lung heat and phlegm-heat binding” syndrome that HQQFD targets. SR, bitter in taste and cold in nature, excels at clearing lung heat in the upper and middle jiao; GF, also bitter and cold with a descending property, can purge fire toxicity from the triple energizers. These two herbs exert synergistic effects to clear heat and purge fire, detoxify and resolve phlegm, and cool blood and reduce swelling, directly addressing the core pathogenesis of ALI.

Modern pharmacological studies have provided evidence for the protective effects of HQQFD against lipopolysaccharide (LPS)-induced ALI in rats [6]. Key bioactive components of its constituent herbs, such as baicalin from SR and geniposide from GF, have been shown to alleviate ALI through multiple mechanisms, including anti-inflammation, antioxidation, and inhibition of inflammatory cytokine release [7–11]. Notably, TCM processing is a crucial factor modulating the therapeutic effects of medicinal herbs. Due to regional differences in clinical practice across China, HQQFD exhibits variations in the selection of processed decoction pieces: SR is commonly used in two forms—raw Scutellariae Radix (RSR) and wine-processed Scutellariae Radix (WSR)—while GF is available as raw Gardeniae Fructus (RGF) and processed Gardeniae Fructus (GFP). According to TCM compatibility theory, the wine processing of SR enhances its efficacy in clearing and cooling lung fire, and previous studies have confirmed that WSR is more effective than RSR in treating ALI [7]. GF possesses a stronger bitter and cold nature, aiding in clearing fire from the triple energizers and more easily entering the “blood phase” after processing. Together, these herbs target both the “chi and blood phases” to treat symptoms such as fever, coughing, yellow phlegm, and expulsion of pus and blood due to pneumonia. This exemplifies the synergistic approach in clinical practice. However, few studies have compared the effectiveness of GF and GFP in this context.

TCM formulas typically involve the combination of multiple herbs to enhance therapeutic efficacy and minimize adverse effects [12]. Therefore, identifying the bioactive components of TCM within the context of formula compatibility is crucial for improving clinical outcomes [13]. However, existing research on HQQFD has a significant limitation: it predominantly focuses on raw medicinal materials, neglecting the fundamental TCM principle of “processing for medicinal use.” In recent years, there has been a substantial expansion of research investigating changes in chemical components and pharmacological mechanisms during TCM processing. Additionally, TCM exerts diverse pharmacological effects with distinct therapeutic focuses depending on the specific formula context. Based on these observations, we propose the concept of “efficacy-oriented components—bioactive constituents that are closely associated with the therapeutic effects of a formula. The identification of such components based on the specific efficacy of TCM formulas holds greater clinical significance and practical value.

Chromatographic fingerprinting, prized for its comprehensive chemical profiling, has become the benchmark methodology for both process standardization and quality control of TCM preparations. Notably, UHPLC fingerprinting offers distinct advantages in operational simplicity, analytical efficiency, and discriminatory power for characterizing complex chemical matrices. To bridge chemical composition with bioactivity, we established a spectrum-effect relationship model integrating chemical fingerprints with corresponding pharmacological outcomes. This approach was optimized using advanced chemometrics: including correlation algorithms [canonical correlation analysis (CCA), gray correlation analysis (GCA)], regression modeling [partial least squares (PLS), orthogonal partial least squares (OPLS)], machine learning [support vector machine (SVM)]. Through correlative modeling, bioactive constituents—particularly those exhibiting significant formulation abundance—can be systematically identified based on efficacy-composition covariance patterns.

To address the aforementioned research gaps, this study aimed to develop a spectrum-effect relationship approach to identify the efficacy-oriented components of processed decoction pieces in HQQFD for ALI treatment. The research design followed a systematic three-step approach: First, we combined UHPLC-Orbitrap high-resolution mass spectrometry (HRMS) and UHPLC-photodiode array (PDA) detection to establish the characteristic fingerprints of HQQFD with different processing combinations, followed by multivariate statistical analysis to identify differential chemical components among these combinations. Second, the Analytic Hierarchy Process-Entropy Weight method (AHP-EWM) was employed to comprehensively evaluate and compare the therapeutic effects of different processing combinations in LPS-induced ALI rats. Third, Pearson correlation analysis, gray relational analysis (GRA), and OPLS regression were used to elucidate the relationship between differential chemical components and pharmacological efficacy. Finally, the results of these analyses were integrated to screen for potential efficacy-oriented components, which could serve as quality control markers for WSR and GFP. This study not only provides innovative insights into the TCM theory of processing and compatibility but also establishes a robust methodological framework for the discovery of bioactive components in TCM formulas.

## Materials and methods

### Materials and reagent

RSR was obtained from Beijing Bencaofangyuan Pharmacy Group, Ltd. (Batch No. Y10470-230214; Neimenggu, China). RGF was obtained from Jiangxi Xinlong Agricultural Development Co. Ltd. (Batch No. 20220821, Jiangxi, China). Both materials were authenticated by Prof. Cun Zhang of the Institute of Chinese Materia Medica, China Academy of Chinese Medical Sciences (Beijing, China). The WSR was prepared from the same batch as the aforementioned RSR, while the GFP was derived from the same batch as the abovementioned RGF. Briefly, the processing procedure for WSR was as follows: RSR was rinsed with yellow rice wine (15 kg yellow rice wine per 100 kg of RSR) for a specified duration, stir-fried at 190 °C for 21 min, and then cooled to room temperature. GFP was also processed from GF from the same batch using methods described in our previous study [14]. Both WSR and GFP were prepared by experienced senior herbalists at Beijing Bencaofangyuan Pharmaceutical Co., Ltd., with the processing parameters and determination of processing endpoints strictly adhering to the requirements specified in the Chinese Pharmacopoeia (2020 Edition, Volume I). Dexamethasone acetate tablets (DEX) were purchased from the Zhejiang Xianju Pharmaceutical Co. Ltd. (Zhejiang, China). Quantikine® ELISA kits for IL-1β, IL-6 and TNF-α were obtained from R&D Systems, Inc. (Minneapolis, MN, USA). Geniposide was acquired from Chengdu Alfa Biotechnology Co., Ltd. (Chengdu, China), whereas crocin II, wogonoside, baicalein, and dihydrobaicalin were obtained from Chengdu Chroma-Biotechnology Co., Ltd. (Chengdu, China). Crocin I and scutellarin were obtained from Chengdu Must Biotechnology Co. Ltd. Gardenoside, 6α-hydroxygeniposide, 6β-hydroxygeniposide, and genipin 1-gentiobioside were obtained from Chengdu Pufei De Biotech Co. Ltd. Wogonin was obtained from Chengdu Push Bio-Technology Co. Ltd. (Chengdu, China). Chrysin was obtained from Jiangxi Baicaoyuan Biotechnology Co., Ltd., and geniposidic acid was purchased from Chengdu Desite Bio-Technology Co., Ltd. Oroxylin A-7-glucuronide, chrysin 7-glucuronide, baicalin, and oroxylin A were obtained from RENI Pharmaceutical Technology Co. Ltd. (Chengdu, China). Additional compounds, including chrysin-6-*C*-α-*L*-arabinoside-8-*C*-β-*D*-glucoside, norwogonin, chrysin-6-*C*-β-*D*-glucoside-8-*C*-α-*L*-arabinoside, viscidulin III, and glychionide A were purchased from Wuhan ChemFaces Biotechnology Co., Ltd. (Wuhan, China).

LPS and formic acid were purchased from Sigma–Aldrich (St. Louis, MO, USA). Methanol and acetonitrile (HPLC grade) were obtained from EMD Millipore. Ltd. (Billerica, MA, USA), and ultrapure water was supplied by A.S. Watson & Company. Telazol® 50 (a 1:1 combination of Tiletamine Hydrochloride and Zolazepam Hydrochloride) was obtained from Virbac Corporation and prepared as a 50 mg/mL stock solution by fully dissolving the lyophilized powder with the supplied water for injection.

### Decoction preparation

Four formulations of HQQFD were designated as PW1, PW2, PW3, and PW4, corresponding to the combinations of RSR + RGF, RSR + GFP, WSR + RGF, and WSR + GFP, respectively. The aqueous extracts were prepared as follows. The proportion of herb pairs was maintained at a 2:1 ratio. The herbs were immersed and decocted in eight volumes of water for 0.5 h, then filtered. This extraction process was repeated three times, with the combined decoctions evaporated under reduced pressure to approximately 100 mL and subsequently freeze-dried at low temperature (≤ -60 °C) for 48 h to obtain brown powder. The yields of the four prescriptions were 49.02, 40.07, 50.67, and 47.55% (*w/w*, dried extract/crude herb), respectively. Meanwhile, the single-herb decoction of RSR, WSR, RGF and GFP were prepared by the preparation method for HQQFD decoction. The yields of the four single-herb were 58.91, 57.19, 33.69 and 31.44%, respectively, and they were stored in a desiccator.

### Sample solution and standards preparation

The preparation processes for HQQFD (PW1, PW2, PW3, PW4) and single-herb (RSR, WSR, RGF and GFP) were consistent with the decoction preparation described above, excluding the evaporation and freeze-drying steps. Prior to the analysis, the samples were diluted five times and filtered through a membrane filter (0.22 µm).

All the standard solutions were prepared in 50% methanol. The concentrations of geniposidic acid, 6α-hydroxygeniposide, gardenoside, 6β-hydroxygeniposide, genipin 1-gentiobioside, geniposide, scutellarin, chrysin-6-*C*-α-*L*-arabinoside-8-*C*-β-*D*-glucoside, chrysin-6-*C*-β-*D*-glucoside-8-*C*-α-*L*-arabinoside, viscidulin III, baicalin, dihydrobaicalin, glychionide A, chrysin 7-glucuronide, oroxylin A-7-glucuronide, wogonoside, norwogonin, baicalein, wogonin, chrysin, oroxylin A, Crocin I, Crocin II, and Crocetin were 11.3, 5.3, 21.6, 18.6, 9.8, 40.8, 6.6, 19.2, 9.1, 10.3, 130.8, 18.8, 19.6, 11.3, 9.4, 66.0, 10.0, 2.8, 10.0, 9.2, 5.3, 25.0, 1.4, and 2.7 µg/mL, respectively.

### Apparatus and analytical condition

#### UHPLC-DAD condition

A Nexera × 2 UHPLC system (Shimadzu Corporation, Shanghai, China) comprising a DGU-20A5R degasser, SIL-30 AC autosampler, SPD-M20A DAD, CTO-30 A oven, and an LC-30AD dual pump was employed. Chromatographic separation was performed on a Waters Acquity UPLC BEH C18 column (2.1 × 100 mm, 1.7 µm) at a column temperature of 45 °C. The mobile phase consisted of (A) water containing formic acid (0.15%, *v/v*) and (B) mixture of acetonitrile and methanol (4:1, *v/v*). The flow rate was set at 0.25 mL/min, and a gradient elution program was utilized: 0–6 min, 4% B; 6–10 min, 4%–15% B; 10–15 min, 15%–15% B; 15–20 min, 15%–20% B; 20–25 min, 20%–23% B; 25–35 min, 23% B; 35–45 min, 23%–45% B; 45–60 min, 45%–55% B. The detection wavelengths were set at 254 and 440 nm, and the injection volume was 1 μL.

### UHPLC-Orbitrap HRMS condition

UHPLC-Orbitrap HRMS analysis was conducted using a Vanquish Duo UHPLC system (Thermo Scientific, USA) coupled with a PDA detector and cooling autosampler. Mass detection was performed using an Orbitrap Exploris™ 240 MS (Thermo Scientific, USA) equipped with a heated ESI source. The UHPLC conditions were consistent with UHPLC-DAD condition. The parameters for MS detection in negative ion scan modes included: spray voltage static; negative ion at -3.2 kV; ion transfer tube and evaporator temperatures at 320 °C and 400 °C, respectively; auxiliary gas, sweep gas, and sheath gas pressures at 10, 0, and 40 arbs, respectively; full MS scan range *m/z* 100–1500 with a resolution of 120,000; auto dynamic exclusion mode; number of dependent scans: 20. For MS/MS, the isolation window was 1 m*/z*, and the samples were analyzed at normalized collision energies of 30, 50, and 70 with a resolution of 30,000.

### Precision, repeatability and stability of characteristic spectrum analysis

The characteristic spectrum methodology was validated to assess its precision, repeatability, and stability by using a quality control (QC) sample solution prepared by pooling equal amounts of PW1, PW2, PW3, and PW4. The precision was evaluated by performing six consecutive analyses under the same UHPLC conditions. The repeatability was determined by testing six replicates of a single sample under identical conditions. Stability was assessed by analyzing the samples at various time points within a single day (0, 2, 4, 8, 12, 24, 36, and 48 h) post-preparation. The relative standard deviations (RSD) of the characteristic peak areas were calculated.

### Chemical profile data preprocessing

The results were analyzed based on precise relative molecular masses and primary and secondary mass spectral fragmentation data obtained using Xcalibur software. These data were supplemented with fragmentation information matched to a self-constructed library, which sourced data from ChemSpider (https://www.chemspider.com/), MassBank (https://massbank.eu/), the Human Metabolome Database (https://hmdb.ca/), and relevant literature.

### Experiments of the pharmacodynamic effect

#### Animal grouping and administration

Healthy male Wistar rats (200 ± 20 g) were procured from the SPF Biotechnology Co. Ltd. (Beijing, China) (License No.: SCXK (Beijing) 2024–0001). The Ethics Committee for the Welfare of Laboratory Animals at the Institute of Chinese Materia Medica of the China Academy of Chinese Medical Sciences approved this study (No. 2023B174). The animals were maintained under good laboratory conditions at an ambient temperature of 24 ± 1 °C and relative humidity of 55 ± 10%. They were kept on a 12 h light/dark cycle and had free access to standard feed and water.

After three days of quarantine and acclimatization, the rats were randomly divided into eight groups (*n* = 6 per group): control group (Con), model group (LPS), positive control group (LPS_DEX), PW1 low-dose group (4.6 g/kg, LPS_LPW1), PW1 high-dose group (9.2 g/kg, LPS_PW1), PW2 group (9.2 g/kg, LPS_PW2), PW3 group (9.2 g/kg, LPS_PW3), and PW4 group (9.2 g/kg, LPS_PW4).

### Animal model and treatment

A rat model of ALI was established by intratracheal administration of LPS. The animals were anesthetized by intramuscular injection of Telazol® 50 solution (40 mg/kg body weight) and placed in the supine position. The trachea was surgically exposed through a cervical midline incision and LPS (5 mg/kg body weight) was slowly injected into the trachea of rat. After 12 h, the ALI model was established [15].

After model establishment, rats in the treatment groups received the corresponding interventions by gavage: PW1 at 4.6 g/kg or 9.2 g/kg, PW2 ~ PW4 at 9.2 g/kg, and DEX as the positive control (DEX, 1 mg/kg). To determine an appropriate therapeutic dose for HQQFD intervention, a preliminary dose–response experiment was conducted using PW1 at 4.6 g/kg and 9.2 g/kg. The high dose (9.2 g/kg) produced a more pronounced protective effect against ALI; therefore, subsequent comparative efficacy experiments among PW1 ~ PW4 were performed at a fixed equivalent dose of 9.2 g/kg. The drugs used in the PW1 ~ PW4 groups were the corresponding freeze-dried powders, which were dissolved in deionized water prior to intragastric administration (all doses calculated based on crude drug weight).

All animals were euthanized and sampled 6 h after drug administration.

### Lung wet/dry weight ratio

All rats were euthanized at 6 h after treatment, and the samples were collected for subsequent analysis. The right lungs were immediately weighed to obtain the wet weight, and then placed in an oven at 80 °C for 48 h to obtain the dry weight. The wet-to-dry weight ratio (W/D ratio) was calculated to assess lung edema.

### Pathological analysis

The upper lobe of the right lung was fixed in 4% paraformaldehyde, dehydrated, embedded in paraffin, and sectioned into 5 µm slices. The sections were stained with hematoxylin and eosin (H&E). Pathological changes were observed at 100 × magnification and photographed at 200 × using CaseViewer (3DHISTEC, Sysmex, Switzerland). Following the methods described in the literature [16], three pathological sections were randomly selected from each group, and five non-overlapping high-power fields (200 ×) were randomly chosen per section for evaluation. The sections were scored according to the grading criteria outlined in Table 1. The lung injury score was calculated using Eq. 1.

**Table 1:** Lung injury scoring system

Parameter	Score per field		
	0	1	2
A: Neutrophils in the alveolar space	None	1–5	> 5
B: Neutrophils in the interstitial space	None	1–5	> 5
C: Hyaline membranes	None	1	> 1
D: Proteinaceous debris filling the airspaces	None	1	> 1
E: Alveolar septal thickening	< 2 ×	2 × ~ 4 ×	> 4 ×

1 $$lung injury score=\frac{\left[\left(20\times A\right)+\left(14\times B\right)+\left(7\times C\right)+\left(7\times D\right)+\left(2\times E\right)\right]}{number of fields\times 100}$$

### BALF collection and analysis

At the conclusion of the experiment, the lungs were ligated, and the left lungs were lavaged twice with 2 mL autoclaved PBS. The fluid recovery ratio was approximately 80% (4 mL). The BALF was centrifuged at 1000 rpm for 10 min at 4 °C, and the supernatants were stored at -80 °C for protein concentration analysis using the Bicinchoninic Acid (BCA) method. The cell pellets were resuspended in PBS and the number of neutrophils was counted using a blood cell analyzer (XI-800; Sysmex; Japan).

### ELISA assay

Approximately 0.2 g of lung tissue was minced and homogenized with four volumes of PBS in a tissue homogenizer and then centrifuged at 10,000 rpm at 4 °C. Aliquots of the supernatant were obtained and stored at -80 °C until analysis. The concentrations of TNF-α, IL-6, and IL-1β in the lungs were measured using commercial ELISA kits.

### Statistical analysis

Data were imported into SPSS statistical software (version 29.0, SPSS Inc., Chicago, IL, USA) and GraphPad Prism 8.0 (version 8.0, GraphPad Software Inc., California, USA) for analysis. One-way analysis of variance (ANOVA) was used to evaluate differences among groups. When homogeneity of variance was satisfied, the least significant difference (LSD) test was applied for post hoc comparisons; otherwise, Dunnett’s T3 test was used. A value of *P* < 0.05 was considered statistically significant.

To better reflect the changes in pharmacodynamic indicators after administration, the efficacy data were pre-processed. Inhibition rates of TNF-α, IL-1β, and IL-6 were used to replace the absolute values of the original pharmacodynamic indicators [17]. The characteristic peaks of the different samples were normalized using SPSS 29.0. The inhibition rates of the pharmacodynamic indicators were calculated using Eq. 2:

2 $$Inhibition rate \left(\%\right)=\frac{model group-treatment group}{model group}\times 100$$

### Evaluation of pharmacodynamic effects by AHP-EWM

The AHP-EWM was employed to determine the weights of the evaluated factors. AHP relies on subjective judgments and may be influenced by the professional background and cognitive preferences of the participating experts, while EWM is prone to being affected by extreme values due to information redundancy or irregular distribution within the evaluation system. To address the potential subjectivity of AHP, five experts specializing in TCM pharmacology and Chinese materia medica were invited to provide professional guidance and conduct rigorous review during the AHP weighting process. Additionally, EWM was utilized for objective weighting to mitigate the inherent biases associated with AHP. The integration of the AHP-EWM comprehensive weighting method was implemented using the SPSSPRO software. (https://www.spsspro.com, Version 1.0.11).

### Spectrum–effect correlation analysis

#### Pearson analysis

The Pearson correlation technique, a parametric approach, is extensively employed to examine the linear associations between variables. Researchers imported chromatographic data and pharmacodynamic indices (inhibition rate) into SPSS for correlation analysis. In these calculations, the standardized peak area and pharmacodynamic indicators were utilized as dependent variables. The magnitude of the correlation coefficient reflects the strength of the relationship between the variables under investigation.

### Gray relational analysis

Gray Relational Analysis (GRA) is a valuable tool for measuring the degree of correlation between factors based on the similarity or dissimilarity of their development trends. We employed EXCEL 2016 to assess the correlation between pharmacodynamic indicators and characteristic peaks. The pharmacodynamic indicator was treated as the primary sequence, whereas the standardized peak area was treated as a subsequent sequence to calculate the degree of association between the ingredients and pharmacodynamic indicators. A higher degree of association signifies a stronger correlation between ingredients and pharmacodynamic indicators [18]. The relation coefficients (ε(ki)) and grey relational degree (GRD, ri) were calculated by Eq. 3 and Eq. 4.

3 $$\varepsilon \left( {k_{i} } \right) = \frac{{{\mathrm{min}} + \rho {\mathrm{max}}}}{{{\mathrm{oi}}\left( {\mathrm{k}} \right) + \rho {\mathrm{max}}}}$$

4 $$n=\frac{1}{N}\sum_{k=1}^{n}\varepsilon \left(ki\right)$$

where,

1. Δoi(k) represents the absolute difference between the parent sequence and subsequence.
2. Δmin denotes the minimum value of the absolute difference between the parent sequence and subsequence.
3. Δmax represents the maximum value of the absolute difference between the parent sequence and sequence.
4. ρ is the distinguishing coefficient, which is typically set as 0.5.

### Orthogonal partial least squares analysis

OPLS models were constructed using SIMCA-P 14.1 software. The independent variable (X) was represented by the standardized peak area, while the pharmacodynamic data served as the dependent variable (Y). To identify the primary active compounds, a Variable Importance in Projection (VIP) value threshold of ≥ 1 was applied. The VIP value serves as an indicator of correlation strength, with higher values denoting stronger correlations between variables.

## Results

### Optimization of chromatographic conditions

To optimize the separation of the chemical components in HQQFD using HPLC, we refined the chromatographic conditions. By analyzing the chromatogram of HQQFD, the common characteristic peaks were selected for subsequent chemometric analysis. During systematic optimization, we examined the type of mobile phase, column temperature, and flow rate. The detailed optimization process is shown in Fig. S1-6. The results indicated that the type of column and mobile phase significantly influenced the separation outcomes, optimal results were shown that Waters column has a satisfactory result, and the optimal mobile phases consist an organic phase with acetonitrile-methanol = 4:1 (*v*:*v*) and aqueous phase using 0.15% formic acid. While column temperature and flow rate affected peak appearance time, we selected a column temperature of 45 °C and a flow rate of 0.2 mL/min to balance separation efficiency and detection time.

### Identification of peaks by UHPLC—Orbitrap HRMS

**Fig. 1:**
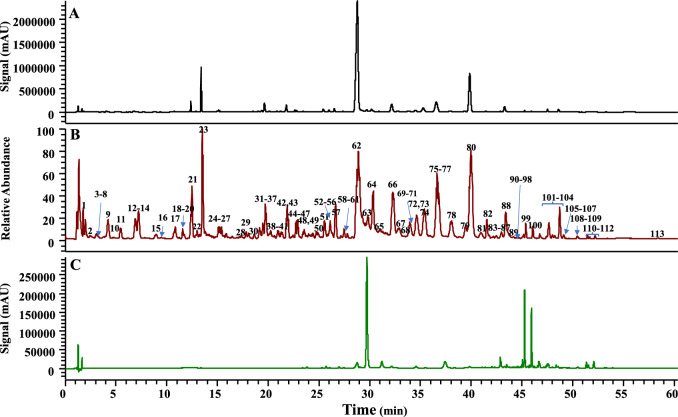
Chromatogram of UHPLC-PDA and total ion chromatogram (TIC) of HQQFD (PW1) under the same UHPLC conditions. **A** UHPLC-PDA spectrum under 254 nm. **B** TIC of HQQFD. **C** UHPLC-PDA spectrum under 440 nm

Data were collected using the Xcalibur software (Thermo Scientific, USA), which was used to fit the molecular formulas. The MS/MS spectra were compared with the MassBank and PubChem databases to identify compound structures and attributes. A total of 113 compounds were identified from the HQQFD, the summary results are presented in Table 2, and their total ion chromatogram (TIC) were shown in Fig. 1 and Fig. S7, with detailed structural identifications available in Tables S1–S4, they mainly consist of 32 free flavones, 6 flavone *C*-glycosides, 26 flavone *O*-glycosides, 1 flavone *O* &*C* glycoside, 24 monoterpenoids, 9 diterpenoids, 12 organic acids, 3 others, their cleavage patterns under mass spectrometry were mentioned in our previous literature [19], similarly, the cleavage rules of their representative components are presented in Fig. S8-Fig. S11.The findings revealed no differences in the types of components detected among the four compatibility groups. However, variations in the mass spectral responses of different components were observed.

**Table 2:** Summary results of identification of PW1, PW2, PW3 and PW4 by UHPLC-Orbitrap HRMS

No	t_R_ (min)	Formula	Identification	Structure type	Source	Characteristic Peak (Y/N)
1	1.37 ± 0	C_7_H_12_O_6_	Quinic acid	OA	GF	N
2	2.45 ± 0	C_16_H_22_O_11_	Deacetylasperulosidic acid	M	GF	N
3	3.13 ± 0	C_16_H_22_O_10_	Gardoside	M	GF	N
4	3.3 ± 0	C_16_H_24_O_11_	Shanzhiside isomer	M	GF	N
5	3.66 ± 0	C_9_H_8_O_4_	Caffeic acid	OA	SR	N
6	3.95 ± 0	C_17_H_26_O_11_	Shanzhiside methyl ester	M	GF	N
7	4.16 ± 0	C_7_H_6_O_4_	Protocatechuic acid	OA	SR	N
8	4.16 ± 0	C_16_H_24_O_11_	Shanzhiside	M	GF	N
9	4.33 ± 0	C_16_H_22_O_10_	Geniposidic acid	M	GF	Y
10	5.33 ± 0.02	C_16_H_18_O_9_	Chlorogenic acid	OA	SR	N
11	5.42 ± 0.05	C_17_H_24_O_11_	6α-hydroxygeniposide	M	GF	Y
12	6.9 ± 0.03	C_17_H_24_O_11_	Gardenoside	M	GF	Y
13	7.03 ± 0.06	C_16_H_24_O_10_	Mussaenosidic acid	M	GF	N
14	7.24 ± 0.03	C_16_H_26_O_8_	Jasminoside B	M	GF	N
15	8.95 ± 0.03	C_17_H_24_O_11_	6β-hydroxygeniposide	M	GF	Y
16	9.45 ± 0.09	C_16_H_26_O_8_	Picrocrocinic acid	M	GF	Y
17	10.71 ± 0.03	C_16_H_18_O_9_	Neochlorogenic acid	OA	GF	Y
18	11.68 ± 0.04	C_16_H_18_O_9_	Chlorogenic acid	OA	GF	Y
19	12.18 ± 0.05	C_7_H_6_O_4_	Protocatechuic acid-isomer	OA	SR	Y
20	12.38 ± 0.05	C_15_H_12_O_7_	3,6,7,2',6 '-pentahydroxyflavanones	F	SR	N
21	12.49 ± 0.03	C_23_H_34_O_15_	Genipin 1-gentiobioside	M	GF	Y
22	13.03 ± 0.04	C_17_H_22_O_10_	Sinapyglucoside	O	GF	Y
23	13.52 ± 0.03	C_17_H_24_O_10_	Geniposide	M	GF	Y
24	15.23 ± 0.02	C_15_H_10_O_7_	Quercetin	F	SR	N
25	15.54 ± 0.03	C_26_H_28_O_14_	Schaftoside	FC	SR	N
26	15.61 ± 0.04	C_16_H_26_O_7_	Picrocrocin	M	GF	Y
27	17.77 ± 0.03	C_27_H_30_O_16_	Rutin-isomer	FO	GF	Y
28	17.91 ± 0.05	C_21_H_20_O_12_	Carthamidin-7-*O*-glucuronide	FO	SR	Y
29	18.6 ± 0.03	C_27_H_30_O_16_	Rutin	FO	GF	N
30	19.32 ± 0.02	C_21_H_20_O_12_	Isoquercetin	FO	GF	Y
31	19.47 ± 0.08	C_21_H_18_O_12_	Scutellarin	FO	SR	Y
32	19.74 ± 0.03	C_26_H_28_O_13_	Chrysin 6-*C*-arabinoside 8-*C*-glucoside	FC	SR	Y
33	20.03 ± 0.03	C_23_H_24_O_13_	Viscidulin III-2′-*O*-glucoside	FO	SR	N
34	20.13 ± 0.04	C_16_H_26_O_8_	Jasminoside G	M	GF	N
35	20.54 ± 0.03	C_21_H_20_O_12_	Carthamidin 7-*O*-glucuronide isomer	FO	GF	N
36	21.7 ± 0.03	C_15_H_10_O_7_	3,5,7,2',6'-Pentahydroxy flavones	F	GF	N
37	21.75 ± 0.03	C_29_H_36_O_15_	Verbascoside	O	SR	N
38	21.82 ± 0.04	C_23_H_24_O_12_	Trihydroxy-dimethoxy-flavone glucoside	FO	SR	N
39	21.88 ± 0.08	C_26_H_28_O_13_	Chrysin 6-*C*-glucoside 8-*C*-arabinoside	FC	SR	Y
40	22.03 ± 0.01	C_25_H_24_O_12_	3,4-Dicaffeoyl quinic acid	OA	GF	N
41	22.27 ± 0.02	C_25_H_24_O_12_	3,5-Dicaffeoyl quinic acid	OA	GF	N
42	22.74 ± 0.03	C_26_H_28_O_13_	Chrysin 6-*C*-glucoside 8-*C*-arabinoside isomer	FC	SR	Y
43	22.87 ± 0.01	C_27_H_34_O_14_	Trihydroxydihydrochalcone-3'-*C*-glucoside-6'-*O*-glucoside or isomer	FOC	SR	Y
44	23.01 ± 0.04	C_16_H_12_O_7_	Tetrahydroxymethoxy-flavone	F	SR	N
45	23.46 ± 0.01	C_29_H_36_O_15_	Isoacteoside	O	SR	N
46	23.56 ± 0.03	C_21_H_20_O_9_	Chrysin 8-*C*-glucoside	FC	SR	N
47	23.58 ± 0.01	C_21_H_20_O_9_	Chrysin 6-*C*-glucoside	FC	SR	N
48	23.72 ± 0.05	C_21_H_20_O_10_	Apigenin 7-glucoside	FO	SR	N
49	24.31 ± 0.02	C_22_H_22_O_10_	Oroxylin A 7-*O*-*D*-glucuronide	FO	SR	N
50	24.44 ± 0.03	C_16_H_12_O_7_	Tetrahydroxymethoxy flavone	F	SR	N
51	25.55 ± 0.05	C_17_H_14_O_8_	Viscidulin III	F	SR	Y
52	25.97 ± 0.02	C_22_H_36_O_12_	Jasminoside Q	M	GF	N
53	26.04 ± 0.02	C_15_H_10_O_6_	Kaempferol or isomer	F	SR	N
54	26.1 ± 0.04	C_22_H_20_O_12_	Trihydroxy-methoxy-flavone-7-*O*-glucuronide	FO	SR	Y
55	26.33 ± 0.12	C_16_H_12_O_7_	Tetrahydroxymethoxy-flavone	F	SR	N
56	26.6 ± 0.03	C_32_H_40_O_17_	6''-*O*-[(*E*)-*p*-Coumaroyl] genipin gentiobioside or isomer	M	GF	Y
57	26.68 ± 0.05	C_31_H_32_O_16_	3,5-Di-*O*-Caffeoyl-4-*O*-(3-hydroxy-3-methyl) glutaroylquinic acid	OA	GF	N
58	27.2 ± 0.03	C_27_H_28_O_13_	3-*O*-Sinapoyl-5-*O*-caffeoylquinic acid	OA	GF	N
59	27.5 ± 0.03	C_34_H_44_O_19_	6''-*O*-[trans-Sinapoyl] genipin gentiobioside	M	GF	N
60	27.78 ± 0.04	C_33_H_42_O_18_	6''-*O*-[trans-Feruloyl] genipin gentiobioside	M	GF	N
61	27.99 ± 0	C_27_H_36_O_12_	6'-*O*-trans-Sinapoyljasminoside L	M	GF	N
62	28.81 ± 0.05	C_21_H_18_O_11_	Baicalin	FO	SR	Y
63	29.08 ± 0.04	C_27_H_28_O_13_	4-Sinapoyl-5-caffeoylquinic acid	OA	GF	N
64	29.83 ± 0.02	C_44_H_64_O_24_	Crocin I	D	GF	Y
65	30.32 ± 0.04	C_21_H_20_O_11_	Dihydrobaicalin	FO	SR	Y
66	30.58 ± 0.03	C_16_H_12_O_7_	Tetrahydroxymethoxy-flavone	F	SR	Y
67	32.31 ± 0.04	C_21_H_18_O_11_	Glychionide A	FO	SR	Y
68	32.97 ± 0.05	C_16_H_12_O_7_	Tetrahydroxymethoxy-flavone	F	SR	N
69	33.01 ± 0	C_22_H_22_O_10_	Oroxylin A 7-*O*-*D*-glucuronide isomer	FO	SR	Y
70	34.04 ± 0.04	C_21_H_18_O_11_	Norwogonin-8-*O*-glucuronide	FO	SR	Y
71	34.64 ± 0.03	C_22_H_20_O_12_	5,7,2'-Trihydroxy-6-methoxy-flavone-7-*O*-glucuronide	FO	SR	Y
72	35.01 ± 0.09	C_22_H_22_O_11_	5, 7-Dihydroxy-6-methoxy-flavanone-7-*O*-glucuronide	FO	SR	N
73	35.4 ± 0.04	C_21_H_18_O_10_	Chrysin-7-*O*-β-*D*-glucoronide	FO	SR	Y
74	36.76 ± 0.03	C_22_H_20_O_11_	Oroxylin A-7-*O*-glucuronide	FO	SR	Y
75	37.48 ± 0.06	C_38_H_54_O_19_	Crocin II	D	GF	Y
76	38.5 ± 0.04	C_18_H_16_O_8_	5,2',5'-Trihydroxy-6,7, 8-trimethoxy-flavonoids	F	SR	N
77	38.81 ± 0.04	C_21_H_20_O_10_	Dihydroxyflavanone-*O*-glucuronide	FO	SR	N
78	38.9 ± 0.07	C_21_H_20_O_10_	Trihydroxydihydroflavone-*O*-glucuronide	FO	SR	N
79	39.31 ± 0.09	C_15_H_10_O_6_	Kaempferol or isomer	F	SR	Y
80	39.44 ± 0.04	C_21_H_18_O_11_	Baicalein-6-*O*-glucuronide isobaric conformation	FO	SR	Y
81	39.72 ± 0.03	C_22_H_22_O_11_	Dihydroxy-methoxy-flavanone-*O*-glucuronide	FO	SR	N
82	39.98 ± 0.05	C_22_H_20_O_11_	Wogonoside	FO	SR	Y
83	40.95 ± 0.01	C_15_H_10_O_5_	Norwogonin	F	SR	Y
84	41.56 ± 0.03	C_23_H_22_O_12_	5, 7-Dihydroxy-6, 8-dimethoxy-flavone-7-*O*-glucuronide	FO	SR	N
85	41.97 ± 0.05	C_16_H_12_O_6_	Trihydroxymethoxy-flavone or isomer	F	SR	N
86	42.17 ± 0.04	C_27_H_36_O_11_	6'-*O*-Trans-sinapoyl jasminoside A	M	GF	N
87	42.2 ± 0.01	C_18_H_16_O_8_	5,2',5'-Trihydroxy-6,7, 8-trimethoxy-flavonoids	F	SR	N
88	42.47 ± 0.02	C_17_H_14_O_7_	Trihydroxy-dimethoxy-flavoneor isomer	F	SR	N
89	42.98 ± 0.05	C_16_H_12_O_6_	Trihydroxymethoxy-flavone	F	SR	Y
90	43.43 ± 0.03	C_15_H_10_O_5_	Baicalein	F	SR	Y
91	43.46 ± 0.03	C_28_H_34_O_14_	6'-*O*-Sinapoylgeniposide	M	GF	N
92	43.62 ± 0.01	C_17_H_14_O_7_	Trihydroxy-dimethoxy-flavone	F	SR	N
93	44.12 ± 0.04	C_16_H_12_O_6_	Trihydroxymethoxy-flavone or isomer	F	SR	N
94	44.71 ± 0.03	C_17_H_14_O_7_	Trihydroxy-dimethoxy-flavone or isomer	F	SR	N
95	45.37 ± 0.02	C_32_H_44_O_14_	all-trans-Crocetin di-β-*D*-glucosyl eater	D	GF	Y
96	46.1 ± 0.02	C_44_H_64_O_24_	Crocin I isomer	D	GF	Y
97	46.3 ± 0.03	C_17_H_14_O_7_	Trihydroxy-dimethoxy-flavone or isomer	F	SR	N
98	47.41 ± 0.02	C_18_H_16_O_7_	Skullcap flavone	F	SR	N
99	47.48 ± 0.01	C_18_H_16_O_8_	5,2',5'-Trihydroxy-6,7, 8-trimethoxy-flavonoids	F	SR	N
100	47.53 ± 0.02	C_27_H_36_O_11_	6'-*O*-trans-sinapoyl jasminoside A	M	GF	Y
101	47.64 ± 0.03	C_16_H_12_O_5_	Wogonin	F	SR	Y
102	47.99 ± 0.04	C_15_H_10_O_4_	Chrysin	F	SR	Y
103	48.27 ± 0.01	C_17_H_14_O_6_	Dihydroxy-dimethoxy-flavone	F	SR	Y
104	48.7 ± 0.02	C_19_H_18_O_8_	Skullcapflavone II	F	SR	Y
105	48.81 ± 0.02	C_16_H_12_O_5_	Oroxylin A	F	SR	Y
106	49.1 ± 0.02	C_30_H_18_O_10_	8,8''-Bibaicalein	F	SR	N
107	49.13 ± 0.01	C_17_H_14_O_6_	Dihydroxy-dimethoxy-flavone	F	SR	N
108	50.46 ± 0.02	C_18_H_16_O_7_	Tenaxin I	F	SR	N
109	50.6 ± 0.05	C_48_H_60_O_22_	Neocrocin B/C/D/E	D	GF	Y
110	51.47 ± 0	C_32_H_44_O_14_	Crocin-III or isomer 1	D	GF	Y
111	51.68 ± 0.01	C_32_H_44_O_14_	Crocin-III or isomer 2	D	GF	Y
112	52.2 ± 0	C_32_H_44_O_14_	Crocin-III or isomer 3	D	GF	Y
113	56.88 ± 0.22	C_20_H_24_O_4_	Crocetin	D	GF	Y

“Y” indicates a characteristic peak in the UHPLC chromatogram, while “N” indicates the absence of a characteristic peak. “SR” represents Scutellariae Radix; “GF”represents Gardeniae Fructus, *F* free flavones; *FO* flavone *O*-glycosides, *FC* flavone *C*-glycosides, *FO&C* flavone *O* &*C* glycoside, *M* monoterpenoids, *D* diterpenoids, *OA* Organic acids, *O* others

### Results of characteristic spectrum analysis

#### Characteristic peak identification and methodological validation

**Fig. 2:**
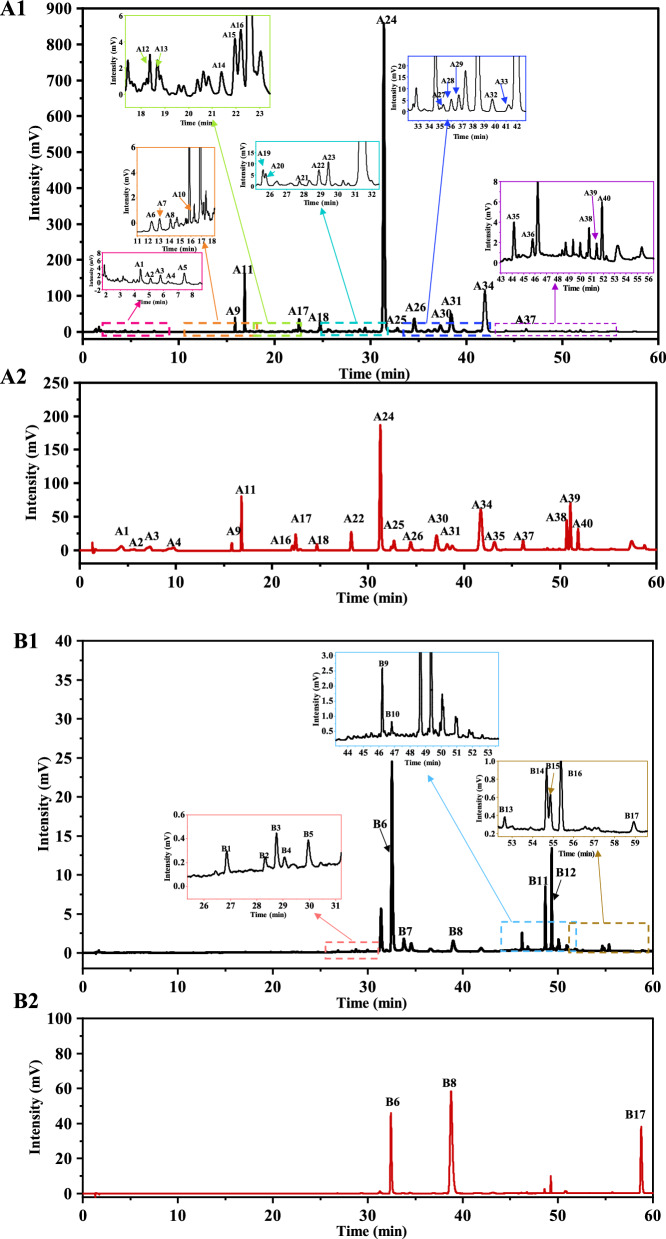

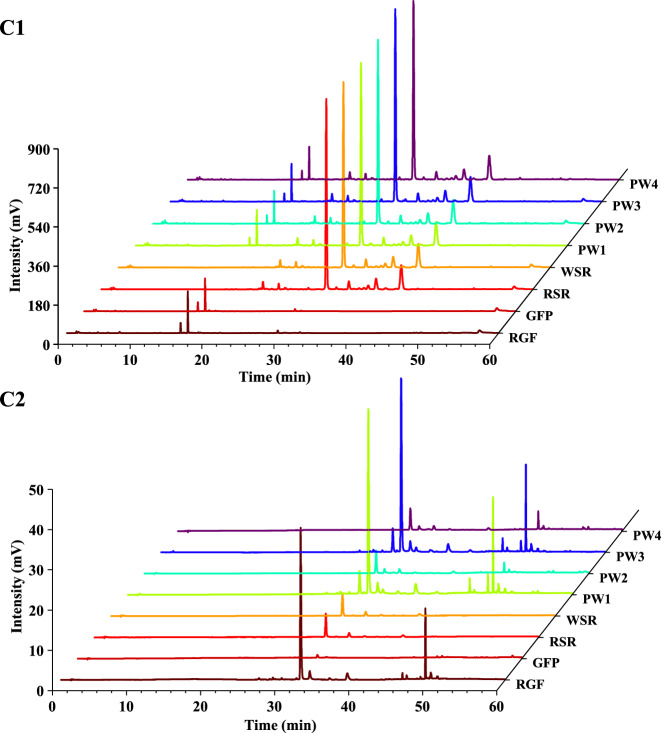
The characteristic spectrum of QC, standards, PW1~PW4, and four individual herbal medicines at 254 nm and 440 nm. **A1-2** Characteristic spectrum of QC and standards under 254 nm. **B1-2** Characteristic spectrum of QC and standards under 440 nm. **C1-2** Characteristic spectrum of PW1~PW4 and four individual herbal medicines under 254 nm and 440 nm

57 main peaks were identified based on the peak area, accounting for > 95% of the total peak area. Of these, 24 peaks were verified with standards, while the others were identified using UHPLC-Orbitrap HRMS. Method validation data are presented in Table S5, showing the RSD for precision, repeatability, and sample stability. The precision and repeatability results were both < 5.0%, confirming the suitability of the chromatographic conditions. In the stability test, the RSD of the samples was < 5%, indicating that the samples remained stable for 48 h. The characteristic spectra and peak areas of PW1–PW4 are shown in Fig. 2 and Table 3.

**Table 3:** Summary results of areas of the characteristic peaks

Peak No	RT (min)	PW1	PW2	PW3	PW4	Corresponding peak numbers in LC–MS	Identification	Source
A1*	4.962	2998 ± 163	17,988 ± 780	2752 ± 479	18,926 ± 875	9	Geniposidic acid	RGF/GFP
A2*	7.254	15,782 ± 1641	18,086 ± 6862	13,959 ± 2404	10,340 ± 823	11	6α-hydroxygeniposide	RGF/GFP
A3*	8.967	24,376 ± 1424	27,308 ± 1898	27,651 ± 1105	22,305 ± 7318	12	Gardenoside	RGF/GFP
A4*	10.061	20,505 ± 879	15,073 ± 846	20,870 ± 2052	11,790 ± 6655	15	6β-hydroxygeniposide	RGF/GFP
A5	11.823	49,745 ± 6098	15,921 ± 4484	56,507 ± 3724	14,876 ± 4795	16	Picrocrocinic acid	RGF/GFP
A6	13.955	10,809 ± 903	14,132 ± 1041	10,797 ± 503	13,190 ± 2380	17	Neochlorogenic acid	RGF/GFP
A7	14.597	18,657 ± 1329	20,724 ± 1238	18,376 ± 1923	17,596 ± 6766	18	Chlorogenic acid	RGF/GFP
A8	14.894	24,057 ± 325	15,983 ± 301	25,893 ± 1203	13,543 ± 3082	19	Protocatechuic acid	RGF/GFP
A9*	16.241	143,447 ± 4417	130,510 ± 8952	123,082 ± 5242	139,748 ± 12,734	21	Genipin 1-gentiobioside	RGF/GFP
A10	16.765	16,423 ± 2684	10,798 ± 452	20,768 ± 2332	9437 ± 2839	22	sinapyglucoside	RGF/GFP
A11*	17.226	695,037 ± 21,423	721,878 ± 83,827	733,526 ± 28,076	765,815 ± 23,316	23	Geniposide	RGF/GFP
A12	18.698	29,717 ± 1712	28,002 ± 5245	31,242 ± 2196	19,826 ± 3424	26	Picrocrocin	RGF/GFP
A13	19.001	36,244 ± 2202	37,164 ± 5195	36,668 ± 1079	22,160 ± 6734	27	Rutin-isomer	RGF/GFP
A14	21.686	27,327 ± 2217	19,686 ± 1098	26,387 ± 1833	17,373 ± 2546	28	Carthamidin-7-*O*-glucuronide	RSR/WSR
A15	22.244	47,883 ± 458	43,550 ± 3103	46,438 ± 734	42,734 ± 2201	30	Isoquercetin	RSR/WSR
A16*	22.547	50,683 ± 2540	48,260 ± 9111	46,701 ± 2784	42,335 ± 4867	31	Scutellarin	RSR/WSR
A17*	22.833	331,651 ± 25,315	319,402 ± 4383	347,312 ± 21,645	332,529 ± 19,298	32	Chrysin 6-*C*-arabinoside 8-*C*-glucoside	RSR/WSR
A18*	24.98	226,469 ± 9668	226,471 ± 894	216,251 ± 1409	221,335 ± 10,953	39	Chrysin 6-*C*-glucoside 8-*C*-arabinoside	RSR/WSR
A19	25.814	50,061 ± 1477	50,078 ± 750	48,010 ± 608	49,843 ± 1839	42	Chrysin 6-*C*-glucoside 8-*C*-arabinoside isomer	RSR/WSR
A20	25.978	51,148 ± 841	50,401 ± 1777	47,781 ± 1856	49,468 ± 1588	43	Trihydroxydihydrochalcone-3'-*C*-glucoside-6'-*O*-glucoside or isomer	RSR/WSR
A21*	27.985	18,788 ± 1294	16,697 ± 1579	17,436 ± 1206	16,518 ± 1077	51	viscidulin III	RSR/WSR
A22	29.152	70,230 ± 3907	65,032 ± 10,918	69,480 ± 6047	64,205 ± 3446	54	Trihydroxy-methoxy-flavone-7-*O*-glucuronide	RSR/WSR
A23	29.656	83,912 ± 1415	68,058 ± 2439	84,110 ± 1243	74,256 ± 4306	56	6''-*O*-[(*E*)-*p*-*C*oumaroyl] genipin gentiobioside or isomer	RGF/GFP
A24*	31.704	8,378,232 ± 51,287	8,215,389 ± 176,081	8,394,519 ± 144,070	8,451,971 ± 50,374	62	Baicalin	RSR/WSR
A25*	33.149	106,929 ± 5951	109,616 ± 2943	109,496 ± 6527	110,777 ± 3170	65	Dihydrobaicalin	RSR/WSR
A26*	34.949	492,670 ± 5428	473,931 ± 8798	490,313 ± 8622	503,542 ± 16,936	67	Glychionide A	RSR/WSR
A27*	35.572	49,090 ± 2903	53,118 ± 1446	53,056 ± 2160	54,830 ± 925	69	Oroxylin A 7-*O*-*D*-glucuronide isomer	RSR/WSR
A28	36.472	77,953 ± 2731	78,722 ± 1820	80,404 ± 1401	81,515 ± 5223	70	Norwogonin-8-*O*-glucuronide	RSR/WSR
A29	37.135	111,404 ± 5959	107,409 ± 8274	112,944 ± 4394	111,880 ± 2683	71	5,7,2 '-Trihydroxy-6-methoxy-flavone-7-*O*-glucuronide	RSR/WSR
A30*	37.771	296,565 ± 6634	289,719 ± 6395	300,501 ± 8784	308,742 ± 4147	73	Chrysin-7-*O*-β-*D*-glucoronide	RSR/WSR
A31*	38.968	867,803 ± 29,230	888,166 ± 24,486	872,761 ± 42,160	864,701 ± 13,114	74	Oroxylin A-7-*O*-*D*-glucuronide	RSR/WSR
A32	40.261	105,809 ± 2200	110,273 ± 8874	99,396 ± 4418	104,173 ± 10,745	79	Kaempferol isomer	RSR/WSR
A33	41.775	62,715 ± 1942	65,939 ± 7249	59,428 ± 4275	62,811 ± 9025	80	Baicalein-6-*O*-glucuronide	RSR/WSR
A34*	42.455	2,251,622 ± 28,194	2,190,797 ± 53,988	2,233,245 ± 58,642	2,282,152 ± 67,869	82	Wogonoside	RSR/WSR
A35*	44.474	42,498 ± 1119	42,640 ± 3159	44,371 ± 2629	42,046 ± 917	83	Norwogonin	RSR/WSR
A36	45.96	14,209 ± 1530	14,729 ± 1838	13,570 ± 767	12,786 ± 755	89	Trihydroxymethoxy-flavone	RSR/WSR
A37*	46.389	40,856 ± 7758	44,247 ± 4859	46,736 ± 5216	42,855 ± 3390	90	Baicalein	RSR/WSR
A38*	50.852	11,696 ± 2652	13,188 ± 2256	13,199 ± 1046	12,781 ± 986	100	Wogonin	RSR/WSR
A39*	51.603	4568 ± 492	7010 ± 1566	5515 ± 2092	4017 ± 742	101	Chrysin	RSR/WSR
A40*	51.985	21,862 ± 2444	24,841 ± 4204	28,551 ± 3692	25,328 ± 1838	104	Oroxylin A	RGF/GFP
B1	27.031	2699 ± 66	0 ± 0	3541 ± 182	0 ± 0	NF	Unknown	RGF/GFP
B2	28.512	1826 ± 61	0 ± 0	2601 ± 68	0 ± 0	NF	Unknown	RGF/GFP
B3	28.94	3641 ± 364	0 ± 0	4342 ± 437	0 ± 0	NF	Unknown	RGF/GFP
B4	29.244	1153 ± 82	0 ± 0	1767 ± 258	0 ± 0	NF	Unknown	RGF/GFP
B5	30.151	2760 ± 105	0 ± 0	3703 ± 233	0 ± 0	NF	Unknown	RGF/GFP
B6*	32.792	401,421 ± 17,117	16,607 ± 3417	524,700 ± 35,421	17,248 ± 1792	64	Crocin I	RGF/GFP
B7	34.101	26,276 ± 1453	2689 ± 583	27,694 ± 3188	2720 ± 215	NF	Unknown	RGF/GFP
B8*	39.524	36,900 ± 2189	0 ± 0	52,355 ± 3630	0 ± 0	75	Crocin II	RGF/GFP
B9	46.324	18,539 ± 1180	0 ± 0	27,214 ± 4328	0 ± 0	NF	Unknown	RGF/GFP
B10	46.975	4724 ± 529	0 ± 0	6728 ± 1108	0 ± 0	NF	Unknown	RGF/GFP
B11	48.756	21,526 ± 2832	18,231 ± 4940	31,397 ± 6500	23,220 ± 7320	95	all-trans-Crocetin di-β-*D*-glucosyl eater	RGF/GFP
B12	49.487	87,444 ± 2497	6708 ± 1673	107,961 ± 6890	7557 ± 411	96	Crocin I isomer	RGF/GFP
B13	50.215	7982 ± 301	0 ± 0	8395 ± 771	0 ± 0	109	Neocrocin B/C/D/E	RGF/GFP
B14	54.913	1633 ± 253	2291 ± 935	2374 ± 744	3202 ± 1171	110	Crocin-III or isomer 1	RGF/GFP
B15	55.102	1173 ± 92	0 ± 0	1533 ± 391	0 ± 0	111	Crocin-III or 2 isomer	RGF/GFP
B16	55.627	2235 ± 266	3165 ± 1235	3114 ± 947	4097 ± 1285	112	Crocin-III or isomer 3	RGF/GFP
B17*	59.06	0 ± 0	1193 ± 421	0 ± 0	1140 ± 221	113	Crocetin	RGF/GFP

^*^ represents the structure identified by the reference material; ^#^ represents the structure identified by UHPLC-Orbitrap HRMS, *NF* represents not found

### Chemical variations of HQQFD from different group

**Fig. 3:**
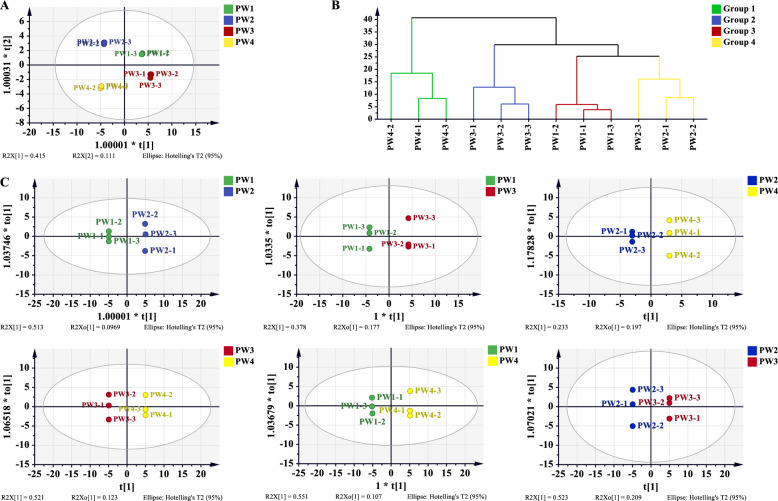
Comparison of the chemical composition of the HQQFDs among the four groups. **A** Scores Scatter plot of the four groups according to OPLS mode. **B** Cluster analysis showing variations based on different groups. **C** Pairwise comparison between different groups according to OPLS-DA

To analyze the chemical variations of HQQFD across the four groups, we utilized SIMCA-P 14.1 to perform PCA analysis (Fig. S12). However, PCA could not effectively distinguish HQQFDs among various groups. Consequently, we conducted an OPLS-DA analysis after incorporating group information and selecting four comparisons and six pairwise comparisons. These models demonstrated good predictability and reliability (R^2^X > 0.5, R^2^Y > 0.9, and Q^2^ > 0.9), as shown in Table 4, and their permutation test results were shown in Fig. S13. The HQQFD samples from different groups were well separated (Fig. 3). According to the internal inspection parameters, VIP screening revealed differences in the compounds between groups (Table S6-S12). We combined these components with a VIP > 1 and removed duplicate entries, resulting in a final total of 49 components (Table 5). These results indicate that these components significantly impact the classification of HQQFDs into different combinations, potentially serving as the main symbolic ingredients responsible for the quality differences in SR and GF before and after processing. However, whether these components relate to the therapeutic effects of ALI remains to be studied in the next phase.

**Table 4:** Parameter information of OPLS model for all efficacy indexes

Item	R^2^X	R^2^Y	Q^2^
PW1-PW2-PW3-PW4	0.931	0.997	0.904
PW1-PW2	0.515	0.996	0.934
PW1-PW3	0.555	0.999	0.920
PW2-PW4	0.531	1	0.920
PW3-PW4	0.526	0.992	0.943
PW1-PW4	0.553	0.998	0.961
PW2-PW3	0.545	0.970	0.917

**Table 5:** The main compounds selected by OPLS model

No	Peak	No	Peak	No	Peak
1	A1	18	A18	35	B3
2	A2	19	A19	36	B4
3	A3	20	A20	37	B5
4	A4	21	A22	38	B6
5	A5	22	A23	39	B7
6	A6	23	A24	40	B8
7	A7	24	A26	41	B9
8	A8	25	A27	42	B10
9	A9	26	A30	43	B11
10	A10	27	A31	44	B12
11	A11	28	A32	45	B13
12	A12	29	A34	46	B14
13	A13	30	A36	47	B15
14	A14	31	A39	48	B16
15	A15	32	A40	49	B17
16	A16	33	B1		
17	A17	34	B2		

### Results of pharmacodynamic effects

#### Histopathological observation and scoring

**Fig. 4:**
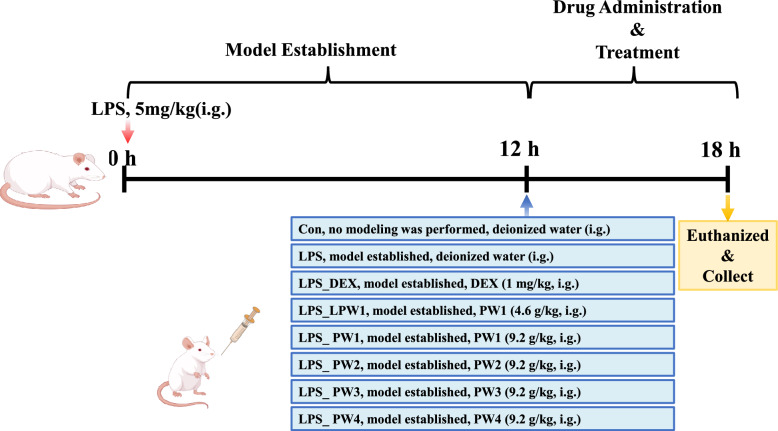
Overview of the experimental groups and treatment timeline

**Fig. 5:**
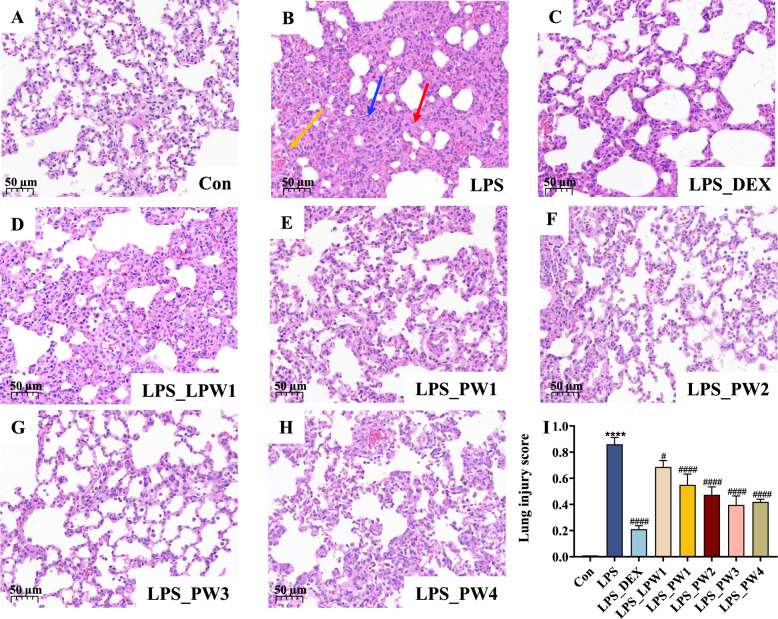
Pathological changes in lung tissues of LPS-induced ALI rats. **A-H** H&E staining of lung tissue. **I** Lung injury scoring. Values are expressed as the mean ± SD (*n* = 3), ****P < 0.0001, significant differences compared to Con; ^#^*P* < 0.05, ^####^*P* < 0.0001, significant differences compared to LPS

The animal experiments were conducted as described above, and the workflow is illustrated in Fig. 4. The H&E staining results of the lung tissues are presented in Fig. 5A-I. Histopathological findings revealed a clear pulmonary structure and minimal cellular influx in the control group, whereas ALI animals exhibited severe lung injury, including marked interstitial edema, septal thickening, hemorrhage, and inflammatory cell infiltration. The treatment group showed an opposite trend compared to the lung tissue scores. The treatment efficacies of the four combination groups were PW3 > PW4 > PW2 > PW1.

### HQQFD attenuated severity of inflammation in the lung and BALF

**Fig. 6:**
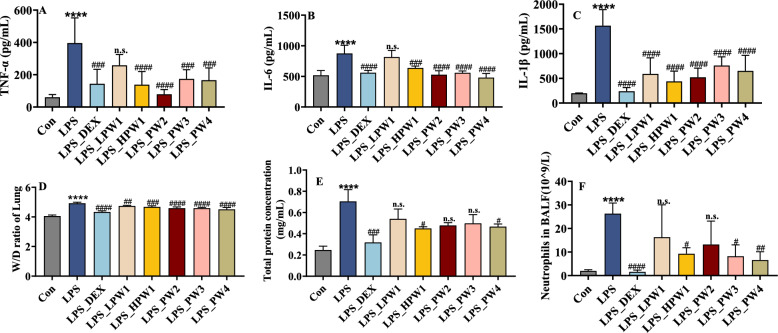
Active ingredients improved inflammatory factors and edema in lung tissue, as well as protein exudate and neutrophil content in BALF. **A-C** Inflammatory factors. **D** W/D ratio of lung. **E** Protein in BALF. **F** Neutrophil content in panel. Values are expressed as the mean ± SD (*n* = 6). n.s., not significant; ****P < 0.0001 indicates significant differences compared to Control; ^#^*P* < 0.05, ^##^*P* < 0.01, ^###^*P* < 0.005, and ^####^*P* < 0.0001 indicate significant differences compared to LPS group

To further elucidate the role of HQQFD in rats treated with LPS, we measured the expression of inflammatory cytokines, including TNF-α, IL-6, and IL-1β, with results shown in Fig. 6A-C. Compared to the control group, the expression of TNF-α, IL-6, and IL-1β in the lung tissue of the LPS group was significantly increased. Except for the LPW1 group, the other treatment groups significantly inhibited the release of TNF-α and IL-6, whereas all treatment groups significantly inhibited the release of IL-1β.

As shown in Fig. 6D, the W/D ratio in the LPS group was notably higher than that in the control group. The lung W/D ratios were significantly reduced in the 4.6 g/kg and 9.2 g/kg HQQFD groups, as well as in other treatment groups compared to the LPS group; however, there was no statistically significant difference among PW1, PW2, PW3, and PW4.

Elevated protein concentration in BALF is an important marker of injury to the pulmonary alveolar–capillary barrier. Pulmonary protein exudation was significantly reduced by PW1 and PW4 at dose of 9.2 g/kg (Fig. 6E), indicating that the treatment ameliorated alveolar–capillary barrier damage during ALI.

Neutrophils are key factors in the inflammatory response, and the neutrophil count in BALF is an important indicator of this response. As shown in Fig. 6F, the number of neutrophils in the BALF was significantly higher in LPS-induced animals. Administration of PW1, PW3, and PW4 at a dose of 9.2 g/kg, as well as DEX, significantly suppressed neutrophil accumulation.

Overall, the differences in efficacy among the various treatment groups indicated a trend toward differing treatment effects, although none reached statistical significance.

### Overall rating of the effects of HQQFD

Based on this analysis, HQQFD demonstrated therapeutic effects on LPS-induced ALI, however, the extent of improvement across various pharmacodynamic indices varied with different combinations. The inconsistencies in the degree of enhancement among the pharmacodynamic indicators were attributed to the diverse combinations of HQQFD forms. TCM is characterized by multiple components and diverse mechanisms of action, which constitutes a key rationale for conducting this study. However, different pharmacodynamic indicators may correspond to distinct phases of the disease course. Therefore, a comprehensive scoring method enables a more objective evaluation of its therapeutic efficacy. To compare the combined efficacy of the four combinations, we calculated rating scores using the AHP-EWM method. The procedures are detailed in the Supplementary Material (Tables S13–S17).

The weights assigned to the various factors were: W/D ratio of Lung (9.75%), Lung injury score (30.80%), Neutrophils in BALF (18.45%), TNF-α (11.81%), IL-6 (18.42%), IL-1β (9.09%), and Protein in BALF (1.68%). Subsequently, we calculated the composite efficacy scores of the different HQQFD combinations, the result was shown in Table 6, it can be seen the overall rating ranked as follows: PW4 > PW3 > PW2 > PW1.

**Table 6:** The results of positive treatment of efficacy indicators and the comprehensive score of efficacy indicators

Item	W/D ratio of Lung	Lung injury score	Neutrophils in BALF	TNF-α	IL-6	IL-1β	Protein in BALF	Overall rating
LPS_PW1	4.88	36.05	64.65	65.04	27.13	72.04	36.62	43.35
LPS_PW2	6.71	45.35	49.70	80.12	39.66	66.79	32.39	47.17
LSP_PW3	6.71	53.49	68.68	55.92	36.13	51.57	29.58	48.24
LPS_PW4	8.33	51.16	75.00	58.02	45.17	58.42	33.80	51.46

### Results of spectrum–effect correlation analysis

#### Pearson analysis

**Fig. 7:**
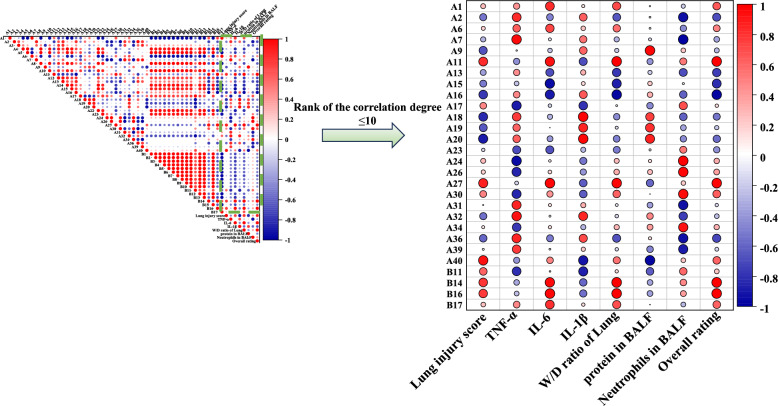
Pearson analysis results of compound peaks and pharmacodynamic index of HQQFD

We calculated the Pearson correlation coefficient for each characteristic peak in relation to the efficacy index, the result was shown in Table S18 and Fig. 7. Notably, the lung injury score exhibited significant correlations with peaks oroxylin A (A40), oroxylin A 7-*O*-*D*-glucuronide (A27), geniposide (A11), crocin-III or isomer (B14), and crocin-III or isomer (B16). Additionally, TNF-α showed significant correlation with the peaks of chlorogenic acid (A7), kaempferol isomer (A32), oroxylin A-7-*O*-glucuronide (A31), trihydroxymethoxy-flavone (A36), and 6α-hydroxygeniposide (A2). IL-6 was significantly correlated with oroxylin A 7-*O*-*D*-glucuronide (A27), crocin-III or isomer 3 (B16), crocin-III or isomer 1 (B14), geniposide (A11), and geniposidic acid (A1). Furthermore, IL-1β demonstrated significant correlations with the peaks of trihydroxydihydrochalcone-3'-*C*-glucoside-6'-*O*-glucoside or isomer (A20), chrysin 6-*C*-glucoside 8-*C*-arabinoside (A18), chrysin 6-*C*-glucoside 8-*C*-arabinoside isomer (A19), kaempferol isomer (A32), and genipin 1-gentiobioside (A9). The W/D ratio significantly correlated with peaks of crocin-III or isomer (B16), crocin-III or isomer (B14), geniposide (A11), oroxylin A 7-*O*-*D*-glucuronide (A27), and geniposidic acid (A1). The protein content in BALF was significantly correlated with genipin 1-gentiobioside (A9), trihydroxydihydrochalcone-3'-*C*-glucoside-6'-*O*-glucoside or isomer (A20), chrysin 6-*C*-glucoside 8-*C*-arabinoside isomer (A19), chrysin 6-*C*-glucoside 8-*C*-arabinoside (A18), and kaempferol isomer (A32). The neutrophil content in BALF was significantly correlated with peaks of baicalin (A24), glychionide A (A26), chrysin-7-*O*-β-*D*-glucoronide (A30), wogonoside (A34), and chrysin 6-*C*-arabinoside 8-*C*-glucoside (A17). The five peaks with the highest correlation coefficients based on overall ratings were geniposide (A11), crocin-III or isomer (B14), crocin-III or isomer (B16), oroxylin A 7-*O*-*D*-glucuronide isomer (A27), and chrysin-7-*O*-β-*D*-glucoronide (A30).

### Gray relational analysis

**Fig. 8:**
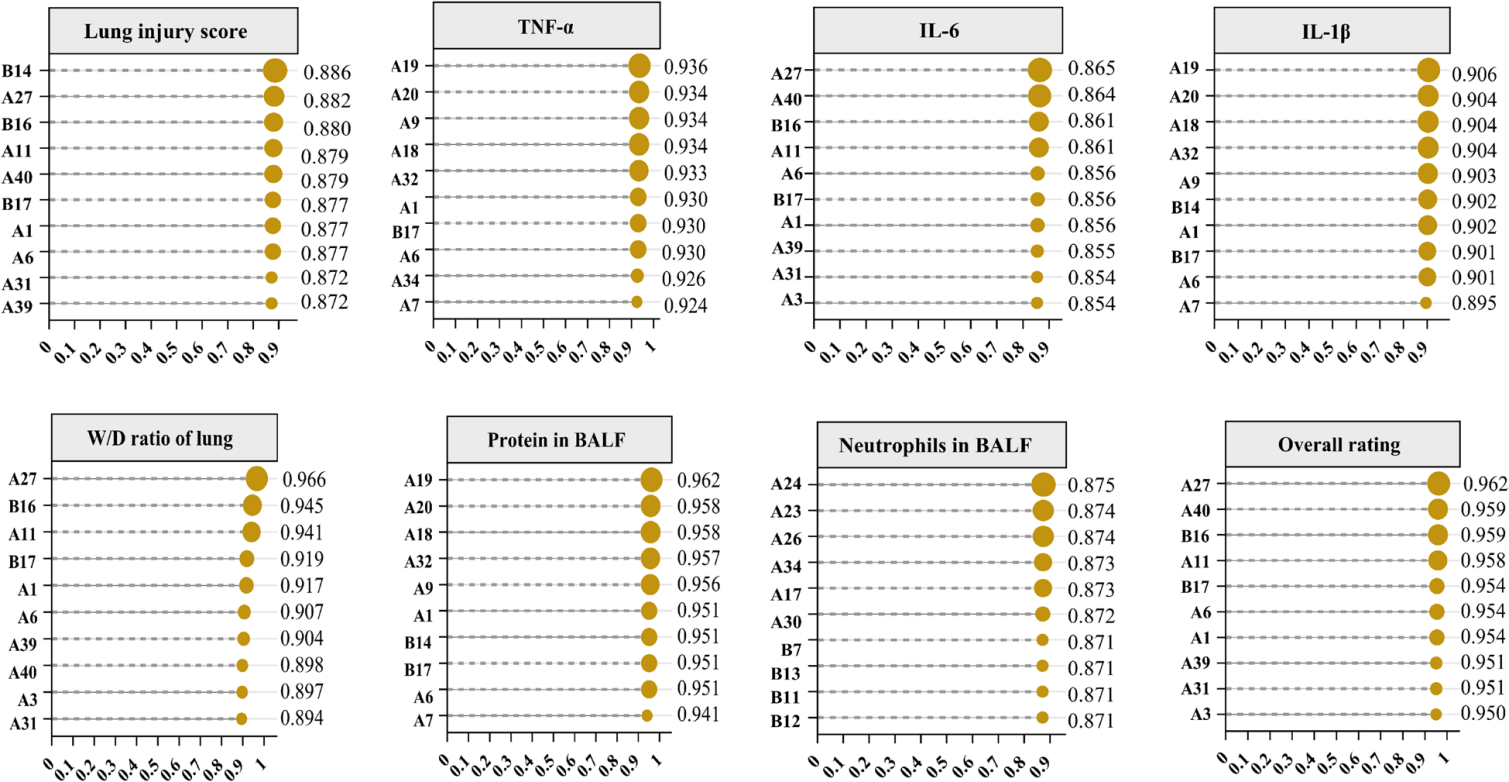
GRA analysis results of HQQFD

The pharmacodynamic indices served as the primary sequence, whereas the characteristic peak areas functioned as subsequences. The calculated results are presented in Table S19, as shown in Fig. 8, with the top ten peaks demonstrating a gray correlation degree greater than 0.8. Specifically, the lung injury score correlated significantly with peaks crocin-III or isomer crocin-III or isomer (B14), oroxylin A 7-*O*-*D*-glucuronide (A27), crocin-III or isomer (B16), geniposide (A11), and oroxylin A (A40). TNF-α showed significant correlations with chrysin 6-*C*-glucoside 8-*C*-arabinoside isomer (A19), trihydroxydihydrochalcone-3'-*C*-glucoside-6'-*O*-glucoside or isomer (A20), genipin 1-gentiobioside (A9), chrysin 6-*C*-glucoside 8-*C*-arabinoside (A18), and kaempferol isomer (A32). IL-6 was significantly associated with oroxylin A 7-*O*-*D*-glucuronide isomer (A27), oroxylin A (A40), crocin-III or isomer (B16), geniposide (A11), and neochlorogenic acid (A6). IL-1β was significantly correlated with chrysin 6-*C*-glucoside 8-*C*-arabinoside isomer (A19), trihydroxydihydrochalcone-3'-*C*-glucoside-the 6'-*O*-glucoside or isomer (A20), chrysin 6-*C*-glucoside 8-*C*-arabinoschrysin (A8), kaempferol isomer (A32), and genipin 1-gentiobioside (A9). The W/D ratio correlated significantly with oroxylin A 7-*O*-*D*-glucuronide (A27), crocin-III or isomer 2 (B16), geniposide (A11), crocetin (B17), and geniposidic acid (A1), etc. The protein content in BALF correlated significantly with chrysin 6-*C*-glucoside 8-*C*-arabinoside isomer (A19), trihydroxydihydrochalcone-3'-*C*-glucoside-6'-*O*-glucoside or isomer (A20), chrysin 6-*C*-glucoside 8-*C*-arabinoside (A18), kaempferol isomer (A32), and genipin 1-gentiobioside (A9). Neutrophil content in BALF was significantly correlated with baicalin (A24), 6''-*O*-[(*E*)-*p*-coumaroyl] genipin gentiobioside or isomer (A23), glychionide A (A26), wogonoside (A34), and chrysin 6-*C*-arabinoside 8-*C*-glucoside (A17). The top five peaks with the highest correlation coefficients, based on overall ratings, were oroxylin A 7-*O*-*D*-glucuronide (A27), oroxylin A (A40), scutellarin (B16), geniposide (A11), and crocetin (B17).

### Orthogonal partial least squares correlation analysis

**Fig. 9:**
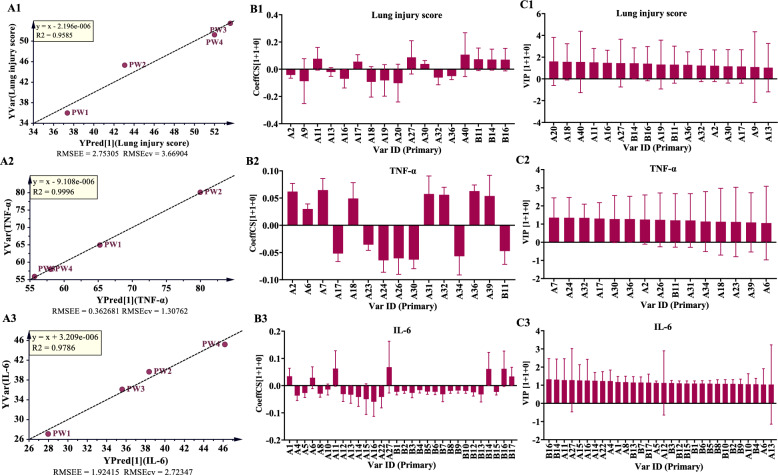

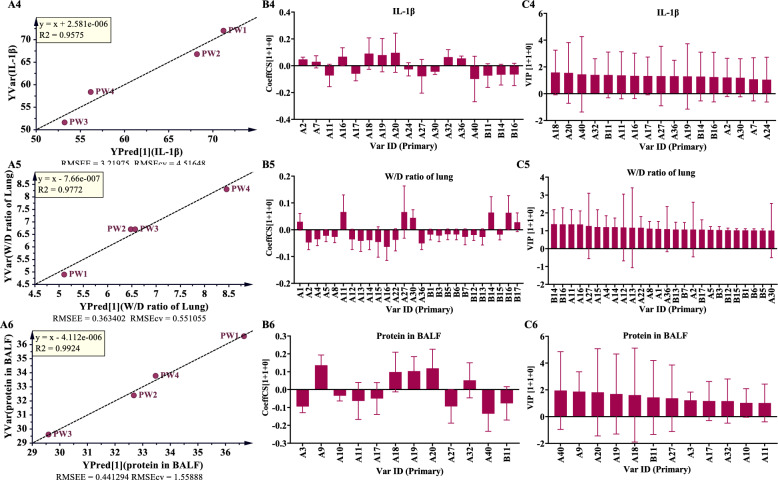

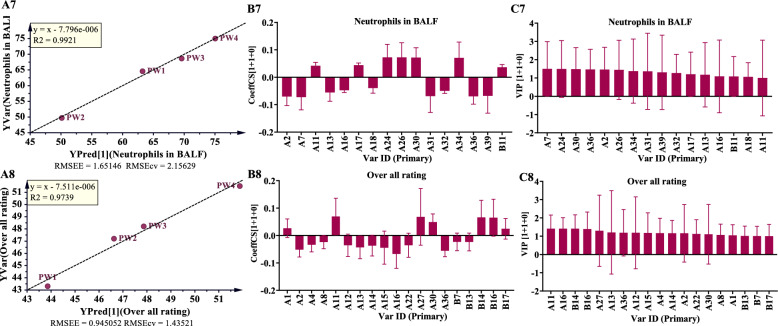
OPLS analysis results of common peaks and each pharmacodynamic index of HQQFD. **A1-A8** The OPLS linear regression. **B1-B8** The regression coefficient between the characteristic peaks and pharmacodynamic index. **C1-C8** VIP values

Simca-P 14.1 was utilized to conduct OPLS analysis, using 49 characteristic peaks as the X matrix and pharmacodynamic indices as the Y vectors. The OPLS model was evaluated using R^2^ and Q^2^, wherein R^2^ quantifies the explanatory power of the model and Q^2^ assesses its predictive power, as shown in Table 7, and the results are presented in Table S20. The comparison between observed and predicted values for the selected response diagram is depicted in Fig. 9 (A1–A8). These findings indicate that the regression models for various pharmacological effects effectively explain and predict outcomes. As illustrated in Fig. 9 (B1–B8), the regression coefficients demonsteate the positive or negative contributions of each peak to the activity. Figure 9 (C1–C8) displays peaks with VIP > 1 screened from each pharmacodynamic index. Among these, the top five peaks were geniposide (A11), scutellarin (A16), crocin-III or isomer (B14), crocin-III or isomer (B16), and Oroxylin A 7-*O*-*D*-glucuronide (A27).

**Table 7:** The parameters of OPLS mode

Item	R^2^X	R^2^Y	Q^2^
Lung injury score	0.730	0.959	0.705
W/D ratio of Lung	0.832	0.977	0.790
TNF-α	0.836	0.996	0.975
IL-6	0.808	0.979	0.829
IL-1β	0.755	0.958	0.666
Protein in BALF	0.717	0.992	0.619
Neutrophils in BALF	0.850	0.995	0.946
Overall rating	0.833	0.974	0.759

### Summary of statistical analysis

To enhance the precision and dependability of the spectrum–effect relationship investigation, we summarized the results from the extended Pearson correlation, GRA, and OPLS. As shown in Table 8, 20 compounds were identified as potential efficacy-oriented components of HQQFD. Compounds oroxylin A (A40), oroxylin A 7-*O*-*D*-glucuronide (A27), geniposide (A11), etc. were ultimately screened as the main ingredients for reducing pathological damage to lung tissue, while geniposidic acid (A1), neochlorogenic acid (A6), chlorogenic acid (A7), etc.were identified as candidate ingredients for inhibiting the release of inflammatory factors (TNF-α, IL-6, IL-1β). Additionally, crocin-III or isomer (B16), geniposide (A11), oroxylin A 7-*O*-*D*-glucuronide (A27), etc. were screened as candidate ingredients for the W/D ratio of lung. Genipin 1-gentiobioside (A9), trihydroxydihydrochalcone-3'-*C*-glucoside-6'-*O*-glucoside or isomer (A20), chrysin 6-*C*-glucoside 8-*C*-arabinoside isomer (A19), etc. were identified as candidate ingredients for protein content in BALF, while baicalin (A24), glychionide A (A26), chrysin-7-*O*-β-*D*-glucoronide (A30), etc. were identified for neutrophils in BALF. Consequently, geniposide (A11), crocin-III or isomer2 (B16), oroxylin A 7-*O*-*D*-glucuronide isomer (A27), etc. were screened as the main components for overall rating.

**Table 8:** Summary of three spectrum-effect correlation analysis methods

Efficacy index	Pearson ∩ GRA ∩ OPLS-DA	Merge deduplication
Lung injury score	A40, A27, A11, B14, B16	**A1**, **A6**, **A7**, A9, **A11**, A17, **A18**, **A19**, **A20**, A24, A26, **A27**, **A30**, A32, A34, A40, B11, B14, **B16**, **B17**
TNF-α	A7, A32, A18, A6	
IL-6	A27, B16, A11, A1, B17, A6	
IL-1β	A20, A18, A19, A32, A7	
W/D ratio of Lung	B16, A11, A27, A1, B17	
Protein in BALF	A9, A20, A19, A18, A32	
Neutrophils in BALF	A24, A26, A30, A34, A17, B11	
Overall rating	A11, B16, A27, A1, B17	

∩ : intersection; Boldfaced ingredients indicate ingredients that are associated with multiple pharmacodynamic indicators

**Fig. 10:**
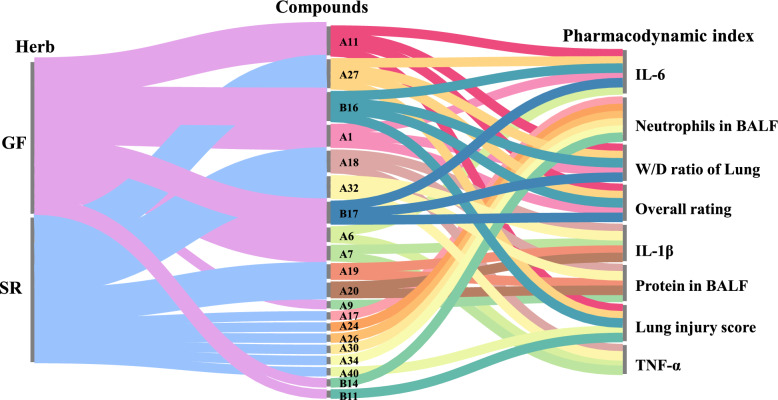
Visualization results of herb-constituent-pharmacodynamic correlation analysis

**Fig. 11:**
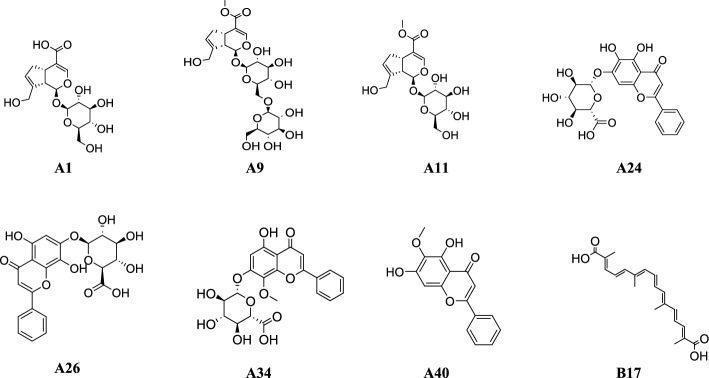
Structures of 8 efficacy-oriented components identified from lung tissues

In addition, considering the potential synergies among the multiple components and comparing their contributions, we imported the data into Cytoscape 3.8.1 for node analysis. Ingredients with a greater number of nodes contributed more significantly to efficacy. In this study, compounds with three topological features (degree, betweenness, and closeness) exceeding the median values were identified as crucial potential efficacy-oriented ingredients of HQQFD against acute lung injury (ALI). The results are shown in Table 9, with visualizations provided in Fig. 10. Among them, geniposidic acid (A1), genipin 1-gentiobioside (A9), geniposide (A11), baicalin (A24), glychionide A (A26), wogonoside (A34), oroxylin A (A40) and crocetin (B17) were detected in lung tissues in our previous study [19], the structures of these eight components are illustrated in Fig. 11. which suggests that these components are more closely related and may synergise more targets in the treatment of ALI.

**Table 9:** Network analysis result of compounds with potential ability efficacy orientation

No	Betweenness Centrality	Closeness Centrality	Degree	Source
A1	0.0155	0.3625	4	GF
A6	0.0331	0.3718	3	SR
A7^Δ^	0.0535	0.3816	3	SR
A9	0.0361	0.3718	2	SR
A11^Δ^	0.0321	0.3816	5	GF
A17	0.0082	0.3625	2	SR
A18^Δ^	0.0478	0.4028	4	GF
A19	0.0213	0.3816	3	SR
A20	0.0213	0.3816	3	SR
A24	0.0082	0.3625	2	SR
A26	0.0082	0.3625	2	SR
A27^Δ^	0.1878	0.4531	5	GF
A30	0.0082	0.3625	2	SR
A32^Δ^	0.0478	0.4028	4	SR
A34	0.0082	0.3625	2	GF
A40^Δ^	0.0305	0.3816	2	GF
B11^Δ^	0.0611	0.3816	2	GF
B14	0.0081	0.3537	2	GF
B16^Δ^	0.0321	0.3816	5	GF
B17	0.0155	0.3625	4	GF

^Δ^indicates components filtered by the median

## Discussion

HQQFD is a TCM formulation that is extensively utilized for the treatment of pulmonary and upper respiratory tract infections. Although the classic formulation for ALI has been documented, research on its concoction forms, particularly in beverage preparations, remains limited. In this study, we induced ALI using LPS and evaluated several pharmacodynamic indices, including lung histopathological scores and validation factors. Our findings indicated a positive correlation between pharmacodynamic results and dosage, with varying improvements across different combinations of HQQFD at the same dose. To assess the therapeutic effects, we employed the AHP-EAW method and ranked the composite efficacy scores as follows: PW4 > PW3 > PW2 > PW1. Although no significant differences were observed among the four groups at the same dose (*P* > 0.05), the overall rating for PW4 exceeded that of PW2, whereas PW3 outperformed PW1. This indicates that when RGF or GFP is fixed in HQQFD, the efficacy of the WSR combination is superior to that of RSR.. Furthermore, when WSR was fixed in the HQQFD, the combination with GFP appeared to be more effective than RGF. These findings, which demonstrate variations in efficacy based on combinations, align with the TCM theories, thereby enhancing our understanding of TCM concoctions. Exploring the mechanisms underlying the efficacy enhancement of Chinese herbal concoctions holds significant theoretical and clinical value, however, the complexity of these medicines presents notable challenges. Recent studies have sought to explore the relationship between constituents and pharmacodynamics through spectrum-effect correlation analysis [20, 21]. This study employs spectrum-effect correlation analysis to uncover efficacy-oriented components within the HQQFD, representing a core innovative aspect of our research.

On one hand, we conducted a chemical fingerprint of HQQFD to identify the discriminatory markers present in its various combinations, our methodology integrated UHPLC-PDA with UHPLC-Orbitrap-MS, the latter offers high efficiency, resolution, sensitivity, and robust qualitative capabilities, making it ideal for analyzing the chemical fingerprints of TCM. To mitigate the interference caused by co-elution, we systematically optimized the chromatographic separation conditions. Among these chromatographic separation factors, the type of organic phase significantly affected separation, with a 4:1 acetonitrile-methanol ratio yielding optimal results. Although the high number of components and complexity of the mixtures extended the analysis time for the method developed in this study, we anticipate that advancements in separation materials and mass spectrometry techniques will reduce this time in future investigations. Finally, the characteristic spectrum identified 57 peaks from HQQFD, and 49 differential components were screened using OPLS mode.

On the other hand, each statistical method has its strengths and limitations, making it advisable to employ multiple methods when studying spectrum-effect relationships to strengthen our conclusions. Thus, we utilized three chemometric methods for the spectrum-effect correlation analysis. Although some discrepancies arose in the active components identified by these methods, several common components were also recognized. To enhance the accuracy of our findings, we focused on the active ingredients that intersected across the three correlation methods, ensuring robust credibility, spectrum-effect correlation analysis revealed 20 compounds with pronounced efficacy-oriented attributes in HQQFD, among them, geniposidic acid (A1), genipin 1-gentiobioside (A9), geniposide (A11), baicalin (A24), glychionide A (A26), wogonoside (A34), oroxylin A (A40) and crocetin (B17) was detected in lung tissues in our previous study [19, 22]. Flavonoids from SR (baicalin, wogonoside) and iridoids/diterpenoids from GF (genipin 1-gentiobioside, crocetin) exhibit selective affinity for ALI-related hub targets identified in our prior network pharmacology study. Baicalin and wogonoside strongly bind to GSK3B, MAPK8, and IL-6 via hydrogen bonds and hydrophobic interactions, thereby inhibiting pro-inflammatory cytokine releas. Genipin 1-gentiobioside and crocetin from GF interact with AKT1 and TNF through similar binding modes, regulating cell survival and platelet activation pathways. These interactions show comparable or superior binding affinity to positive drugs like dexamethasone, confirming their functional relevance [19]. According to reports, baicalin (A24) and wogonoside (A34) demonstrated the ability to suppress TNF-α, IL-1β, and IL-6 levels and expression, both in vitro and in vivo, induce protection against inflammation during LPS-induced ALI [8, 23]. While geniposide (A11) and its metabolite genipin could pretreatment prevented LPS-induced histopathological deterioration, increased pulmonary edema, and decreased oxygenation index [10]. Additionally, crocetin (B17) could attenuate LPS-induced ALI, possessing protection involved the inhibition of inflammatory cell infiltration, reduction of pro-inflammatory mediator expression [24]. Which further supporting the validity of our screening results.

**Fig. 12:**
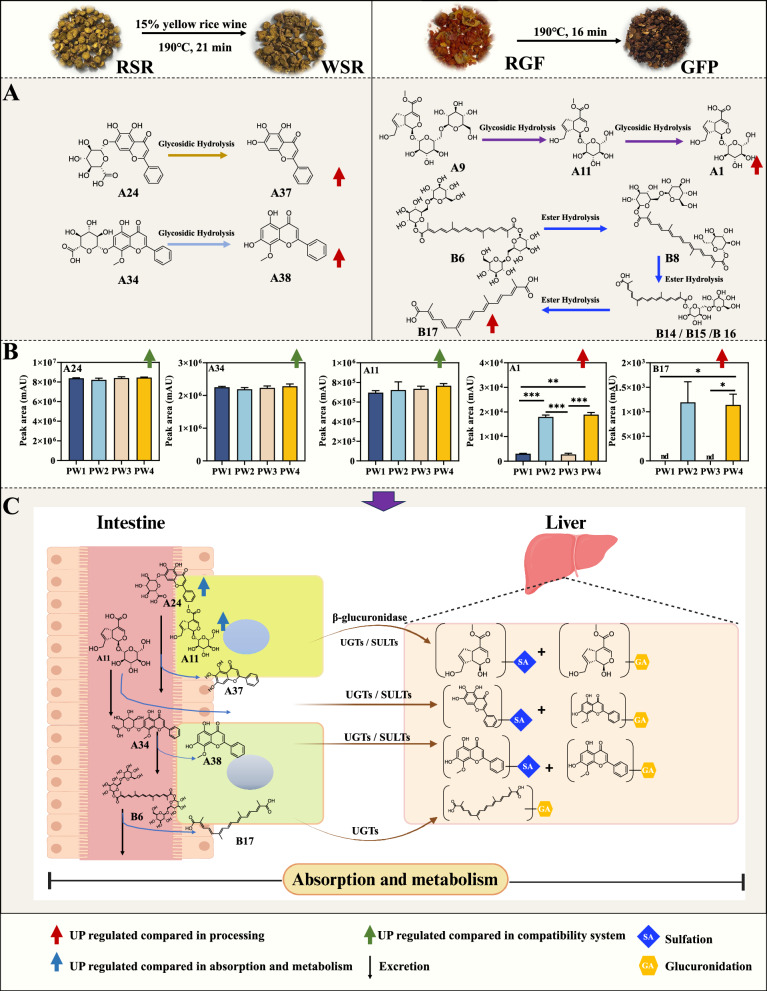
A simple schematic diagram exploring the mechanism of potentiation from the perspective of processing and compatibility (in vitro)-absorption and metabolism (in vivo) of HQQFD. **A** Possible chemical reactions of the main components during the processing of RSR into WSR and RGF into GFP. **B** Comparison results of the contents of main components in four different combinations of Huangqin Qingfei Decoction. **C** Possible metabolic reactions of the main components of HQQFD in the intestines and liver

The main efficacy-oriented components in the treatment of ALI provide the basis for investigating the mechanisms of potentiation. To facilitate understanding, we provide a schematic diagram, as shown in Fig. 12. The processing of RSR and RGF involves high-temperature heating, this heating may reduce the number of glycosyl groups in the constituents, which is a common chemical reaction—hydrolysis. This reaction can occur when RSR is transformed into WSR, leading to the conversion of baicalin (A24) to baicalein (A37) and wogonoside (A34) to wogonin (A38). Since glycosides are prone to degradation under heat, the total flavonoid content tends to decrease, but the results of studies on whether glycosides are elevated or reduced are not consistent. We speculate that this is related to the conditions of processing, when the amount of glycosides produced is greater than the amount destroyed, the results should show an increasing trend, and vice versa, a decreasing trend will be observed. The conversions of genipin 1-gentiobioside (A9) and geniposide (A11) to geniposidic acid (A1), as well as crocin I (B6) to crocetin (B17), followed this pattern [25]. The hydrolysis of glycosides can effectively reduce the number of glycosyl groups present in these components, consequently increasing their permeability in the body [22]. To analyze the content changes within the compound formula, we compared the peak areas of these components using ANOVA (Fig. 12B). The results indicated that the contents of baicalin (A24) and wogonoside (A34) in HQQFD combined with WSR (PW3 and PW4) were slightly elevated compared to those in RSR (PW1 and PW2). Which suggests that decocting with RGF enhances component conversion post-dissolution, these findings are consistent with those of our previous report [26]. Glycoside-aglycone conversion may occur during processing. The combination of RSR and GFP (PW4) has a relatively high content of aglycones components, which is consistent with the conclusion in previous studies that processing can promote the hydrolysis of glycoside components’, and it is speculated that this may be one of the reasons for its more favorable efficacy trend. Aglycones components usually have higher lipid solubility and bioavailability, and it is speculated that their absorption efficiency is better than that of glycoside precursors, which may further affect the exertion of in vivo pharmacodynamics.

Oral administration is the primary clinical application of TCM. The absorption of these medicines primarily occurs in the small intestine, where components may interact with the intestinal absorption barrier, thereby influencing their bioavailability. The absorption of active components is influenced by their physical properties and solubility, thus we obtained the absorption constants of the target components from the TCMSP official website (https://old.tcmsp-e.com/tcmsp.php), as shown in Table 10. The AlogP value represents the partition coefficient, OB indicates oral bioavailability, BBB signifies blood–brain barrier permeability, Caco-2 refers to Caco-2 permeability, and DL represents drug-likeness (compounds with OB ≥ 20%, DL ≥ 0.1, and BBB ≥ -0.3 are likely to demonstrate favorable absorption in the human body). Aglycones (baicalein, wogonin, genipin, and crocetin) have better absorption properties than their glycosidic counterparts (baicalin, wogonoside, genipin-1-gentiobioside, geniposide, and crocin I). These components can be detected in vitro and undergo interconversion in vivo, one of which is the gut, where the intestinal microbiota plays a crucial role. Numerous studies have detailed these metabolic pathways. Baicalein (A24) could facilitate the absorption of geniposide (A11) by inhibiting P-gp activity [27]. Therefore, under the same conditions, the absorption is better when the content of the glycoside component is lower, which can be verified by pharmacokinetics. Compared with other combinations, PW4 contains higher levels of aglycones (e.g., crocetin, baicalein) due to glycoside hydrolysis during processing; (2) These aglycones have higher AlogP values (e.g., crocetin AlogP = 4.58 vs crocin I AlogP = -2.72) and oral bioavailability (e.g., crocetin OB = 35.3% vs crocin I OB = 7.06%), thus having better intestinal absorption and tissue penetration; (3) The high bioavailability of key aglycones in PW4 is one of the important reasons for its more favorable anti-acute lung injury trend. In addition, the intervention of differential components in the bacterial flora is also an important direction to resolve the differences in efficacy, and it is critical to identify the strains involved in metabolism. Pharmacokinetic and intestinal flora studies of HQQFD are ongoing in our laboratory.

**Table 10:** Pharmacological and molecular properties of the main compounds

compound	CAS	MW	AlogP	OB (%)	Caco-2	BBB	DL
Baicalin	21,967-41-9	446.39	0.64	40.12	−0.85	−1.74	0.75
Wogonoside	51,059-44-0	459.41	0.21	7.07	−1.68	−2.15	0.77
Baicalein	491–67-8	270.25	2.33	33.52	0.63	−0.05	0.21
Wogonin	632–85-9	284.28	2.59	30.68	0.79	0.04	0.23
Genipin 1-gentiobioside	29,307-60-6	550.57	−4.00	45.58	−3.29	−5.43	0.83
Geniposide	24,512-63-8	388.41	−2.25	14.64	−1.70	−2.61	0.44
Geniposidic acid	27,741-01-1	374.38	−2.50	19.59	−2.15	−2.7	0.41
genipin	6902-77-8	226.25	−0.50	26.06	−0.37	−0.98	0.10
Crocin I	94,238-00-3	977.08	−2.72	7.06	−4.64	−6.43	0.12
Crocetin	27,876-94-4	328.44	4.58	35.3	0.54	−0.83	0.26

Additionally, the liver is rich in metabolic enzymes and an important site for component metabolism. Phase II metabolism, including glucuronidation catalyzed by the enzyme UDP-glucuronosultransferase, and sulfation catalyzed by sulfatransferase [28, 29], resulting in its conjugated metabolites, as shown in Fig. 12C. Thus, baicalin (A24), wogonoside (A34), genipin-1-gentiobioside (A9), geniposide (A11), and crocin I (B6) are converted to glycosides (baicalein, wogonin, genipin, and crocetin) by the intestinal microbiota for absorption and potentially transform back into glucuronidase or sulfatase forms. As a result, the ingredients are mainly present in the body in the form of the metabolized components mentioned above, and as such, they may be the key components in the exertion of the medicinal effects and are also important in generating the differences in efficacy. Ultimately, differences in this link also arise from the absorption link.

It is important to acknowledge that unscreened ingredients are not necessarily ineffective, they may still exhibit efficacy but contribute less significantly to differences in efficacy. In addition, this study focused solely on small molecules, omitting larger molecular weight components, such as sugars and proteins, which also play significant roles in HQQFD [30]. Although these larger components may not be readily absorbed, they undoubtedly contribute to the overall efficacy of the concoction, potentially through mechanisms such as micelle formation and aggregation in the intestines. This finding highlights an important avenue for future research. In summary, the findings presented here underscore the material basis of HQQFD and offer valuable insights into its mechanisms of action, suggesting potential directions for future investigations.

## Conclusion

Based on the principle of “effectiveness” and “compatible environment”, a new strategy for screening and evaluating efficacy-oriented components of HQQFD was proposed by combining the spectrum-effect relationship. To the best of our knowledge, this study is the first to establish a spectrum-effect relationship between HQQFD and efficacy, comparing differences between RSR and WSR, as well as RGF and GFP in compounds of HQQFD. Ultimately, 20 compounds (geniposidic acid, neochlorogenic acid, chlorogenic acid, genipin 1-gentiobioside, geniposide, chrysin 6-*C*-arabinoside 8-*C*-glucoside, chrysin 6-*C*-glucoside 8-*C*-arabinoside, chrysin 6-*C*-glucoside 8-*C*-arabinoside isomer, trihydroxydihydrochalcone-3'-*C*-glucoside-6'-*O*-glucoside or isomer, baicalin, glychionide A, oroxylin A 7-*O*-*D*-glucuronide, chrysin-7-*O*-β-*D*-glucoronide, kaempferol isomer, wogonoside, oroxylin A, all-trans-crocetin di-β-*D*-glucosyl eater, crocin-III isomer1, crocin-III or isomer2, crocetin) were screened as highly correlated with efficacy, and thus show promise as efficacy-oriented component indices in HQQFD. In conclusion, this work offers a viable strategy for identifying the pharmacodynamic material basis of TCM under processing and compatibility conditions.

## Supplementary Information

Supplementary file 1.

## Data Availability

No datasets were generated or analysed during the current study.

## Abbreviations

ALI: Acute lung injury
ARDS: Acute respiratory distress syndrome
ANOVA: Analysis of variance
AHP-EWM: Analytic Hierarchy Process-Entropy Weight method
BCA: Bicinchoninic acid
BBB: Blood–brain barrier permeability
BALF: Bronchoalveolar lavage fluid
CCA: Canonical correlation analysis
DEX: Dexamethasone acetate tablets
FGP: Fructus Gardeniae Praeparatus
GF: Gardeniae fructus
GCA: Gray correlation analysis
GRA: Gray relational analysis
H&E: Hematoxylin and eosin
HQQFD: Huangqin Qingfei decoction
ICU: Intensive care unit
LPS: Lipopolysaccharide
LSD: Least significant difference
OB: Oral bioavailability
OPLS: Orthogonal partial least squares
PLS: Partial least squares
QC: Quality control
RSD: Relative standard deviations
SR: Scutellariae Radix
RGF: Raw Gardeniae Fructus
RSR: Raw Scutellariae Radix
SVM: Support vector machine
TIC: Total ion chromatogram
TCM: Traditional Chinese medicine
UHPLC-Orbitrap HRMS: Ultra-high performance liquid chromatography-Orbitrap high-resolution mass spectrometry
VIP: Variable importance in projection
W/D ratio: Wet-to-dry weight ratio
