# Supplementary material for: Interpretation of the efficacy-oriented components of decoction pieces in compounds based on the spectrum-effect relationship: Huangqin Qingfei decoction as an example
Source: Chin Med. 2026 Feb 9;21:66. doi: 10.1186/s13020-026-01341-z (PMC12888459; doi:10.1186/s13020-026-01341-z)
Supplement: Supplementary file 1 [file 13020_2026_1341_MOESM1_ESM.docx]

# Interpretation of the efficacy-oriented components of Decoction Pieces in compounds based on the spectrum-effect relationship: Huangqin Qingfei Decoction as an example

*Corresponding author. Cun Zhang, Tel.: +86-010-64032658

*E-mail addresses*: zhc95@163.com (C. Zhang)

# Table of Contents

1. **Table S1** Identification of compounds in PW1 by UPLC-Orbitrap HRMS
2. **Table S2** Identification of compounds in PW2 by UPLC-Orbitrap HRMS.
3. **Table S3** Identification of compounds in PW3 by UPLC-Orbitrap HRMS
4. **Table S4** Identification of compounds in PW4 by UPLC-Orbitrap HRMS
5. **Table S5** Method Validation of characteristic spectrum
6. **Table S6** The difference compounds among PW1, PW2, PW3, and PW4
7. **Table S7** The difference compounds between PW1 and PW2
8. **Table S8** The difference compounds between PW1 and PW3
9. **Table S9** The difference compounds between PW2 and PW4
10. **Table S10** The difference compounds between PW1 and PW
11. **Table S11** The difference compounds between PW2 and PW3
12. **Table S12** The difference compounds between PW3 and PW4
13. **Table S13** Result of the pharmacodynamic study
14. **Table S14** Judgment matrix of AHP assessment factors
15. **Table S15** Result of AHP analysis
16. **Table S16** Result of EWM analysis
17. **Table S17** Combined weight results for each index
18. **Table S18** Pearson analysis results of characteristic peaks and each pharmacodynamic index of HQQFD
19. **Table S19** Gray relational analysis results of characteristic peaks and each pharmacodynamic index of HQQFD
20. **Table S20** OPLS-DA analysis results of characteristic peaks and each pharmacodynamic index of HQQFD
21. **Fig. S1** Optimization of UPLC separation conditions (column brands) of HQQFD
22. **Fig. S2** Optimization of UPLC separation conditions (types of acids in the aqueous phase) of HQQFD
23. **Fig. S3** Optimization of UPLC separation conditions (organic phase) of HQQFD
24. **Fig. S4** Optimization of UPLC separation conditions (acid concentration) of HQQFD
25. **Fig. S5** Optimization of UPLC separation conditions (flow rate) of HQQFD
26. **Fig. S6** Optimization of UPLC separation conditions (column temperature) of HQQFD
27. **Fig. S7** Total ion chromatogram (TIC) of HQQFD.
28. Fig. S8 Identification results of baicalin by mass spectrometry
29. Fig. S9 Identification results of baicalein by mass spectrometry
30. Fig. S10 Identification results of geniposide by mass spectrometry
31. Fig. S11 Identification results of C-I by mass spectrometry
32. **Fig. S12** PCA analysis of chemical variations of HQQFD across PW1, PW2, PW3 and PW4
33. **Fig. S13** Permutation test results (200 iterations) of OPLS-DA models for different comparison groups
34. The calculation procedure of AHP-EWM

## **Table S1** Identification of compounds in PW1 by UPLC-Orbitrap HRMS

| **No** | **t_R_**  **(min)** | **Formula** | **Selection ion** | **Measured mass (m/z)** | **Error (ppm)** | **Fragmentations** | **Identification** | **Source** |
| --- | --- | --- | --- | --- | --- | --- | --- | --- |
| 1 | 1.37 | C_7_H_12_O_6_ | [M-H]- | 191.055 | -3.14 | 191.0551, 184.6895, 179.6002, 173.0081, 142.6267, 129.0187 | Quinic acid | RGF |
| 2 | 2.45 | C_16_H_22_O_11_ | [M-H]- | 389.1066 | -4.63 | 389.1064, 303.0144, 273.8625, 227.0545, 209.0443, 183.0651, 165.0547, 147.0443, 139.0392 | Deacetylasperulosidic acid | RGF |
| 3 | 3.13 | C_16_H_22_O_10_ | [M-H]- | 373.1118 | -4.56 | N/A | Gardoside | RGF |
| 4 | 3.30 | C_16_H_24_O_11_ | [M-H]- | 391.1225 | -3.84 | 391.1232, 357.5748, 229.0714, 185.0814, 156.4031, 140.1040 | Shanzhiside isomer | RGF |
| 5 | 3.66 | C_9_H_8_O_4_ | [M-H]- | 179.034 | -2.79 | 179.0340, 161.0235, 151.0394, 134.9872, 133.0287, 122.0368 | Caffeic acid | RSR |
| 6 | 3.95 | C_17_H_26_O_11_ | [M-H]- | 405.138 | -4.20 | N/A | Shanzhiside methyl ester | RGF |
| 7 | 4.16 | C_7_H_6_O_4_ | [M-H]- | 153.0185 | -1.96 | 153.0185, 109.0289 | Protocatechuic acid | RSR |
| 8 | 4.16 | C_16_H_24_O_11_ | [M-H]- | 391.1223 | -4.35 | 391.1227, 369.0520, 260.1431, 229.0706, 193.0498, 185.0810, 179.0549, 167.0705, 149.0600, 127.0394, 101.0238 | Shanzhiside | RGF |
| 9 | 4.33 | C_16_H_22_O_10_ | [M-H]- | 373.1118 | -4.56 | N/A | Geniposidic acid | RGF |
| 10 | 5.32 | C_16_H_18_O_9_ | [M-H]- | 353.0858 | -4.25 | 353.0854, 191.0550, 179.0340, 173.0444, 135.0444, 127.0393, 930340, 85.0289 | Chlorogenic acid | RSR |
| 11 | 5.48 | C_17_H_24_O_11_ | [M+HCOO]- | 449.1272 | -5.12 | 449.1200, 343.8275, 327.5110, 300.4862, 273.6779, 263.9525, 227.3024, 205.7340, 185.3020, 138.9965, 118.8303, 105.5002 | 6α-hydroxygeniposide | RGF |
| 12 | 6.93 | C_17_H_24_O_11_ | [M+HCOO]- | 449.1273 | -4.90 | 403.1227, 371.0963, 241.0705, 223.0600, 205.0493, 191.0338, 177.0546, 161.0235, 133.0288, 127.0394, 121.0288, 109.0288, 101.0238 | Gardenoside | RGF |
| 13 | 6.98 | C_16_H_24_O_10_ | [M-H]- | 375.1275 | -4.27 | 375.1280, 335.8841, 244.9416, 227.2939, 213.0759, 195.1019, 179.0549, 169.0860, 165.2384, 151.0756, 133.0654, 125.0601, 119.0344, 107.0496, 101.0237 | Mussaenosidic acid | RGF |
| 14 | 7.22 | C_16_H_26_O_8_ | [M-H]- | 345.1533 | -4.64 | 345.1176, 315.3869 | Jasminoside B | RGF |
| 15 | 8.93 | C_17_H_24_O_11_ | [M-H]- | 403.1223 | -4.22 | 403.1601, 338.9369, 319.6859, 269.0771, 227.3033, 191.0550 | 6β-hydroxygeniposide | RGF |
| 16 | 9.37 | C_16_H_26_O_8_ | [M-H]- | 345.1535 | -4.06 | 345.1539, 307.4010, 179.0549, 161.0448, 153.0912, 149.0445, 125.0600, 113.0237, 101.0238 | Picrocrocinic acid | RGF |
| 17 | 10.74 | C_16_H_18_O_9_ | [M-H]- | 353.0855 | -5.10 | N/A | Neochlorogenic acid | RGF |
| 18 | 11.65 | C_16_H_18_O_9_ | [M-H]- | 353.0857 | -4.53 | N/A | Chlorogenic acid | RGF |
| 19 | 12.12 | C_7_H_6_O_4_ | [M-H]- | 153.0185 | -1.96 | 153.0189, 143.6439, 121.7816, 109.0288, 81.0343 | Protocatechuic acid-isomer | RSR |
| 20 | 12.42 | C_15_H_12_O_7_ | [M-H]- | 303.049 | -4.95 | 303.0493, 285.0398, 275.0548, 259.0601, 241.0487, 217.0495, 1930495, 177.0183, 149.0235, 133.0289, 125.0236, 107.0132 | 3,6,7,2',6 '-pentahydroxyflavanones | RSR |
| 21 | 12.54 | C_23_H_34_O_15_ | [M-H]- | 549.1794 | -4.55 | 595.1549, 505.1222, 475.1130, 453.5304, 385.0806, 355.0704, 299.0806, 255.0541, 225.0764, 207.0661, 166.5319, 147.0443, 139.0467, 123.0445, 101.0238 | Genipin 1-gentiobioside | RGF |
| 22 | 13.00 | C_17_H_22_O_10_ | [M-H]- | 385.1114 | -5.45 | 385.1113, 325.0919, 247.0599, 223.0598, 205.0493, 190.0259, 175.0025, 164.0468, 71.0133 | Sinapyglucoside | RGF |
| 23 | 13.55 | C_17_H_24_O_10_ | [M+HCOO]- | 433.1323 | -5.31 | 433.0763, 387.2028, 301.0337, 271.8911, 225.0757, 207.0651, 195.0290, 175.0393, 153.0915, 147.0443, 123.0445, 101.0238 | Geniposide | RGF |
| 24 | 15.25 | C_15_H_10_O_7_ | [M-H]- | 301.0334 | -4.98 | 301.0340, 283.0235, 257.0438, 229.0495, 215.0341, 201.0548, 193.0131, 185.0613, 169.0128, 161.0235, 155.0344, 151.0028, 147.0079, 139.0349, 133.0284, 125.0237, 121.0288, 107.0132 | Quercetin | RSR |
| 25 | 15.51 | C_26_H_28_O_14_ | [M-H]- | 563.1375 | -4.62 | 53.1375, 545.1279, 527.1161, 503.1176, 485.1067, 473.1063, 455.0957, 443.0959, 425.0852, 413.0854, 395.0748, 383.0752, 365.0648, 353.0647, 335.0548, 325.0702, 297.0753, 283.0597, 268.0725, 233.0445, 191.0339, 161.0235, 135.0443, 117.0339 | Schaftoside | RSR |
| 26 | 15.63 | C_16_H_26_O_7_ | [M-H]- | 375.164 | -4.00 | 285.1761, 238.6485, 220.6559, 167.1068, 161.0447, 152.0836, 132.065, 119.0343, 113.0238, 101.0240 | Picrocrocin | RGF |
| 27 | 17.73 | C_27_H_30_O_16_ | [M-H]- | 609.1472 | 2.63 | 609.1429, 575.6957, 462.4324, 343.0443, 300.0260, 271.0234, 255.0286, 243.0286, 227.0337, 199.0389, 178.9974, 151.0029, 135.0080, 107.0132 | Rutin or isomer | RGF |
| 28 | 17.97 | C_21_H_20_O_12_ | [M-H]- | 463.0855 | -4.75 | 463.0888, 441.7891, 407.8214, 372.4014, 322.1273, 308.2899, 287.0546, 269.0441, 241.0495, 225.0544, 193.0130, 181.0132, 166.9976, 153.0185, 139.0029, 123.0081, 119.0495, 113.0237 | Carthamidin-7-*O*-glucuronide | RSR |
| 29 | 18.62 | C_27_H_30_O_16_ | [M-H]- | 609.1426 | -4.92 | 609.1429, 391.7841, 343.0429, 300.0260, 271.0234, 255.286, 243.0286, 227.0337, 211.0391, 178.99886, 151.0028, 135.0080, 108.0210 | Rutin or isomer | RGF |
| 30 | 19.33 | C_21_H_20_O_12_ | [M-H]- | 463.0856 | -4.53 | 463.0859, 300.0261, 287.0441, 271.0234, 255.0286, 243.0286, 227.0340, 199.0391, 178.9976, 151.0029, 121.0287, 107.0131 | Isoquercetin | RGF |
| 31 | 19.45 | C_21_H_18_O_12_ | [M-H]- | 461.0698 | -4.77 | 794.7151, 461.0675, 362.2463, 285.0391, 267.0278, 239.0342, 211.0393, 136.9873, 113.0239 | Scutellarin | RSR |
| 32 | 19.70 | C_26_H_28_O_13_ | [M-H]- | 547.1423 | -5.30 | 547.1430, 529.1327, 487.1229, 457.1117, 427.1010, 409.0907, 379.0808, 367.0806, 349.0701, 337.0704, 321.0752, 309.0755, 295.0595, 281.0808, 267.0651, 252.0779, 227.2951, 191.0342, 163.0392, 145.0286, 119.0496 | Chrysin 6-*C*-arabinoside 8-*C*-glucoside | RSR |
| 33 | 20.00 | C_23_H_24_O_13_ | [M-H]- | 507.1118 | -4.14 | 507.1136, 492.0885, 345.0598, 330.0365, 315.0131, 287.0180, 269.0075, 233.0076, 217.0125, 178.9970, 164.9819, 149.0235, 136.9868, 110.0001 | Viscidulin III-2′-*O*-glucoside | RSR |
| 34 | 20.15 | C_16_H_26_O_8_ | [M-H]- | 345.1537 | -3.48 | 345.0235, 327.0131, 301.0346, 283.0234, 271.0227, 255.0282, 241.0132, 237.0028, 233.0082, 211.0387, 199.0396, 193.0132, 175.0029, 163.0025, 151.0029, 147.0078, 125.0237, 107.0132 | Jasminoside G | RGF |
| 35 | 20.52 | C_21_H_20_O_12_ | [M-H]- | 463.0856 | -4.53 | 348.4416, 287.0544, 261.3289, 241.0497, 227.2994, 181.0136, 175.0388, 160.0157, 153.0184, 139.0031；125.0237；117.0495；113.0240 | Carthamidin 7-*O*-glucuronide isomer | RGF |
| 36 | 21.73 | C_15_H_10_O_7_ | [M-H]- | 301.0337 | -3.99 | 301.0343, 283.0244, 273.0399, 257.0448, 229.0401, 227.2964, 217.0133, 215.0338, 201.0185, 187.0391, 166.9979, 151.0031, 139.0030, 133.0291, 129.0239, 119.0495 | 3,5,7,2 ',6 '-Pentahydroxy flavones | RGF |
| 37 | 21.78 | C_29_H_36_O_15_ | [M-H]- | 623.1943 | -5.30 | 623.1949, 607.2066, 580.5847, 461.1640, 315.1071, 297.0962, 227.2986, 206.5672, 179.0340, 161.0234, 153.0548, 133.0287, 113.0237 | Verbascoside | RSR |
| 38 | 21.85 | C_23_H_24_O_12_ | [M-H]- | 491.1169 | -4.28 | 491.1191, 461.0694, 406.6892, 371.0753, 353.0651, 338.0421, 329.0650, 315.0494, 300.0259, 285.0392, 271.0232, 255.0284, 227.0336, 204.0182, 180.0054, 164.9819, 150.0311, 133.0287, 110.0003 | Trihydroxy-dimethoxy-flavone glucoside | RSR |
| 39 | 21.85 | C_26_H_28_O_13_ | [M-H]- | 547.1426 | -4.75 | 547.1429, 529.1331, 511.1229, 487.1216, 469.1124, 457.1118, 439.1016, 427.1013, 421.0911, 397.0910, 379.0792, 367.0805, 349.0700, 337.0703, 309.0755, 293.0806, 281.0805, 267.0652, 252.0780, 235.0751, 203.0343, 191.0338, 163.0394, 145.0288, 119.0496 | Chrysin 6-*C*-glucoside 8-*C*-arabinoside | RSR |
| 40 | 22.01 | C_25_H_24_O_12_ | [M-H]- | 515.1167 | -4.46 | 515.1151, 452.9803, 411.1092, 353.0858, 335.0757, 331.1978, 255.0651, 205.0493, 191.0550, 179.0339, 173.0445, 161.0235, 155.0341, 135.0444, 127.0394 | 3,4-Dicaffeoyl quinic acid | RGF |
| 41 | 22.25 | C_25_H_24_O_12_ | [M-H]- | 515.117 | -3.88 | 353.0218, 335.3481, 249.2005, 227.3014, 205.93.64, 173.0446, 151.0029, 135.0446, 125.0238, 113.0232 | 3,5-Dicaffeoyl quinic acid | RGF |
| 42 | 22.70 | C_26_H_28_O_13_ | [M-H]- | 547.1427 | -4.57 | 547.1429, 529.1331, 511.1229, 487.1216, 469.1124, 457.1118, 439.1016, 427.1013, 421.0911, 397.0910, 379.0792, 367.0805, 349.0700, 337.0703, 309.0755, 293.0806, 281.0805, 267.0652, 252.0780, 235.0751, 203.0343, 191.0338, 163.0394, 145.0288, 119.0496 | Chrysin 6-*C*-glucoside 8-*C*-arabinoside isomer | RSR |
| 43 | 22.87 | C_27_H_34_O_14_ | [M-H]- | 581.1843 | -4.82 | N/A | Trihydroxydihydrochalcone-3'-*C*-glucoside-6'-*O*-glucoside or isomer | RSR |
| 44 | 23.03 | C_16_H_12_O_7_ | [M-H]- | 315.0496 | -2.86 | 315.0497, 300.0262, 165.9898, 153.9899, 137.9952, 129.0186, 110.0003 | Tetrahydroxymethoxy-flavone | RSR |
| 45 | 23.46 | C_29_H_36_O_15_ | [M-H]- | 623.1942 | -5.46 | 623.1948, 461.1643, 389.1595, 315.1076, 297.0978, 269.0444, 225.1210, 208.0732, 197.0345, 161.0235, 133.0287, 113.0238 | Isoacteoside | RSR |
| 46 | 23.58 | C_21_H_20_O_9_ | [M-H]- | 415.1008 | -5.06 | 415.1015, 397.0900, 325.0703, 307.0598, 295.0596, 267.0648, 253.0493, 223.0751, 181.0646, 145.0287, 121.0287 | Chrysin 8-*C*-glucoside | RSR |
| 47 | 23.59 | C_21_H_20_O_9_ | [M-H]- | 415.101 | -4.58 | 415.1014, 398.0407, 325.0704, 295.0596, 267.0649, 253.0493, 233.0754, 195.0805, 145.0287, 121.0288 | Chrysin 6-*C*-glucoside | RSR |
| 48 | 23.70 | C_21_H_20_O_10_ | [M-H]- | 431.0961 | -3.94 | 431.0959, 385.0235, 367.0121, 269.0442, 241.0495, 218.9965, 197.0602, 184.0523, 161.0236, 139.0392, 110.0003 | Apigenin 7-glucoside | RSR |
| 49 | 24.29 | C_22_H_22_O_10_ | [M-H]- | 445.1115 | -4.49 | 445.1114, 430.0891, 392.1377, 330.0963, 283.0600, 269.0443, 251.0340, 239.0343, 223.0391, 195.0445, 165.0183, 136.9876, 117.0338, 113.0241, 85.0291 | Oroxylin A 7-*O*-*D*-glucuronide | RSR |
| 50 | 24.40 | C_16_H_12_O_7_ | [M-H]- | 315.0496 | -2.86 | 315.0491, 300.0259, 251.0168, 2272973, 206.4968, 151.0027, 107.0132 | Tetrahydroxymethoxy flavone | RSR |
| 51 | 25.54 | C_17_H_14_O_8_ | [M-H]- | 345.0593 | -5.22 | 345.0596, 330.0366, 315.0131L287.0192L283.0241, 269.0090, 243.0287, 233.0086, 215.0338, 201.0543, 178.9980, 164.9820, 151.0031, 149.0235, 139.0030, 125.0237, 121.0287, 110.0003 | Viscidulin III | RSR |
| 52 | 25.94 | C_22_H_36_O_12_ | [M-H]- | 537.216 | -4.28 | 537.1940, 473.7653, 421.0036, 401.5541, 375.4704, 369.1057, 355.0912, 311.0758, 284.0311, 251.0547, 227.2995, 209.0440, 205.0494, 190.0260, 176.0114, 167.1067, 152.0106, 137.0236, 123.0444, 108.0210, 101.0239 | Jasminoside Q | RGF |
| 53 | 26.04 | C_15_H_10_O_6_ | [M-H]- | 285.0388 | -3.86 | 285.0389, 267.0286, 257.0438, 239.0336, 223.0385, 213.0544, 195.0439, 185.0597, 166.9977, 157.0652, 143.0493, 137.0236, 117.0339 | Kaempferol or isomer | RSR |
| 54 | 26.07 | C_22_H_20_O_12_ | [M-H]- | 475.0853 | -5.05 | 429.0120, 413.9885, 398.9651, 370.9706, 342.9755, 315.0133, 299.0547, 284.0312, 270.9911, 227.0332, 165.9899, 112.9849 | Trihydroxy-methoxy-flavone-7-*O*-glucuronide | RSR |
| 55 | 26.50 | C_16_H_12_O_7_ | [M-H]- | 315.0496 | -2.86 | 315.0497, 300.1819, 272.0321, 243.4700, 230.1238, 227.0338, 225.0547, 183.0439, 165.0185, 149.0236, 139.0395, 124.0159, 93.0340 | Tetrahydroxymethoxy-flavone | RSR |
| 56 | 26.61 | C_32_H_40_O_17_ | [M-H]- | 695.2147 | -5.75 | 695.2153, 649.1989, 469.1326, 451.1209, 409.1123, 367.1014, 325.0920, 307.0818, 265.0702, 225.0755, 207.0650, 163.0391, 145.0286, 123.0444, 101.0238 | 6''-*O*-[(*E*)-*p*-Coumaroyl] genipin gentiobioside or isomer | RGF |
| 57 | 26.68 | C_31_H_32_O_16_ | [M-H]- | 659.158 | -4.85 | 659.1930, 622.2500, 534.8969, 517.8245, 483.1454, 421.0231, 397.2425, 337.0889, 319.0784, 296.4605, 233.0644, 227.3046, 191.0551, 173.0445, 153.0550, 135.0445；123.0443 | 3,5-Di-*O*-Caffeoyl-4-*O*-(3-hydroxy-3-methyl) glutaroylquinic acid | RGF |
| 58 | 27.21 | C_27_H_28_O_13_ | [M-H]- | 559.1428 | -4.29 | 559.1427, 541.1326, 523.1218, 485.1067, 439.1011, 359.0747, 365.0650, 323.0753, 295.0598, 267.0651, 219.0287, 191.0338, 173.0448 | 3-*O*-Sinapoyl-5-*O*-caffeoylquinic acid | RGF |
| 59 | 27.51 | C_34_H_44_O_19_ | [M-H]- | 755.2352 | -6.22 | 755.2262, 625.1853, 529.1533, 511.1413, 427.1226, 403.0799, 367.1015, 325.0915, 299.1266, 265.0702, 241.0494, 223.0599, 207.0650, 205.0494, 191.0339, 190.0260, 175.0026, 164.0470, 149.0235, 147.0443, 12300444, 101.0238 | 6''-*O*-[trans-Sinapoyl] genipin gentiobioside | RGF |
| 60 | 27.75 | C33H42O18 | [M-H]- | 725.2255 | -5.24 | 725.2141, 665.6073, 647.0472, 549.0663, 531.0546, 408.0460, 369.0597, 347.4554, 309.0394, 279.0139, 269.0442, 253.0500, 249.0029, 239.0183, 233.0072, 223.0600, 221.0081, 205.0496, 193.0134, 178.9980, 164.8353, 149.0238, 123.0081 | 6''-*O*-[trans-Feruloyl] genipin gentiobioside | RGF |
| 61 | 27.99 | C_27_H_36_O_12_ | [M-H]- | 551.2109 | -3.63 | 551.2083, 507.6577, 471.0822, 385.1127, 325.0913, 311.0757, 295.0809, 265.0703, 250.0469, 237.0755, 223.0600, 205.0494, 190.0260, 175.0027, 164.0469, 149.0235, 135.0443, 121.0288, 101.0239 | 6'-*O*-trans-Sinapoyljasminoside L | RGF |
| 62 | 28.89 | C_21_H_18_O_11_ | [M-H]- | 445.0748 | -5.17 | 445.1133, 377.0211, 283.0603, 269.0444, 239.0336, 225.0547, 195.0444, 113.0238 | Baicalin | RSR |
| 63 | 29.14 | C_27_H_28_O_13_ | [M-H]- | 559.1425 | -4.83 | 559.1418, 515.1150, 487.0435, 455.0955, 397.1135, 353.0228, 329.0229, 288.9767, 269.0440, 223.0600, 173.0444, 149.0235, 110.9751 | 4-Sinapoyl-5-caffeoylquinic acid | RGF |
| 64 | 29.85 | C_44_H_64_O_24_ | [M-H]- | 975.3646 | -6.46 | N/A | Crocin I | RGF |
| 65 | 30.30 | C_21_H_20_O_11_ | [M-H]- | 447.0903 | -5.59 | 447.1264, 271.0481, 243.0662, 197.0597, 169.0650, 113.0238, 85.0290 | Dihydrobaicalin | RSR |
| 66 | 30.60 | C_16_H_12_O_7_ | [M-H]- | 315.0494 | -3.49 | 315.0500, 300.0261, 242.3273, 227.2934, 153.9899, 143.3624, 126.6992, 100.8441 | Tetrahydroxymethoxy-flavone | RSR |
| 67 | 32.33 | C_21_H_18_O_11_ | [M-H]- | 445.0749 | -4.94 | 445.0744, 310.6758, 269.0440, 225.0546, 197.0597, 171.0441, 113.0238 | Glychionide A | RSR |
| 68 | 32.93 | C_16_H_12_O_7_ | [M-H]- | 315.0494 | -3.49 | 315.0497, 300.0259, 271.0237, 243.0276, 227.0337, 217.0131, 203.0319, 190.0256, 176.0103, 165.0184, 148.0157, 139.0392, 124.0157, 107.0131 | Tetrahydroxymethoxy-flavone | RSR |
| 69 | 33.01 | C_22_H_22_O_10_ | [M-H]- | 445.1104 | -6.96 | 445.1110, 430.0885, 387.3803, 269.0439, 239.0336 | Oroxylin A 7-*O*-*D*-glucuronide isomer |  |
| 70 | 34.03 | C_21_H_18_O_11_ | [M-H]- | 445.0748 | -5.17 | 445.0747, 413.7126, 383.8782, 269.0440, 241.0491, 223.0388, 197.0596, 113.0237, 85.0289 | Norwogonin-8-*O*-glucuronide | RSR |
| 71 | 34.59 | C_22_H_20_O_12_ | [M-H]- | 475.0851 | -5.47 | 417.1167, 299.0547, 284.0313, 255.0287, 181.9848, 153.9900, 85.0290 | 5,7,2 '-Trihydroxy-6-methoxy-flavone-7-*O*-glucuronide | RSR |
| 72 | 34.96 | C_22_H_22_O_11_ | [M-H]- | 461.1065 | -4.12 | 461.1066, 446.0833, 440.7041, 321.5048, 299.0546, 284.0311, 256.0367, 239.0335, 227.3018, 211.0390, 199.0386, 183.0441, 173.0596, 155.0496, 127.0546, 119.0134, | 5, 7-Dihydroxy-6-methoxy-flavanone-7-*O*-glucuronide | RSR |
| 73 | 35.36 | C_21_H_18_O_10_ | [M-H]- | 429.08 | -5.13 | 429.5549, 372.7767, 253.0491, 227.3018, 209.0593, 187.0396, 175.0235, 165.0698, 143.0494, 117.0187, 113.0237 | Chrysin-7-*O*-β-*D*-glucoronide | RSR |
| 74 | 36.74 | C_22_H_20_O_11_ | [M-H]- | 459.0904 | -5.23 | 459.0564, 345.0603, 330.0372, 315.0135, 283.0232, 269.0438, 255.0288, 239.0333, 227.2991, 211.0387, 199.0388, 183.0440, 173.0598, 155.0493, 145.0651, 127.0544, 112.9850, 85.0290 | Oroxylin A-7-*O*-glucuronide | RSR |
| 75 | 37.53 | C_38_H_54_O_19_ | [M-H]- | 813.3147 | -4.18 | 651.2652, 369.1700, 327.1586, 283.1688, 265.1583, 239.1791, 221.0652 | Crocin II | RGF |
| 76 | 38.47 | C_18_H_16_O_8_ | [M-H]- | 359.0752 | -4.18 | 359.0756, 344.0520, 329.0288, 314.0051, 295.9950, 286.0105, 268.0004, 258.0155, 242.0214, 214.0261, 194.9924, 179.9691, 169.0131, 151.9743, 133.0287, 123.9796 | 5,2',5'-Trihydroxy-6,7, 8-trimethoxy-flavonoids | RSR |
| 77 | 38.78 | C_21_H_20_O_10_ | [M-H]- | 431.0961 | -3.94 | 431.0960, 269.0440, 241.0495, 223.0386, 195.0439, 169.0647, 1356.9873, 110.0001 | Dihydroxyflavanone-*O*-glucuronide | RSR |
| 78 | 39.00 | C_21_H_20_O_10_ | [M-H]- | 431.0961 | -3.94 | 431.0960, 269.0440, 241.0495, 223.0386, 195.0439, 169.0647, 136.9873, 110.0001 | Trihydroxydihydroflavone-*O*-glucuronide | RSR |
| 79 | 39.18 | C_15_H_10_O_6_ | [M-H]- | 285.039 | -3.16 | 285.0389, 267.0287, 257.0444, 241.0498, 229.0491, 213.0545, 211.0388, 199.0390, 185.0598, 173.0595, 171.0441, 163.0027, 151.0028, 133.0287, 125.0237, 121.0287, 110.0002, 107.0132, 83.0133 | Kaempferol or isomer | RSR |
| 80 | 39.41 | C_21_H_18_O_11_ | [M-H]- | 445.0748 | -5.17 | 445.0747, 413.7126, 282.8782, 269.0440, 241.0491, 223.0388, 197.0596, 169.0650, 136.9873, 113.0237 | Baicalein-6-*O*-glucuronide isobaric conformation | RSR |
| 81 | 39.75 | C_22_H_22_O_11_ | [M-H]- | 461.106 | -5.20 | 461.1066, 446.0883, 440.7041, 321.5048, 299.0546, 284.0311, 256.0367, 239.0335, 227.3018, 211.0390, 199.0386, 183.0441, 173.0596, 155.0496, 127.0546, 119.0134, 92.5216 | Dihydroxy-methoxy-flavanone-*O*-glucuronide | RSR |
| 82 | 40.03 | C_22_H_20_O_11_ | [M-H]- | 459.0904 | -5.23 | 283.0597, 268.0362, 239.0336, 224.0474, 198.0310, 184.0520, 163.0027, 137.9949, 117.0188, 113.0238, 85.0289 | Wogonoside | RSR |
| 83 | 40.94 | C_15_H_10_O_5_ | [M-H]- | 269.0439 | -4.09 | 269.0440, 251.0339, 241.0495, 223.0390, 213.0546, 197.0597, 185.0599, 169.0648, 151.0543, 139.0028, 123.0081, 111.0081 | Norwogonin | RSR |
| 84 | 41.56 | C_23_H_22_O_12_ | [M-H]- | 489.1008 | -5.11 | 489.2679, 476.2939, 457.1331, 406.5341, 348.9805, 313.0709, 291.1938, 227.2966, 211.1330, 172.6813, 157.7558, 139.1961, 133.3512, 96.4417, 89.0238 | 5, 7-Dihydroxy-6, 8-dimethoxy-flavone-7-*O*-glucuronide | RSR |
| 85 | 42.02 | C_16_H_12_O_6_ | [M-H]- | 299.0545 | -3.68 | 299.0544, 294.1936, 284.0301, 256.0357, 277.2959, 211.0389, 199.0389, 174.0633, 165.9903, 156.0530, 133.0287, 127.0548, 110.0005 | Trihydroxymethoxy-flavone or isomer | RSR |
| 86 | 42.21 | C_27_H_36_O_11_ | [M-H]- | 535.2158 | -3.92 | 535.2164, 520.1918, 483.8649, 467.0577, 358.9395, 340.9729, 325.0912, 295.0807, 265.0703, 250.0473, 237.0754, 223.0599, 205.0493, 190.0259, 175.0025, 164.0469, 149.0235, 135.0444, 119.0313 | 6'-*O*-trans-sinapoyl jasminoside A or isomer | RGF |
| 87 | 42.21 | C_18_H_16_O_8_ | [M-H]- | 359.0753 | -3.90 | 359.0755, 344.0519, 329.0288, 314.0055, 301.0338, 284.0313, 273.0392, 258.0159, 242.0209, 227.2990, 196.0311, 180.9766, 173.0596, 154.9978, 153.9921, 133.9874, | 5,2',5'-Trihydroxy-6,7, 8-trimethoxy-flavonoids | RSR |
| 88 | 42.43 | C_17_H_14_O_7_ | [M-H]- | 329.0649 | -3.95 | 329.0651, 314.0415, 299.0181, 283.0231, 271.0236, 255.0290, 243.0283, 227.0337, 215.0341, 199.0387, 178.9976, 165.9897, 161.0238, 140.0107, 137.9950, 124.0158, 110.0003 | Trihydroxy-dimethoxy-flavoneor isomer | RSR |
| 89 | 42.99 | C_16_H_12_O_6_ | [M-H]- | 299.0542 | -4.68 | 299.0546, 284.0311, 267.0286, 256.0365, 239.0336, 228.0418, 211.0386, 200.0467, 181.9845, 171.0443, 153.9899, 151.9743, 136.9870, 125.9951, 110.0003 | Trihydroxymethoxy-flavone | RSR |
| 90 | 43.39 | C_15_H_10_O_5_ | [M-H]- | 269.0436 | -5.20 | 269.0439, 251.0336, 241.0493, 223.0388, 213.0543, 197.0596, 185.0600, 169.0649, 157.0647, 143.0497, 136.9872, 123.0081, 95.0132 | Baicalein | RSR |
| 91 | 43.44 | C_28_H_34_O_14_ | [M-H]- | 593.1844 | -4.38 | 593.0109, 575.0042, 547.0087, 509.0237, 480.0233, 452.0293, 426.0131, 401.0108, 365.9770, 340.9732, 324.9766, 295.9767, 267.0285, 239.0336, 223.0598, 207.0654, 190.0260, 164.0469, 149.0235, 136.9874, 121.0289, 101.0238 | 6'-*O*-Sinapoylgeniposide | RGF |
| 92 | 43.61 | C_17_H_14_O_7_ | [M-H]- | 329.0649 | -3.95 | 329.0652, 314.0417, 299.0182, 271.0233, 255.0285, 243.0285, 227.0337, 215.0337, 211.0389, 199.0389, 183.0440, 171.0441, 165.9897, 155.0494, 143.0494, 124.9873, 117.0339 | Trihydroxy-dimethoxy-flavone | RSR |
| 93 | 44.06 | C_16_H_12_O_6_ | [M-H]- | 299.0546 | -3.34 | 299.0549, 284.0311, 267.0287, 256.0365, 239.0341, 227.3034, 211.0388, 200.0464, 181.9848, 165.0185, 153.9899, 133.0286, 125.9953, 110.0002 | Trihydroxymethoxy-flavone or isomer | RSR |
| 94 | 44.70 | C_17_H_14_O_7_ | [M-H]- | 329.0651 | -3.34 | 329.0659, 314.0416, 299.0177, 271.0239, 227.2944, 183.9123, 152.9819, 130.6741, 58.8255 | Trihydroxy-dimethoxy-flavone or isomer | RSR |
| 95 | 45.37 | C_32_H_44_O_14_ | [M-H]- | 651.2623 | -4.61 | 651.1639, 389.0289, 337.0696, 327.1587, 295.1485, 283.1687, 265.1584, 239.1793 | all-trans-Crocetin di-β-*D*-glucosyl eater | RGF |
| 96 | 46.12 | C_44_H_64_O_24_ | [M+HCOO]- | 1021.3691 | -7.15 | N/A | Crocin I isomer | RGF |
| 97 | 46.35 | C_17_H_14_O_7_ | [M-H]- | 329.0651 | -3.34 | 329.2317, 314.0419, 311.2204, 299.0182, 293.2098, 271.0240, 229.1431, 211.1326, 201.1119, 183.1379, 171.1016, 164.9820, 157.1223, 146.9225, 139.1120, 127.1121, 121.1017, 99.0809 | Trihydroxy-dimethoxy-flavone or isomer | RSR |
| 98 | 47.39 | C_18_H_16_O_7_ | [M-H]- | 343.0802 | -4.66 | 343.0805, 328.0573, 313.0338, 298.0110, 285.0390, 273.0385, 270.0156, 253.0132, 242.0206, 226.0257, 213.0548, 198.0310, 180.0054, 164.9819, 152.0103, 136.9872, 133.0287, 120.9922, 111.0079, 96.9925, 86.47.9926 | Skullcap flavone | RSR |
| 99 | 47.49 | C_18_H_16_O_8_ | [M-H]- | 359.0752 | -4.18 | 359.0761, 344.0524, 341.1366, 329.0287, 314.0053, 311.0171, 286.0118, 258.0160, 239.1823, 227.2976, 193.1173, 133.0288 | 5,2',5'-Trihydroxy-6,7, 8-trimethoxy-flavonoids | RSR |
| 100 | 47.55 | C_27_H_36_O_11_ | [M-H]- | 535.2158 | -3.92 | 535.2535, 493.3040, 456.1633, 399.8181, 370.7679, 331.1897, 327.1585, 313.1797, 301.1795, 383.1686, 265.1584, 239.1793, 227.3026, 203.5126, 183.1170, 147.1174, 133.1015, 113.0238, 101.0238 | 6'-*O*-trans-sinapoyl jasminoside A or isomer | RGF |
| 101 | 47.59 | C_16_H_12_O_5_ | [M-H]- | 283.0593 | -4.95 | 283.0597, 268.0361, 258.4944, 239.0336, 224.0463, 211.0391, 198.0311, 184.0519, 165.9897, 163.0027, 156.0572, 148.9875, 137.9950, 110.0003 | Wogonin | RSR |
| 102 | 48.01 | C_15_H_10_O_4_ | [M-H]- | 253.049 | -4.35 | 253.0492, 247.9567, 227.2952, 209.1535, 143.0496, 115.7433, 63.0236 | Chrysin | RSR |
| 103 | 48.26 | C_17_H_14_O_6_ | [M-H]- | 313.0701 | -3.51 | 313.0703, 298.0463, 243.8532, 235.5044, 187.1684, 167.8030, 139.0975, 104.2392, 83.1999 | Dihydroxy-dimethoxy-flavone | RSR |
| 104 | 48.71 | C_19_H_18_O_8_ | [M-H]- | 373.0901 | -6.16 | 373.0910, 358.0676, 343.0440, 328.0209, 325.0344, 315.0501, 303.0497, 300.0259, 285.0024, 269.0078, 257.0078, 241.0127, 213.0181, 194.9923, 185.0232, 179.9690, 169.0133, 151.9742, 133.0288, 123.9795, 110.0004, 95.0136, 79.9899 | Skullcapflavone II | RSR |
| 105 | 48.83 | C_16_H_12_O_5_ | [M-H]- | 283.0594 | -4.59 | 283.0598, 268.0363, 239.0337, 211.0388, 195.0442, 184.0521, 163.0027, 137.9952, 123.6544, 110.0005 | Oroxylin A | RSR |
| 106 | 49.10 | C_30_H_18_O_10_ | [M-H]- | 537.0797 | -4.65 | 537.0796, 525.0049, 417.0231, 391.0439, 373.0331, 363.0488, 347.0543, 335.0549, 319.0597, 291.0645, 270.9869, 267.0285, 261.0544, 245.0077, 239.0337, 233.0594, 217.0129, 189.0181, 161.0232, 145.0286, 136.9871, 97.6789 | 8,8''-Bibaicalein | RSR |
| 107 | 49.12 | C_17_H_14_O_6_ | [M-H]- | 313.0699 | -4.15 | 313.0702, 298.0468, 283.0232, 269.0439, 255.0285, 239.0336, 227.0336, 211.0387, 199.0383, 183.0440, 167.0489, 164.9819, 152.0106, 143.0488, 136.9872, 117.0340 | Dihydroxy-dimethoxy-flavone | RSR |
| 108 | 50.47 | C_18_H_16_O_7_ | [M-H]- | 343.0802 | -4.66 | 343.0818, 328.0574, 323.1649, 313.0335, 298.0110, 270.0156, 266.2701, 227.2973, 191.4056, 151.9741, 139.0749, 116.9569 | Tenaxin I | RSR |
| 109 | 50.62 | C_48_H_60_O_22_ | [M-H]- | 987.3423 | -7.60 |  | Neocrocin B/C/D/E | RGF |
| 110 | 51.47 | C_32_H_44_O_14_ | [M-H]- | 651.2621 | -4.91 | 327.1584, 313.3103, 283.1688, 265.1583, 250.1349, 239.1792, 221.0655, | Crocin-III or isomer 1 | RGF |
| 111 | 51.69 | C_32_H_44_O_14_ | [M-H]- | 651.2621 | -4.91 | 327.1588, 283.1686, 239.1793, 179.0554, 161.0448, 139.0760, 125.0237 | Crocin-III or isomer 2 | RGF |
| 112 | 52.20 | C_32_H_44_O_14_ | [M-H]- | 651.2623 | -4.61 | 327.1586, 283.1688, 265.1587, 239.1792, 221.0654, 209.0444, 179.0554, 161.0447 | Crocin-III or isomer 3 | RGF |
| 113 | 57.03 | C_20_H_24_O_4_ | [M-H]- | 327.1584 | -3.67 | 327.1588, 309.1485, 294.1244, 283.1324, 279.1298, 266.1298, 252.0784, 229.0871, 203.0699, 190.8514, 185.0067, 146.9377, 118.2670, 102.9482 | Crocetin | RGF |

## **Table S2** Identification of compounds in PW2 by UPLC-Orbitrap HRMS

| **No** | **t_R_**  **(min)** | **Formula** | **Selection ion** | **Measured mass (m/z)** | **Error (ppm)** | **Fragmentations** | **Identification** | **Source** |
| --- | --- | --- | --- | --- | --- | --- | --- | --- |
| 1 | 1.37 | C_7_H_12_O_6_ | [M-H]- | 191.055 | -3.14 | 191.0551, 184.6895, 179.6002, 173.0081, 142.6267, 129.0187 | Quinic acid | GFP |
| 2 | 2.45 | C_16_H_22_O_11_ | [M-H]- | 389.1066 | -4.63 | 389.1064, 303.0144, 273.8625, 227.0545, 209.0443, 183.0651, 165.0547, 147.0443, 139.0392 | Deacetylasperulosidic acid | GFP |
| 3 | 3.13 | C_16_H_22_O_10_ | [M-H]- | 373.1118 | -4.56 | N/A | Gardoside | GFP |
| 4 | 3.3 | C_16_H_24_O_11_ | [M-H]- | 391.1225 | -3.84 | 391.1232, 357.5748, 229.0714, 185.0814, 156.4031, 140.1040 | Shanzhiside isomer | GFP |
| 5 | 3.66 | C_9_H_8_O_4_ | [M-H]- | 179.034 | -2.79 | 179.0340, 161.0235, 151.0394, 134.9872, 133.0287, 122.0368 | Caffeic acid | RSR |
| 6 | 3.95 | C_17_H_26_O_11_ | [M-H]- | 405.138 | -4.20 | N/A | Shanzhiside methyl ester | GFP |
| 7 | 4.16 | C_7_H_6_O_4_ | [M-H]- | 153.0185 | -1.96 | 153.0185, 109.0289 | Protocatechuic acid | RSR |
| 8 | 4.16 | C_16_H_24_O_11_ | [M-H]- | 391.1223 | -4.35 | 391.1227, 369.0520, 260.1431, 229.0706, 193.0498, 185.0810, 179.0549, 167.0705, 149.0600, 127.0394, 101.0238 | Shanzhiside | GFP |
| 9 | 4.33 | C_16_H_22_O_10_ | [M-H]- | 373.1118 | -4.56 | N/A | Geniposidic acid | GFP |
| 10 | 5.31 | C_16_H_18_O_9_ | [M-H]- | 353.0864 | -2.55 | 353.0862, 191.0553, 179.0342, 161.0238, 135.0446, | Chlorogenic acid | RSR |
| 11 | 5.37 | C_17_H_24_O_11_ | [M+HCOO]- | 449.1281 | -3.12 | 449.0826, 413.1053, 391.1225, 229.0716, 185.0813, 167.0715, 149.0603, 112.2441 | 6α-hydroxygeniposide | GFP |
| 12 | 6.91 | C_17_H_24_O_11_ | [M+HCOO]- | 449.1279 | -3.56 | 403.1235, 371.0957, 341.7132, 275.7302, 265.0701, 241.0707, 227.2978, 223.0603, 205.0495, 191.0341, 177.0549, 161.0238, 145.0288, 127.0395, 121.0290, 109.0289, 101.0239 | Gardenoside | GFP |
| 13 | 6.98 | C_16_H_24_O_10_ | [M-H]- | 375.128 | -2.93 | 339.6590, 316.9875, 276.7999, 187.2069, 177.5162, 168.3223, 137.0960, 132.1977, 113.0241, 101.0242 | Mussaenosidic acid | GFP |
| 14 | 7.22 | C_16_H_26_O_8_ | [M-H]- | 345.1536 | -3.77 | 345.1176, 315.3869 | Jasminoside B | GFP |
| 15 | 8.93 | C_17_H_24_O_11_ | [M-H]- | 403.1227 | -3.22 | 403.1594, 343.9911, 315.0065, 259.0644, 227.3047, 167.0349 | 6β-hydroxygeniposide | GFP |
| 16 | 9.37 | C_16_H_26_O_8_ | [M-H]- | 345.154 | -2.61 | 227.3028, 214.7800, 179.0552, 161.0447, 143.0914, 131.0343, 119.0344, 113.0239, 101.0239 | Picrocrocinic acid | GFP |
| 17 | 10.74 | C_16_H_18_O_9_ | [M-H]- | 353.086 | -3.68 | N/A | Neochlorogenic acid | GFP |
| 18 | 11.64 | C_16_H_18_O_9_ | [M-H]- | 353.0861 | -3.40 | N/A | Chlorogenic acid | GFP |
| 19 | 12.23 | C_7_H_6_O_4_ | [M-H]- | 153.0187 | -0.65 | 153.0186, 135.0084, 109.0290, 86.6895 | Protocatechuic acid-isomer | RSR |
| 20 | 12.42 | C_15_H_12_O_7_ | [M-H]- | 303.0493 | -3.96 | 303.0500, 285.0393, 275.0555, 259.0599, 241.0497, 217.0497, 193.0499, 177.0185, 149.0238, 125.0238, 107.0133 | 3,6,7,2',6 '-pentahydroxyflavanones | RSR |
| 21 | 12.47 | C_23_H_34_O_15_ | [M-H]- | 549.1801 | -3.28 | 595.1687, 549.1806, 517.1554, 387.1280, 225.0759, 207.0653, 191.0559, 179.0554, 123.0446, 101.0239 | Genipin 1-gentiobioside | GFP |
| 22 | 13.04 | C_17_H_22_O_10_ | [M-H]- | 385.1124 | -2.86 | 385.1134, 325.0914, 269.0710, 247.0601, 223.0602, 205.0496, 190.0263, 164.0471, 149.0238, 143.0497, 101.0239 | Sinapyglucoside | GFP |
| 23 | 13.55 | C_17_H_24_O_10_ | [M+HCOO]- | 433.1323 | -5.31 | 433.0761, 387.2013, 330.6121, 313.0702, 287.0395, 225.0759, 207.0650, 195.0292, 167.0345, 147.0444, 123.0446, 113.0240, 101.00239 | Geniposide | GFP |
| 24 | 15.22 | C_15_H_10_O_7_ | [M-H]- | 301.0338 | -3.65 | 301.0343, 283.0238, 273.0400, 257.0448, 239.0343, 215.0338, 203.0550, 193.0134, 173.0233, 155.0343, 151.0030, 125.0238, 121.0289, 107.0134 | Quercetin | RSR |
| 25 | 15.51 | C_26_H_28_O_14_ | [M-H]- | 561.1438 | -3544.94 | 563.1379, 545.1291, 503.1173, 485.1063, 473.1067, 455.0963, 443.0964, 425.0852, 413.0860, 395.0751, 383.0755, 365.0651, 353.0650, 337.0704, 325.0704, 297.0755, 283.0599, 251.0699, 221.0442, 191.0341, 161.0237, 135.0446, 117.0340 | Schaftoside | RSR |
| 26 | 15.58 | C_16_H_26_O_7_ | [M-H]- | 375.1656 | 0.27 | 285.0762, 270.0530, 255.6011, 227.2985, 180.9504, 167.1070, 161.0451, 152.0838, 125.0240, 113.0239, 101.0239 | Picrocrocin | GFP |
| 27 | 17.78 | C_27_H_30_O_16_ | [M-H]- | 609.1435 | -3.45 | 609.1435, 543.2610, 300.0261, 271.0235, 255.0288, 243.0291, 227.2986, 165.8289, 151.0031, 121.0288, 101.0245 | Rutin isomer | GFP |
| 28 | 17.84 | C_21_H_20_O_12_ | [M-H]- | 463.086 | -3.67 | 463.0864, 445.6753, 300.0263, 287.0549, 271.0236, 255.0288, 243.0288, 227.0339, 199.0288, 181.0132, 166.9976, 153.0185, 119.0496 | Carthamidin-7-*O*-glucuronide | RSR |
| 29 | 18.61 | C_27_H_30_O_16_ | [M-H]- | 609.1433 | -3.78 | 608.1436, 563.2691, 473.6065, 431.9365, 343.0438, 300.0264, 271.0237, 255.0289, 243.0289, 227.0230, 211.0394, 178.9977, 151.0030, 121.0290, 107.0134 | Rutin or isomer | GFP |
| 30 | 19.34 | C_21_H_20_O_12_ | [M-H]- | 463.0863 | -3.02 | 463.0869, 452.8129, 342.1023, 321.5673, 300.0262, 287.0554, 271.0239, 255.0289, 241.0494, 227.2932, 151.0031, 135.0444, 119.0497, 107.0134 | Isoquercetin | GFP |
| 31 | 19.39 | C_21_H_18_O_12_ | [M-H]- | 461.0706 | -3.04 | 285.0393, 139.0398, 113.0242, 102.1307 | Scutellarin | RSR |
| 32 | 19.76 | C_26_H_28_O_13_ | [M-H]- | 547.1429 | -4.20 | 547.1433, 529.1331, 449.1235, 487.1224, 457.1121, 427.1017, 409.0909, 379.0804, 367.0809, 349.0701, 337.0706, 321.0753, 309.0758, 295.0601, 281.0807, 267.0651, 252.0781, 208.0883, 191.0344, 163.0392, 145.0288, 119.0497 | Chrysin 6-*C*-arabinoside 8-*C*-glucoside | RSR |
| 33 | 20.06 | C_23_H_24_O_13_ | [M-H]- | 507.1122 | -3.35 | 492.0888, 345.0602, 330.0369, 315.0135, 297.0040, 287.0189, 269.0085, 233.0081, 201.0187, 178.9980, 164.9821, 149.0237, 136.9873, 110.0004 | Viscidulin III-2′-*O*-glucoside | RSR |
| 34 | 20.13 | C_16_H_26_O_8_ | [M-H]- | 345.1539 | -2.90 | 345.1530, 329.9427, 273.0764, 265.0503, 239.0710, 221.0598, 193.0659, 155.0346, 137.0239, 119.0344, 113.0239, 101.0240 | Jasminoside G | GFP |
| 35 | 20.53 | C_21_H_20_O_12_ | [M-H]- | 463.0861 | -3.46 | 431.0965, 2830599, 273.0756, 240.0419, 225.0547, 193.0129, 178.9978, 161.0236, 151.0029, 139.0394, 124.0160 | Carthamidin 7-*O*-glucuronide isomer | GFP |
| 36 | 21.72 | C_15_H_10_O_7_ | [M-H]- | 301.0343 | -1.66 | 301.0342, 273.0394, 256.3537, 227.3042, 217.0134, 207.3658, 177.0188, 166.9982, 151.0033, 137.0239, 125.0241, 119.0497, 111.0083, 107.0135 | 3,5,7,2',6'-Pentahydroxy flavones | GFP |
| 37 | 21.72 | C_29_H_36_O_15_ | [M-H]- | 623.1957 | -3.05 | 623.1952, 461.1646, 389.1595, 290.9883, 190.0631, 175.0394, 175.0394, 161.0238, 133.0290, 113.0238 | Verbascoside | RSR |
| 38 | 21.79 | C_23_H_24_O_12_ | [M-H]- | 491.1175 | -3.05 | 491.1577, 365.8980, 329.1023, 315.0497, 300.0261, 269.0812, 257.0805, 244.0380, 227.2989, 197.0447, 188.2922, 162.8191, 160.0524, 138.0875, 125.0242, 116.5013 | Trihydroxy-dimethoxy-flavone glucoside | RSR |
| 39 | 21.9 | C_26_H_28_O_13_ | [M-H]- | 547.1432 | -3.66 | 547.1436, 529.1332, 487.1212, 469.1111, 457.1123, 439.1026, 427.1018, 397.0923, 367.0809, 349.0701, 337.0707, 321.0752, 309.0758, 295.0598, 281.0808, 267.0650, 252.0786, 221.0440, 191.0340, 163.0393, 145.0289, 119.0498 | Chrysin 6-*C*-glucoside 8-*C*-arabinoside | RSR |
| 40 | 22.04 | C_25_H_24_O_12_ | [M-H]- | 515.1174 | -3.11 | 515.1149, 402.4895, 372.6217, 353.0864, 335.0765, 255.0654, 227.3025, 205.0500, 191.0553, 179.0342, 173.0447, 161.0237, 155.02343, 135.0446, 127.0395, 111.0447 | 3,4-Dicaffeoyl quinic acid | GFP |
| 41 | 22.29 | C_25_H_24_O_12_ | [M-H]- | 515.1177 | -2.52 | 350.9949, 323.0003, 293.0457, 279.0668, 267.0649, 253.0500, 239.4092, 194.8338, 179.0349 | 3,5-Dicaffeoyl quinic acid | GFP |
| 42 | 22.75 | C_26_H_28_O_13_ | [M-H]- | 547.1435 | -3.11 | 547.1436, 529.1329, 457.1122, 397.0913, 367.0808, 337.0707, 309.0759, 281.0809, 251.0699, 191.0342 | Chrysin 6-*C*-glucoside 8-*C*-arabinoside isomer | RSR |
| 43 | 22.87 | C_27_H_34_O_14_ | [M-H]- | 581.1852 | -3.27 | N/A | Trihydroxydihydrochalcone-3'-*C*-glucoside-6'-*O*-glucoside or isomer | RSR |
| 44 | 23.05 | C_16_H_12_O_7_ | [M-H]- | 315.0501 | -1.27 | 315.0507, 300.0262, 270.6669, 253.0501, 242.0578, 227.2994, 209.0600, 199.0763, 165.9901, 145.0292, 110.0006 | Tetrahydroxymethoxy-flavone | RSR |
| 45 | 23.46 | C_29_H_36_O_15_ | [M-H]- | 623.1956 | -3.21 | 623.1592, 461.1070, 447.1180, 432.0951, 299.0550, 283.0237, 268.0343, 239.134, 226.0266, 211.0391, 199.0390, 183.0443, 173.0600, 155.0496, 127.0548 | Isoacteoside | RSR |
| 46 | 23.51 | C_21_H_20_O_9_ | [M-H]- | 415.1017 | -2.89 | 415.1015, 397.0900, 325.0703, 307.0598, 295.0596, 267.0648, 253.0493, 223.0751, 181.0646, 145.0287, 121.0287 | Chrysin 8-*C*-glucoside | RSR |
| 47 | 23.56 | C_21_H_20_O_9_ | [M-H]- | 415.1015 | -3.37 | 415.1024, 375.5512, 337.0710, 325.0708, 295.0601, 267.0653, 253.0497, 223.0757, 181.0653, 145.0289, 121.0289, 77.0393 | Chrysin 6-*C*-glucoside | RSR |
| 48 | 23.76 | C_21_H_20_O_10_ | [M-H]- | 431.0966 | -2.78 | 431.0969, 388.2481, 340.3229, 269.0446, 251.0345, 218.9976, 191.0015, 165.9904, 139.0395, 124.0163, 107.0499 | Apigenin 7-glucoside | RSR |
| 49 | 24.32 | C_22_H_22_O_10_ | [M-H]- | 445.1121 | -3.15 | 445.1125, 430.0888, 341.0676, 314.2318, 283.0602, 269.0446, 251.0340, 239.0342, 223.0395, 195.0444, 169.0651, 136.9875, 117.0342, 85.0292 | Oroxylin A 7-*O*-*D*-glucuronide | RSR |
| 50 | 24.45 | C_16_H_12_O_7_ | [M-H]- | 315.05 | -1.59 | 315.0499, 300.0258, 227.2949, 206.9404, 181.7298, 151.0033, 140.0341, 107.0137 | Tetrahydroxymethoxy flavone | RSR |
| 51 | 25.49 | C_17_H_14_O_8_ | [M-H]- | 345.0598 | -3.77 | 345.0609, 330.0373, 315.0136, 282.1828, 270.8318, 270.8318, 252.2360, 206.8936, 178.9983, 164.9820, 149.0240, 138.9798, 123.3110, 109.2251 | Viscidulin III | RSR |
| 52 | 25.99 | C_22_H_36_O_12_ | [M-H]- | 537.2168 | -2.79 | 573.1964, 368.1045, 278.3393, 227.3046, 205.0501, 190.0269, 175.0029, 167.0343, 152.0108, 126.2366, 123.0448, 108.0213, 103.1463 | Jasminoside Q | GFP |
| 53 | 26.06 | C_15_H_10_O_6_ | [M-H]- | 285.0393 | -2.10 | 285.0392, 151.0034, 143.4746, 107.0135, 83.1931 | Kaempferol or isomer | RSR |
| 54 | 26.07 | C_22_H_20_O_12_ | [M-H]- | 475.0859 | -3.79 | 475.1633, 377.5968, 299.0551, 284.0325, 237.3882, 227.3051, 216.9124, 185.7218, 137.0237, 109.3668, 96.9598 | Trihydroxy-methoxy-flavone-7-*O*-glucuronide | RSR |
| 55 | 26.33 | C_16_H_12_O_7_ | [M-H]- | 315.05 | -1.59 | 315.0500, 308.6601, 272.0317, 227.0341, 176.8687, 165.0182, 149.0235, 139.0394, 114.0633 | Tetrahydroxymethoxy-flavone | RSR |
| 56 | 26.62 | C_32_H_40_O_17_ | [M-H]- | 695.2159 | -4.03 | 695.2153, 619.1983, 469.1328, 451.1227, 409.1113, 367.1018, 307.0812, 269.0457, 265.0704, 225.0759, 207.0654, 163.0393, 145.0288, 123.0446, 101.0239 | 6''-*O*-[(*E*)-*p*-Coumaroyl] genipin gentiobioside or isomer | GFP |
| 57 | 26.68 | C_31_H_32_O_16_ | [M-H]- | 659.1595 | -2.58 | 659.1925, 644.1687, 613.0625, 513.1347, 497.1260, 435.1282, 407.0064, 353.0886, 335.0765, 273.0977, 253.0490, 233.0658, 201.0162, 191.0553, 173.0447, 151.0448, 155.0343, 135.0446, | 3,5-Di-*O*-Caffeoyl-4-*O*-(3-hydroxy-3-methyl) glutaroylquinic acid | GFP |
| 58 | 27.21 | C_27_H_28_O_13_ | [M-H]- | 559.1439 | -2.32 | 559.1429, 541.1338, 485.1081, 455.0961, 421.0930, 365.0653, 335.0552, 295.0604, 267.0652, 258.1502, 227.2990, 189.0184, 135.0450, 113.0240 | 3-*O*-Sinapoyl-5-*O*-caffeoylquinic acid | GFP |
| 59 | 27.52 | C_34_H_44_O_19_ | [M-H]- | 755.2367 | -4.24 | 755.3920, 694.2052, 596.4949, 453.5662, 432.0727, 323.0674, 259.2663, 227.3000, 205.0499, 190.0268, 164.0476, 140.9125, 123.0451, 101.0241 | 6''-*O*-[trans-Sinapoyl] genipin gentiobioside | GFP |
| 60 | 27.81 | C33H42O18 | [M-H]- | 725.2266 | -3.72 | 725.1813, 701.4175, 588.7101, 411.0707, 359.1507, 338.0733, 227.3055, 193.6127, 127.6206, | 6''-*O*-[trans-Feruloyl] genipin gentiobioside | GFP |
| 61 | 27.98 | C_27_H_36_O_12_ | [M-H]- | 551.2121 | -1.45 | 551.2151.536.1866, 467.7326, 352.9226, 325.0919, 311.0756, 295.0815, 281.0665, 265.0709, 250.0474, 237.0760, 223.0604, 205.0499, 190.0264, 175.0030, 164.0473, 149.0238, 135.0447, 121.0292 | 6'-*O*-trans-Sinapoyljasminoside L | GFP |
| 62 | 28.79 | C_21_H_18_O_11_ | [M-H]- | 445.0757 | -3.15 | 445.1129, 283.0612, 269.0448, 239.0343, 195.0445, 169.0654, 111.1227, 85.0292 | Baicalin | RSR |
| 63 | 29.06 | C_27_H_28_O_13_ | [M-H]- | 559.1437 | -2.68 | 559.0306, 487.0490, 439.1199, 382.9977, 353.0243, 288.9776, 269.0446, 227.2980, 211.5595, 149.4516, 110.9753 | 4-Sinapoyl-5-caffeoylquinic acid | GFP |
| 64 | 29.82 | C_44_H_64_O_24_ | [M-H]- | 975.3674 | -3.59 | N/A | Crocin I | GFP |
| 65 | 30.37 | C_21_H_20_O_11_ | [M-H]- | 447.0914 | -3.13 | 447.0800, 361.4261, 271.0497, 243.0540, 225.0553, 197.0599, 171.0443, 113.0238 | Dihydrobaicalin | RSR |
| 66 | 30.6 | C_16_H_12_O_7_ | [M-H]- | 315.05 | -1.59 | 315.0500, 300.0257, 227.2978, 165.9900, 145.8828, 138.7487 | Tetrahydroxymethoxy-flavone | RSR |
| 67 | 32.34 | C_21_H_18_O_11_ | [M-H]- | 445.0755 | -3.59 | 445.0765, 349.1569, 269.0444, 225.0548, 197.0600, 171.0444, 113.0240 | Glychionide A | RSR |
| 68 | 33.04 | C_16_H_12_O_7_ | [M-H]- | 315.0502 | -0.95 | 315.0505, 300.0267, 271.0237, 243.0288, 148.0160, 139.0397, 107.0135 | Tetrahydroxymethoxy-flavone | RSR |
| 69 | 33.01 | C_22_H_22_O_10_ | [M-H]- | 445.1115 | -4.49 | 445.1128, 430.0890, 390.1039, 349.5295, 269.0445, 251.0343, 239.0341, 223.0390, 197.0602 | Oroxylin A 7-*O*-*D*-glucuronide isomer |  |
| 70 | 34.06 | C_21_H_18_O_11_ | [M-H]- | 445.0755 | -3.59 | 445.0758, 404.7560, 370.4137, 269.0444, 241.0496, 223.0391, 197.0599, 136.9872, 113.0239 | Norwogonin-8-*O*-glucuronide | RSR |
| 71 | 34.66 | C_22_H_20_O_12_ | [M-H]- | 475.0861 | -3.37 | 423.1509, 299.0551, 284.0315, 239.0344, 181.9850, 153.9902, 113.0240, 95.0291 | 5,7,2'-Trihydroxy-6-methoxy-flavone-7-*O*-glucuronide | RSR |
| 72 | 34.98 | C_22_H_22_O_11_ | [M-H]- | 461.1073 | -2.39 | 461.1077, 446.0862, 299.0549, 284.0317, 239.0343, 227.2989, 211.0392, 183.0441, 173.0602, 155.0497, 127.0550 | 5, 7-Dihydroxy-6-methoxy-flavanone-7-*O*-glucuronide | RSR |
| 73 | 35.37 | C_21_H_18_O_10_ | [M-H]- | 429.0807 | -3.50 | N/A | Chrysin-7-*O*-β-*D*-glucoronide | RSR |
| 74 | 36.79 | C_22_H_20_O_11_ | [M-H]- | 459.0914 | -3.05 | 283.0602, 268.0367 | Oroxylin A-7-*O*-glucuronide | RSR |
| 75 | 37.42 | C_38_H_54_O_19_ | [M-H]- | 859.3212 | -2.79 | 691.6539, 283.1698, 227.3001, 135.3897, 101.0241 | Crocin II | GFP |
| 76 | 38.55 | C_18_H_16_O_8_ | [M-H]- | 359.0758 | -2.51 | 359.0762, 344.0525, 329.0293, 314.0058, 286.0110, 258.0162, 201.0187, 194.9927, 179.9693, 151.9745, 133.0290, 123.9797 | 5,2 ',5 '-Trihydroxy-6,7, 8-trimethoxy-flavonoids | RSR |
| 77 | 38.77 | C_21_H_20_O_10_ | [M-H]- | 431.0969 | -2.09 | 431.0969, 292.3767, 269.0445, 227.3013, 181.0682, 134.1198 | Dihydroxyflavanone-*O*-glucuronide | RSR |
| 78 | 38.84 | C_21_H_20_O_10_ | [M-H]- | 431.0969 | -2.09 | 431.0967, 269.0445, 255.0553, 223.0392, 165.9900, 137.9955, 110.0005 | Trihydroxydihydroflavone-*O*-glucuronide | RSR |
| 79 | 39.35 | C_15_H_10_O_6_ | [M-H]- | 285.0393 | -2.10 | 285.0394, 267.0292, 229.0498, 213.0548, 199.0393, 185.0600, 171.0444, 151.0030, 133.0289, 125.0239, 107.0134, 83.0135 | Kaempferol or isomer | RSR |
| 80 | 39.47 | C_21_H_18_O_11_ | [M-H]- | 445.0754 | -3.82 | 445.0758, 370.4037, 269.0444, 241.0496, 223.0391, 197.0599, 136.9872, 113.0239 | Baicalein-6-*O*-glucuronide isobaric conformation | RSR |
| 81 | 39.75 | C_22_H_22_O_11_ | [M-H]- | 461.1067 | -3.69 | 461.1067, 446.0838, 299.0550, 284.0316, 268.0367, 239.0342, 211.0392, 183.0443, 173.0600, 155.0496, 127.0549 | Dihydroxy-methoxy-flavanone-*O*-glucuronide | RSR |
| 82 | 39.98 | C_22_H_20_O_11_ | [M-H]- | 459.0911 | -3.70 | 283.0602, 268.0367, 239.0338, 184.0522, 165.9900 | Wogonoside | RSR |
| 83 | 40.96 | C_15_H_10_O_5_ | [M-H]- | 269.0443 | -2.60 | 269.0445, 241.0496, 223.0391, 197.0598, 169.0652 | Norwogonin | RSR |
| 84 | 41.55 | C_23_H_22_O_12_ | [M-H]- | 489.1015 | -3.68 | N/A | 5, 7-Dihydroxy-6, 8-dimethoxy-flavone-7-*O*-glucuronide | RSR |
| 85 | 41.91 | C_16_H_12_O_6_ | [M-H]- | 299.0552 | -1.34 | 299.0558, 284.0315, 278.0808, 231.8694, 227.3009, 216.8353, 179.3428, 153.9905 | Trihydroxymethoxy-flavone or isomer | RSR |
| 86 | 42.14 | C_27_H_36_O_11_ | [M-H]- | 535.2169 | -1.87 | 535.2172, 520.1932, 467.0577, 436.2687, 355.0095, 315.0475, 269.0445, 223.0602, 205.0498, 190.0265, 175.0031, 164.0473 | 6'-*O*-trans-sinapoyl jasminoside A or isomer | GFP |
| 87 | 42.19 | C_18_H_16_O_8_ | [M-H]- | 359.0763 | -1.11 | 359.0761, 344.0526, 329.0294, 314.0057, 299.0551, 284.0317, 273.0396, 242.0214, 198.0314, 180.9773, 152.9824, 124.9876 | 5,2',5'-Trihydroxy-6,7, 8-trimethoxy-flavonoids | RSR |
| 88 | 42.48 | C_17_H_14_O_7_ | [M-H]- | 329.0655 | -2.13 | 329.0659, 314.0423, 299.0118, 285.0393, 271.0240, 225.0293, 227.0342, 215.0342, 183.0445, 155.0497, 143.0497, 124.9876 | Trihydroxy-dimethoxy-flavoneor isomer | RSR |
| 89 | 43.04 | C_16_H_12_O_6_ | [M-H]- | 299.0548 | -2.68 | 299.0552, 284.0317, 267.0291, 256.0368, 239.0342, 211.0396, 181.9849, 153.9902, 125.9954 | Trihydroxymethoxy-flavone | RSR |
| 90 | 43.44 | C_15_H_10_O_5_ | [M-H]- | 269.0443 | -2.60 | 269.0445, 241.0497, 223.0391, 197.0601, 169.0652, 136.9875, 123.0082 | Baicalein | RSR |
| 91 | 43.44 | C_28_H_34_O_14_ | [M-H]- | 593.1857 | -2.19 | 593.0122, 547.0105, 509.0259, 466.0299, 413.1233, 364.9729, 340.9744, 324.9778, 239.0342, 223.0604, 207.0655, 164.0478, 149.0238, 101.0240 | 6'-*O*-Sinapoylgeniposide | GFP |
| 92 | 43.61 | C_17_H_14_O_7_ | [M-H]- | 329.0656 | -1.82 | 329.0653, 314.0432, 299.0188, 285.0395, 271.0240, 255.0288, 243.0288, 227.0341, 215.0345, 183.0444, 155.0496, 143.0496, 117.0341 | Trihydroxy-dimethoxy-flavone | RSR |
| 93 | 44.15 | C_16_H_12_O_6_ | [M-H]- | 299.055 | -2.01 | 299.0559, 284.0317, 267.0287, 255.0295, 239.0342, 200.0474, 181.9851, 153.9903, 125.9956 | Trihydroxymethoxy-flavone or isomer | RSR |
| 94 | 44.7 | C_17_H_14_O_7_ | [M-H]- | 329.0658 | -1.22 | 329.0655, 314.0427, 299.0197, 286.9426, 271.0247, 246.2342, 227.3006, 199.0394, 170.6515, 138.0515, 103.8637 | Trihydroxy-dimethoxy-flavone or isomer | RSR |
| 95 | 45.33 | C_32_H_44_O_14_ | [M-H]- | 651.2634 | -2.92 | 651.1715, 427.1001, 369.1708, 327.1590, 283.1694, 239.1798, 221.0660, 161.0451, 101.0240 | all-trans-Crocetin di-β-*D*-glucosyl eater | GFP |
| 96 | 46.07 | C_44_H_64_O_24_ | [M+HCOO]- | 1021.372 | 4.31 | N/A | Crocin I isomer | GFP |
| 97 | 46.29 | C_17_H_14_O_7_ | [M-H]- | 329.0656 | -1.82 | 329.0656, 314.0423, 299.0187, 285.0394, 271.0244, 229.1444, 199.0389, 171.0449, 164.9823, 136.9878, 119.5951 | Trihydroxy-dimethoxy-flavone or isomer | RSR |
| 98 | 47.41 | C_18_H_16_O_7_ | [M-H]- | 343.0806 | -3.50 | 343.1538, 327.1223, 311.1280, 297.1126, 227.3020, 194.5568, 164.9824 | Skullcap flavone | RSR |
| 99 | 47.47 | C_18_H_16_O_8_ | [M-H]- | 359.0757 | -2.78 | 359.1489, 344.0526, 329.0293, 314.0059, 286.0111, 258.0162, 229.0139, 202.0267, 151.9748, 133.0288, 100.9335 | 5,2',5'-Trihydroxy-6,7, 8-trimethoxy-flavonoids | RSR |
| 100 | 47.53 | C_27_H_36_O_11_ | [M-H]- | 535.2167 | -2.24 | 535.2510, 351.1936, 331.1904, 301.1796, 239.1796, 227.2996 | 6'-*O*-trans-sinapoyl jasminoside A or isomer | GFP |
| 101 | 47.64 | C_16_H_12_O_5_ | [M-H]- | 283.0598 | -3.18 | 283.0603, 268.0367, 250.1212, 239.0340, 198.0314, 165.9901, 163.0030, 155.0493, 139.0549, 110.0005 | Wogonin | RSR |
| 102 | 48.03 | C_15_H_10_O_4_ | [M-H]- | 253.0496 | -1.98 | 253.0496, 227.2961, 180.4176, 167.0399, 143.0500, 107.0135 | Chrysin | RSR |
| 103 | 48.27 | C_17_H_14_O_6_ | [M-H]- | 313.0706 | -1.92 | 313.0713, 298.0480, 271.3533, 267.9435, 227.2980, 221.2480, 117.8929, 106.8798, 89.5234 | Dihydroxy-dimethoxy-flavone | RSR |
| 104 | 48.71 | C_19_H_18_O_8_ | [M-H]- | 373.0909 | -4.02 | 373.0915, 359.0681, 343.0446, 328.0213, 300.0265, 285.0031, 257.0082, 213.0184, 194.9927, 151.9745, 133.0291 | Skullcapflavone II | RSR |
| 105 | 48.8 | C_16_H_12_O_5_ | [M-H]- | 283.06 | -2.47 | 283.0601, 268.0367, 255.0280, 239.0340, 211.0394, 184.0519, 163.0030, 137.9952, 110.0004 | Oroxylin A | RSR |
| 106 | 49.1 | C_30_H_18_O_10_ | [M-H]- | 537.0805 | -3.17 | 537.0806, 391.0446, 373.0339, 335.0551, 270.9874, 345.0081, 214.0134, 145.0289, 133.0288 | 8,8''-Bibaicalein | RSR |
| 107 | 49.12 | C_17_H_14_O_6_ | [M-H]- | 313.0705 | -2.24 | 313.0708, 298.0473, 283.0239, 269.0453, 255.0293, 239.0336, 227.0338, 201.1277, 183.0446, 164.9822, 136.9875, 121.0291 | Dihydroxy-dimethoxy-flavone | RSR |
| 108 | 50.44 | C_18_H_16_O_7_ | [M-H]- | 343.0807 | -3.21 | 343.0812, 328.0580, 313.0343, 285.0393, 270.0167, 227.2948, 180.0062, 164.9822, 136.9875, 121.9009 | Tenaxin I | RSR |
| 109 | 50.54 | C_48_H_60_O_22_ | [M-H]- | 987.3458 | -4.05 | N/A | Neocrocin B/C/D/E | GFP |
| 110 | 51.47 | C_32_H_44_O_14_ | [M-H]- | 651.2633 | -3.07 | N/A | Crocin-III or isomer 1 | GFP |
| 111 | 51.67 | C_32_H_44_O_14_ | [M-H]- | 651.2634 | -2.92 | N/A | Crocin-III or isomer 2 | GFP |
| 112 | 52.21 | C_32_H_44_O_14_ | [M-H]- | 651.2634 | -2.92 | N/A | Crocin-III or isomer 3 | GFP |
| 113 | 56.95 | C_20_H_24_O_4_ | [M-H]- | 327.1592 | -1.22 | 327.1832, 262.1368, 227.2971, 185.0074 | Crocetin | GFP |

## **Table S3** Identification of compounds in PW3 by UPLC-Orbitrap HRMS

| **No** | **t_R_**  **(min)** | **Formula** | **Selection ion** | **Measured mass (m/z)** | **error (ppm)** | **Fragmentations** | **Identification** | **Source** |
| --- | --- | --- | --- | --- | --- | --- | --- | --- |
| 1 | 1.37 | C_7_H_12_O_6_ | [M-H]- | 191.055 | -3.14 | 191.0551, 184.6895, 179.6002, 173.0081, 142.6267, 129.0187 | Quinic acid | RGF |
| 2 | 2.45 | C_16_H_22_O_11_ | [M-H]- | 389.1066 | -4.63 | 389.1064, 303.0144, 273.8625, 227.0545, 209.0443, 183.0651, 165.0547, 147.0443, 139.0392 | Deacetylasperulosidic acid | RGF |
| 3 | 3.13 | C_16_H_22_O_10_ | [M-H]- | 373.1118 | -4.56 | N/A | Gardoside | RGF |
| 4 | 3.3 | C_16_H_24_O_11_ | [M-H]- | 391.1225 | -3.84 | 391.1232, 357.5748, 229.0714, 185.0814, 156.4031, 140.1040 | Shanzhiside isomer | WSR |
| 5 | 3.66 | C_9_H_8_O_4_ | [M-H]- | 179.034 | -2.79 | 179.0340, 161.0235, 151.0394, 134.9872, 133.0287, 122.0368 | Caffeic acid | RGF |
| 6 | 3.95 | C_17_H_26_O_11_ | [M-H]- | 405.138 | -4.20 | N/A | Shanzhiside methyl ester | WSR |
| 7 | 4.16 | C_7_H_6_O_4_ | [M-H]- | 153.0185 | -1.96 | 153.0185, 109.0289 | Protocatechuic acid | RGF |
| 8 | 4.16 | C_16_H_24_O_11_ | [M-H]- | 391.1223 | -4.35 | 391.1227, 369.0520, 260.1431, 229.0706, 193.0498, 185.0810, 179.0549, 167.0705, 149.0600, 127.0394, 101.0238 | Shanzhiside | RGF |
| 9 | 4.33 | C_16_H_22_O_10_ | [M-H]- | 373.1118 | -4.56 | N/A | Geniposidic acid | WSR |
| 10 | 5.34 | C_16_H_18_O_9_ | [M-H]- | 353.0865 | -2.27 | 325.3858, 292.2612, 227.2956, 191.0553, 179.0346, 135.0445 | Chlorogenic acid | RGF |
| 11 | 5.44 | C_17_H_24_O_11_ | [M+HCOO]- | 449.1281 | -3.12 | 449.1187, 340.9545, 323.3509, 245.5356, 241.0715, 227.2967, 191.0332, 160.5686, 137.6511, 101.0243 | 6α-hydroxygeniposide | RGF |
| 12 | 6.87 | C_17_H_24_O_11_ | [M+HCOO]- | 449.1279 | -3.56 | 449.1187, 340.9545, 323.3509, 245.5356, 241.0715, 227.2967, 191.0332, 160.5686, 137.8611, 101.0243 | Gardenoside | RGF |
| 13 | 7.1 | C_16_H_24_O_10_ | [M-H]- | 375.1281 | -2.67 | 375.1256, 216.3918, 213.0760, 187.5630, 169.0864, 151.0758, 125.0603, 113.0240, 107.0497, 101.0497 | Mussaenosidic acid | RGF |
| 14 | 7.28 | C_16_H_26_O_8_ | [M-H]- | 345.1537 | -3.48 | 345.1176, 315.3869 | Jasminoside B | RGF |
| 15 | 8.95 | C_17_H_24_O_11_ | [M-H]- | 403.123 | -2.48 | 403.1599, 227.2927, 191.0555, 149.0240, 137.0238, 59.0136 | 6β-hydroxygeniposide | RGF |
| 16 | 9.49 | C_16_H_26_O_8_ | [M-H]- | 345.154 | -2.61 | 179.0552, 161.0449, 153.0915, 149.0452, 119.0345, 113.0239, 101.0239 | Picrocrocinic acid | RGF |
| 17 | 10.69 | C_16_H_18_O_9_ | [M-H]- | 353.0861 | -3.40 | N/A | Neochlorogenic acid | RGF |
| 18 | 11.69 | C_16_H_18_O_9_ | [M-H]- | 353.086 | -3.68 | N/A | Chlorogenic acid | WSR |
| 19 | 12.17 | C_7_H_6_O_4_ | [M-H]- | 153.0188 | 0.00 | 153.0187, 145.7366, 138.0968, 135.0082, 109.0290, 81.0342 | Protocatechuic acid-isomer | WSR |
| 20 | 12.36 | C_15_H_12_O_7_ | [M-H]- | 303.0495 | -3.30 | 303.0500, 285.0401, 275.0555, 259.0593, 241.0499, 227.2981, 217.0498, 177.0186, 175.0392, 149.0238, 133.0287, 125.0239, 123.0447, 107.0135 | 3,6,7,2',6'-pentahydroxyflavanones | RGF |
| 21 | 12.48 | C_23_H_34_O_15_ | [M-H]- | 549.1804 | -2.73 | 517.1541, 387.1277, 337.0935, 225.0758, 207.0651, 179.0551, 147.0444, 123.0445, 101.0238 | Genipin 1-gentiobioside | RGF |
| 22 | 13.08 | C_17_H_22_O_10_ | [M-H]- | 385.1125 | -2.60 | 247.0602, 223.0602, 206.0575, 190.0264, 179.0706, 164.0471, 149.0237 | Sinapyglucoside | RGF |
| 23 | 13.49 | C_17_H_24_O_10_ | [M+HCOO]- | 433.1329 | -3.92 | 387.2027, 313.1498, 301.0351, 225.0759, 207.0656, 195.0289, 147.0445, 123.0447, 119.0348, 101.0240 | Geniposide | WSR |
| 24 | 15.22 | C_15_H_10_O_7_ | [M-H]- | 301.0339 | -3.32 | 301.0343, 283.0242, 253.2238, 227.2967, 193.0133, 161.0238, 151.0031, 139.0396, 125.0239, 121.0290, 107.0134 | Quercetin | WSR |
| 25 | 15.56 | C_26_H_28_O_14_ | [M-H]- | 563.1385 | -2.84 | 563.1384, 545.1293, 527.1172, 503.1184, 485.1072, 473.1071, 455.0969, 443.0970, 425.0863, 413.0868, 395.0757, 383.0760, 365.0654, 353.0654, 335.0549, 325.0707, 283.0602, 267.0648, 251.0704, 221.0454, 191.0343, 161.0237, 135.0447, 117.0341 | Schaftoside | RGF |
| 26 | 15.57 | C_16_H_26_O_7_ | [M-H]- | 375.1647 | -2.13 | 285.0743, 271.5463, 227.2925, 167.1071, 161.0450, 152.0840, 149.0965, 125.0239, 113.0238, 101.0240 | Picrocrocin | RGF |
| 27 | 17.79 | C_27_H_30_O_16_ | [M-H]- | 609.1435 | -3.45 | 609.1433, 433.1498, 418.1244, 373.1277, 343.0457, 300.0266, 271.0239, 255.0289, 243.0290, 227.0348, 199.0396, 178.9982, 151.0032, 138.0328, 107.0135 | Rutin or isomer | WSR |
| 28 | 17.91 | C_21_H_20_O_12_ | [M-H]- | 463.086 | -3.67 | 463.0866, 419.0879, 300.0266, 287.0552, 271.0239, 255.0289, 243.0293, 227.0338, 201.0548, 181.0135, 166.9980, 153.0188, 148.0161, 139.0031, 133.0140, 123.0082, 119.0498, 113.0240107.0134 | Carthamidin-7-Oglucuronide | RGF |
| 29 | 18.56 | C_27_H_30_O_16_ | [M-H]- | 609.1437 | -3.12 | 608.1438, 591.1337, 522.9261, 411.3524, 343.0444, 300.0264, 271.0238, 255.0259, 243.0290, 227.0340, 199.0392, 178.9978, 151.0031, 121.0290, 107.0134 | Rutin or isomer | RGF |
| 30 | 19.3 | C_21_H_20_O_12_ | [M-H]- | 463.0864 | -2.81 | 463.0867, 300.0266, 287.0446, 271.0238, 255.0291, 243.0291, 227.0343, 211.0393, 178.9981, 163.0032, 151.0031, 148.0160, 113.0239 | Isoquercetin | WSR |
| 31 | 19.57 | C_21_H_18_O_12_ | [M-H]- | 461.0705 | -3.25 | 285.2394, 267.0285, 239.0338, 211.0397, 139.0034, 113.0238 | Scutellarin | WSR |
| 32 | 19.75 | C_26_H_28_O_13_ | [M-H]- | 547.1433 | -3.47 | 547.1439, 529.1324, 517.1333, 487.1232, 427.1126, 427.1020, 409.0919, 379.0814, 367.0810, 363.0871, 349.0705, 337.0708, 321.0757, 309.0758, 295.0603, 281.0810, 267.0652, 252.0787, 227.2931, 203.0341, 163.0393, 145.0290, 119.0497 | Chrysin 6-*C*-arabinoside 8-C-glucoside | WSR |
| 33 | 20.06 | C_23_H_24_O_13_ | [M-H]- | 507.1124 | -2.96 | 507.1129, 492.0889, 470.0897, 345.0604, 330.0371, 315.0137, 287.0186, 269.0082, 233.0081, 201.0188, 178.9976, 164.9822, 149.0238, 139.0032, 110.0005 | viscidulin III-2′-*O*-glucoside | RGF |
| 34 | 20.16 | C_16_H_26_O_8_ | [M-H]- | 345.1542 | -2.03 | 345.8903, 330.0378, 327.0140, 315.0128, 287.0200, 267.1412, 193.0138, 181.7370, 149.0241, 146.2420, 121.0293, 113.0240, 101.0242 | jasminoside *G* | RGF |
| 35 | 20.53 | C_21_H_20_O_12_ | [M-H]- | 463.0863 | -3.02 | 463.0827, 300.0269, 287.0445, 271.0238, 239.0345, 213.0560, 185.0597, 166.9976, 139.0031, 113.0240 | carthamidin 7-*O*-glucuronide isomer | RGF |
| 36 | 21.66 | C_15_H_10_O_7_ | [M-H]- | 301.0344 | -1.33 | 301.0344, 255.0294, 239.4329, 227.2990, 192.1110, 178.9978, 165.0184, 151.0030, 133.0288, 125.0239, 121.0289, 109.0291, 107.0137 | 3,5,7,2',6'-pentahydroxy flavones | WSR |
| 37 | 21.78 | C_29_H_36_O_15_ | [M-H]- | 623.1954 | -3.53 | 623.1957, 524.7410, 461.1648, 431.3509, 387.9999, 260.2113, 227.2980, 179.0344, 161.0237, 148.0789, 133.0289, 113.0240 | Verbascoside | WSR |
| 38 | 21.78 | C_23_H_24_O_12_ | [M-H]- | 491.1177 | -2.65 | 491.1574, 329.1022, 315.0504, 300.0262, 269.0814, 257.0811, 239.0717, 197.0453, 175.0756, 160.0527, 137.0239, 125.0242 | Trihydroxy-dimethoxy-flavone glucoside | WSR |
| 39 | 21.78 | C_26_H_28_O_13_ | [M-H]- | 547.143 | -4.02 | 547.1438, 529.1325, 487.1234, 469.1132, 457.1124, 439.1027, 427.1019, 421.0930, 397.0907, 379.0815, 367.0810, 349.0703, 337.0708, 321.0756, 309.0760, 295.0601, 281.0809, 267.0655, 252.0783, 237.0917, 203.0345, 163.0395, 145.0291, 119.0497 | Chrysin 6-*C*-glucoside 8-*C*-arabinoside | WSR |
| 40 | 22.03 | C_25_H_24_O_12_ | [M-H]- | 515.1176 | -2.72 | 353.0867, 335.0768, 264.5918, 249.6067, 227.3035, 205.0501, 191.0553, 179.1343, 173.0448, 161.0238, 155.0347, 135.0446 | 3,4-Dicaffeoyl quinic acid | RGF |
| 41 | 22.26 | C_25_H_24_O_12_ | [M-H]- | 515.1176 | -2.72 | 353.0866, 3335.0744, 301.2288, 227.3066, 205.0490, 197.0553, 179.0342, 173.0447, 161.0237, 155.0342, 135.0446, 127.0396, 111.0448 | 3,5-Dicaffeoyl quinic acid | WSR |
| 42 | 22.76 | C_26_H_28_O_13_ | [M-H]- | 547.1433 | -3.47 | 547.1437, 457.1124, 427.1019, 297.0916, 379.0812, 367.0811, 337.0707, 309.0758, 281.0810 | Chrysin 6-*C*-glucoside 8-*C*-arabinoside isomer | WSR |
| 43 | 22.86 | C_27_H_34_O_14_ | [M-H]- | 581.1852 | -3.27 | N/A | trihydroxydihydrochalcone-3'-*C*-glucoside-6'-Oglucoside or isomer | WSR |
| 44 | 22.98 | C_16_H_12_O_7_ | [M-H]- | 315.0499 | -1.90 | 315.0500, 300.0265, 283.0241, 255.0293, 227.0338, 201.0184, 165.9900, 139.0031, 110.0005 | Tetrahydroxymethoxy-flavone | WSR |
| 45 | 23.46 | C_29_H_36_O_15_ | [M-H]- | 623.1951 | -4.01 | 623.1959, 461.4646, 389.1594, 345.1085, 569.0444, 227.3035, 203.2363, 179.0345, 161.0237, 133.0290, 113.0239 | Isoacteoside | WSR |
| 46 | 23.58 | C_21_H_20_O_9_ | [M-H]- | 415.1013 | -3.85 | 415.1021, 337.0705, 325.0707, 307.0605, 295.0600, 279.0651, 267.0652, 253.0496, 223.0755, 195.0808, 188.0651, 165.0186, 145.0288, 121.0289, 105.0341 | Chrysin 8-*C*-glucoside | WSR |
| 47 | 23.58 | C_21_H_20_O_9_ | [M-H]- | 415.1013 | -3.85 | 415.1021, 337.0705, 325.0707, 295.0600, 267.0652, 253.0496, 223.0755, 181.0651, 145.0289, 121.0289, 77.0393 | Chrysin 6-*C*-glucoside | WSR |
| 48 | 23.76 | C_21_H_20_O_10_ | [M-H]- | 431.0966 | -2.78 | 431.0968, 385.0238, 269.0445, 241.0499, 223.0391, 211.0394, 165.9901, 139.0395, 124.0161, 110.0005 | Apigenin 7-glucoside | WSR |
| 49 | 24.32 | C_22_H_22_O_10_ | [M-H]- | 445.1123 | -2.70 | 445.1131, 430.0880, 368.1545, 341.0662, 300.4005, 283.0602, 269.0445, 239.0340, 169.0654 | Oroxylin A 7-*O*-*D*-glucuronide | WSR |
| 50 | 24.45 | C_16_H_12_O_7_ | [M-H]- | 315.05 | -1.59 | 315.0862, 300.0628, 241.0499, 227.2940, 202.6283, 185.0605, 167.4757, 139.0032, 121.0302 | Tetrahydroxymethoxy-flavone | WSR |
| 51 | 25.55 | C_17_H_14_O_8_ | [M-H]- | 345.0598 | -3.77 | 345.0607, 330.0371, 315.0136, 287.0186, 269.0086, 243.0285, 233.0084, 215.0346, 190.9982, 178.9980, 164.9823, 161.0237, 149.0238, 139.0033, 133.0290, 125.0238, 121.0291, 110.0005 | viscidulin III | RGF |
| 52 | 25.99 | C_22_H_36_O_12_ | [M-H]- | 491.2119 | -2.04 | 537.1957, 473.0452, 295.0811, 263.0767, 253.0603, 221.0657, 193.0497, 175.0393, 167.1071, 160.0159, 134.1369, 113.0240 | Jasminoside Q | GF |
| 53 | 26.02 | C_15_H_10_O_6_ | [M-H]- | 285.0395 | -1.40 | 285.0394, 267.0289, 239.0342, 185.0600, 166.9980, 137.0239, 117.0341, 111.0084, 93.0342 | Kaempferol or isomer | WSR |
| 54 | 26.12 | C_22_H_20_O_12_ | [M-H]- | 475.0861 | -3.37 | 405.5917, 385.0921, 355.0819, 311.0552, 299.0550, 284.0311, 269.0449, 195.3223, 165.9899, 113.0238, 85.0291 | trihydroxy-methoxy-flavone-7-*O*-glucuronide | WSR |
| 55 | 26.22 | C_16_H_12_O_7_ | [M-H]- | 315.05 | -1.59 | N/A | tetrahydroxymethoxy-flavone | RGF |
| 56 | 26.62 | C_32_H_40_O_17_ | [M-H]- | 695.2159 | -4.03 | 659.1921, 483.1460, 461.1655, 415.4592, 360.5443, 337.0896, 319.0801, 265.0713, 227.3050, 193.0499, 175.0394, 160.0158, 153.0551, 113.0240 | 6″-*O*-[(*E*)-*p*-Coumaroyl] genipin gentiobioside or isomer | RGF |
| 57 | 26.62 | C_31_H_32_O_16_ | [M-H]- | 659.1594 | -2.73 | 659.1926, 495.1258, 483.1474, 449.0147, 353.0872, 337.0891, 319.0791, 233.0666, 205.1754, 191.0553, 173.0449, 161.0450, 153.0551, 135.0449, | 3,5-Di-*O*-caffeoyl-4-*O*-(3-hydroxy-3-methyl) glutaroylquinic acid | RGF |
| 58 | 27.21 | C_27_H_28_O_13_ | [M-H]- | 559.1437 | -2.68 | 559.1423, 541.1328, 515.1181, 485.1093, 473.1071, 455.0961, 439.0990, 395.0762, 365.0639, 335.0551, 307.0599, 279.0647, 249.1319, 189.0182, 145.0289, 113.0239 | 3-*O*-Sinapoyl-5-*O*-caffeoylquinic acid | RGF |
| 59 | 27.45 | C_34_H_44_O_19_ | [M-H]- | 755.2366 | -4.37 |  | 6″-*O*-[trans-Sinapoyl] genipin gentiobioside | RGF |
| 60 | 27.75 | C33H42O18 | [M-H]- | 725.2269 | -3.31 | 725.1825, 659.6229, 635.1479, 549.1504, 469.7846, 359.1490, 349.5188, 279.9072, 227.3043, 197.9852, 190.7857, 168.1345, | 6″-*O*-[trans-Feruloyl] genipin gentiobioside | RGF |
| 61 | 27.99 | C_27_H_36_O_12_ | [M-H]- | 551.2122 | -1.27 | 551.2125, 528.9066, 436.7954, 385.1138, 325.0918, 311.0765, 295.0812, 281.0653, 265.0707, 250.0472, 237.0759, 223.0603, 205.0498, 190.0264, 175.0029, 164.0472, 149.0238, 135.0445, 121.0291, 101.0240 | 6′-*O*-trans-Sinapoyljasminoside L | WSR |
| 62 | 28.78 | C_21_H_18_O_11_ | [M-H]- | 445.0757 | -3.15 | 445.1134, 282.0526, 269.0448, 239.0343, 227.3019, 169.0651, 113.0242, 85.0290 | Baicalin | RGF |
| 63 | 29.05 | C_27_H_28_O_13_ | [M-H]- | 559.1437 | -2.68 | 559.2326, 487.0445, 469.0348, 414.1740, 381.0186, 353.0228, 329.0243, 26.0446, 204.7193, 173.0449, 123.5603, 110.9753 | 4-Sinapoyl-5-caffeoylquinic acid | RGF |
| 64 | 29.8 | C_44_H_64_O_24_ | [M-H]- | 975.3662 | -4.82 | N/A | Crocin I | WSR |
| 65 | 30.3 | C_21_H_20_O_11_ | [M-H]- | 447.0913 | -3.36 | 447.2342, 422.6166, 379.8326, 349.0588, 289.5559, 271.0604, 243.0655, 227.0708, 152.0109, 124.0161, 85.0291 | Dihydrobaicalin | WSR |
| 66 | 30.53 | C_16_H_12_O_7_ | [M-H]- | 315.0501 | -1.27 | 315.0501, 283.0241, 243.0646, 227.2944, 148.0160, 122.9816, 107.0134 | Tetrahydroxymethoxy-flavone | WSR |
| 67 | 32.26 | C_21_H_18_O_11_ | [M-H]- | 445.0756 | -3.37 | 445.0768, 269.0445, 225.0549, 197.0601, 171.0446, 113.0240 | Glychionide A | WSR |
| 68 | 32.92 | C_16_H_12_O_7_ | [M-H]- | 315.0501 | -1.27 | 315.0506, 300.0267, 271.0236, 243.0291, 217.0134, 176.0111, 148.0160, 124.0162, 107.0134 | Tetrahydroxymethoxy-flavone | WSR |
| 69 | 33.01 | C_22_H_22_O_10_ | [M-H]- | 445.1114 | -4.72 | 445.1123, 430.0892, 269.0444, 239.0341, 197.0600, 171.0445 | Oroxylin A 7-*O*-*D*-glucuronide isomer | WSR |
| 70 | 34.07 | C_21_H_18_O_11_ | [M-H]- | 445.0757 | -3.15 | 445.0748, 312.0797, 269.0445, 241.049 | Norwogonin-8-*O*-glucuronide | WSR |
| 71 | 34.66 | C_22_H_20_O_12_ | [M-H]- | 475.0862 | -3.16 | 375.1768, 299.0540, 284.0316, 271.7328, 236.2720, 227.3036, 124.9877 | 5,7,2'-trihydroxy-6-methoxy-flavone-7-*O*-glucuronide | WSR |
| 72 | 35.14 | C_22_H_22_O_11_ | [M-H]- | 461.1072 | -2.60 | 461.1075, 446.0844, 358.0091, 330.2234, 299.0552, 283.0239, 239.0342, 211.0393, 173.0601, 155.0497, 127.0548 | 5, 7-dihydroxy-6-methoxy-flavanone-7-*O*-glucuronide | WSR |
| 73 | 35.43 | C_21_H_18_O_10_ | [M-H]- | 429.0808 | -3.26 | N/A | Chrysin-7-*O*-β-*D*-glucoronide | WSR |
| 74 | 36.79 | C_22_H_20_O_11_ | [M-H]- | 459.0914 | -3.05 | N/A | Oroxylin A-7-*O*-glucuronide | RGF |
| 75 | 37.53 | C_38_H_54_O_19_ | [M-H]- | 859.3203 | -3.84 | N/A | Crocin II | WSR |
| 76 | 38.49 | C_18_H_16_O_8_ | [M-H]- | 359.0759 | -2.23 | 359.1494, 329.0301, 314.0056, 286.0114, 250.3101, 194.9929, 139.8432, 133.0293 | 5,2',5'-trihydroxy-6,7, 8-trimethoxy-flavonoids | WSR |
| 77 | 38.84 | C_21_H_20_O_10_ | [M-H]- | 431.097 | -1.86 | 431.0974, 423.1322, 330.9991, 269.0446, 227.2928, 195.0448, 155.2372, 110.0005 | Dihydroxyflavanone-*O*-glucuronide | WSR |
| 78 | 38.86 | C_21_H_20_O_10_ | [M-H]- | 431.097 | -2.09 | 431.0974, 423.1322, 330.0991, 269.0446, 227.2928, 195.0448, 155.2372, 110.0005 | Trihydroxydihydroflavone-*O*-glucuronide | WSR |
| 79 | 39.4 | C_15_H_10_O_6_ | [M-H]- | 285.0395 | -1.40 | 285.0395, 267.0286, 241.0496, 213.0548, 185.0601, 171.0444, 151.0031, 133.0290, 125.0239 | Kaempferol or isomer | WSR |
| 80 | 39.46 | C_21_H_18_O_11_ | [M-H]- | 445.0757 | -3.15 | 445.0748, 312.0797, 269.0445, 223.0393, 197.0601 | Baicalein-6-*O*-glucuronide isobaric conformation | WSR |
| 81 | 39.7 | C_22_H_22_O_11_ | [M-H]- | 461.1068 | -3.47 | 461.01073, 446.0847, 325.7389, 299.0553, 284.0319, 269.0410, 239.8212, 227.3004, 173.0605, 155.0498, 113.1963 | Dihydroxy-methoxy-flavanone-*O*-glucuronide | WSR |
| 82 | 39.91 | C_22_H_20_O_11_ | [M-H]- | 459.0913 | -3.27 | N/A | Wogonoside | WSR |
| 83 | 40.94 | C_15_H_10_O_5_ | [M-H]- | 269.0443 | -2.60 | 269.0445, 254.0578, 225.0551, 197.0599, 171.0443, 136.9874 | Norwogonin | WSR |
| 84 | 41.52 | C_23_H_22_O_12_ | [M-H]- | 489.1015 | -3.68 | 489.2654, 408.5948, 327.2173, 298.0477, 227.3035, 211.1335, 156.1382, 101.0241 | 5, 7-dihydroxy-6, 8-dimethoxy-flavone-7-*O*-glucuronide | WSR |
| 85 | 41.97 | C_16_H_12_O_6_ | [M-H]- | 299.0547 | -3.01 | 299.0563, 284.0318, 267.0313, 256.0375, 236.9423, 227.0706, 211.0394, 165.0188, 133.0290 | trihydroxymethoxy-flavone or isomer | RGF |
| 86 | 42.19 | C_27_H_36_O_11_ | [M-H]- | 535.2167 | -2.24 | N/A | 6'-*O*-trans-sinapoyl jasminoside A or isomer | WSR |
| 87 | 42.19 | C_18_H_16_O_8_ | [M-H]- | 359.0759 | -2.23 | 395.0760, 344.0525, 329.0292, 301.0344, 284.0317, 342.0209, 198.0315, 180.9771, 152.9823, 124.9875 | 5,2',5'-trihydroxy-6,7, 8-trimethoxy-flavonoids | WSR |
| 88 | 42.48 | C_17_H_14_O_7_ | [M-H]- | 329.0653 | -2.74 | 329.0656, 314.0421, 299.0187, 271.0240, 227.0342, 199.0392, 180.0058, 165.9900, 137.9953, 110.0004 | trihydroxy-dimethoxy-flavoneor isomer | WSR |
| 89 | 42.94 | C_16_H_12_O_6_ | [M-H]- | 299.0547 | -3.01 | 299.0551, 284.0315, 271.0600, 256.0368, 239.0343, 227.0704, 211.0392, 200.0469, 181.9847, 153.9901, 133.0289 | trihydroxymethoxy-flavone | WSR |
| 90 | 43.44 | C_15_H_10_O_5_ | [M-H]- | 269.0442 | -2.97 | 269.0445, 241.0498, 223.0392, 197.0600'169.0652, 136.9875 | Baicalein | RGF |
| 91 | 43.51 | C_28_H_34_O_14_ | [M-H]- | 593.1857 | -2.19 | 593.0120, 575.0061, 509.0237, 457.9223, 401.0113, 364.9731, 340.9736, 324.9781, 267.0289, 223.0603, 207.0652, 164.0472, 149.0238, 101.0239 | 6'-*O*-sinapoylgeniposide | WSR |
| 92 | 43.62 | C_17_H_14_O_7_ | [M-H]- | 329.0655 | -2.13 | 329.0659, 314.0422, 299.0188, 271.0239, 255.0292, 227.0342, 183.0443, 143.0497, 124.9875 | Trihydroxy-dimethoxy-flavone | WSR |
| 93 | 44.13 | C_16_H_12_O_6_ | [M-H]- | 299.055 | -2.01 | 299.0556, 284.0316, 256.0374, 227.3013, 211.0395, 181.9853, 153.9902, 125.9955 | trihydroxymethoxy-flavone or isomer | WSR |
| 94 | 44.69 | C_17_H_14_O_7_ | [M-H]- | 329.0657 | -1.52 | 329.0656, 314.0422, 299.0186, 271.0240, 243.0300, 227.0345, 199.0389, 143.0495, 110.0007 | trihydroxy-dimethoxy-flavone or isomer | RGF |
| 95 | 45.38 | C_32_H_44_O_14_ | [M-H]- | 651.264 | -2.00 | 651.1663, 495.5093, 409.0909, 337.0705, 327.1591, 283.1693, 239.1796, 221.0656 | all*-*trans-crocetin di-β-*D*-glucosyl eater | RGF |
| 96 | 46.11 | C_44_H_64_O_24_ | [M+HCOO]- | 1021.371 | -5.29 | N/A | Crocin I isomer | WSR |
| 97 | 46.28 | C_17_H_14_O_7_ | [M-H]- | 329.0655 | -2.13 | 329.2324, 314.0423, 299.0186, 271.0233, 243.0282, 229.1436, 211.1331, 183.1380, 171.1020, 157.1228, 139.1123, 127.1124 | trihydroxy-dimethoxy-flavone or isomer | WSR |
| 98 | 47.44 | C_18_H_16_O_7_ | [M-H]- | 343.0807 | -3.21 | 343.0818, 328.0581, 313.0345, 285.0398, 269.2064, 227.2984, 180.0054, 164.8722, 136.9876, 116.2833 | Skullcapflavone | WSR |
| 99 | 47.49 | C_18_H_16_O_8_ | [M-H]- | 359.0757 | -2.78 | 359.1494, 329.0293, 301.0346, 286.0109, 244.0737, 207.1902, 168.1547, 136.9323 | 5,2',5'-trihydroxy-6,7, 8-trimethoxy-flavonoids | RGF |
| 100 | 47.54 | C_27_H_36_O_11_ | [M-H]- | 535.2167 | -2.24 | 859.3203, 813.3165, 651.2277, 537.1599, 475.0867, 445.0762, 323.0975, 197.8073, 125.8728 | 6'-*O*-trans-sinapoyl jasminoside A or isomer | WSR |
| 101 | 47.66 | C_16_H_12_O_5_ | [M-H]- | 283.0598 | -3.18 | 283.0601, 268.0366, 239.0339, 211.0390, 184.0522, 163.0030, 137.9953, 110.0005 | Wogonin | WSR |
| 102 | 47.94 | C_15_H_10_O_4_ | [M-H]- | 253.0495 | -2.37 | 253.0497, 227.3000, 209.1541, 183.9131, 143.0499, 107.0137 | Ch*r*ysin | WSR |
| 103 | 48.27 | C_17_H_14_O_6_ | [M-H]- | 313.0705 | -2.24 | 313.0708, 298.0472, 283.0237, 269.0450, 255.0290, 239.0340, 211.0390, 183.0440, 164.9822, 136.9875, 117.0343 | Dihydroxy-dimethoxy-flavone | WSR |
| 104 | 48.68 | C_19_H_18_O_8_ | [M-H]- | 373.0909 | -4.02 | 373.0914, 358.0681, 343.0446, 328.0215, 300.0265, 285.0033, 257.0083, 194.9928, 151.9744, 133.0290 | Skullcapflavone II | WSR |
| 105 | 48.8 | C_16_H_12_O_5_ | [M-H]- | 283.06 | -2.47 | 283.0601, 268.0366, 239.0339, 211.0390, 184.0522, 163.0030, 137.9953, 110.0005 | Oroxylin A | WSR |
| 106 | 49.13 | C_30_H_18_O_10_ | [M-H]- | 537.0807 | -2.79 | 537.0807, 529.2979, 417.0234, 391.0446, 373.0342, 319.0600, 270.9875, 245.0082, 217.0134, 145.0289 | 8,8''-Bibaicalein | WSR |
| 107 | 49.13 | C_17_H_14_O_6_ | [M-H]- | 313.0705 | -2.24 | 313.0706, 298.0473, 283.0239, 255.0296, 227.2955, 183.0444, 164.9822, 136.9877 | Dihydroxy-dimethoxy-flavone | WSR |
| 108 | 50.45 | C_18_H_16_O_7_ | [M-H]- | 343.0807 | -3.21 | 343.0816, 328.0580, 313.0345, 298.0108, 270.0160, 242.0212, 198.0318, 179.9693, 151.9747, 123.9799, 85.9091 | tenaxin I | RGF |
| 109 | 50.6 | C_48_H_60_O_22_ | [M-H]- | 987.3451 | -4.76 | N/A | Neocrocin B/C/D/E | RGF |
| 110 | 51.47 | C_32_H_44_O_14_ | [M-H]- | 651.2631 | -3.38 | N/A | Crocin-III or isomer | RGF |
| 111 | 51.69 | C_32_H_44_O_14_ | [M-H]- | 651.2632 | -3.22 | 327.159 | Crocin-III or isomer | RGF |
| 112 | 52.2 | C_32_H_44_O_14_ | [M-H]- | 651.2631 | -3.38 | N/A | Crocin-III or isomer | RGF |
| 113 | 57 | C_20_H_24_O_4_ | [M-H]- | 327.1591 | -1.53 | 327.159 | Crocetin |  |

## **Table S4** Identification of compounds in PW4 by UPLC-Orbitrap HRMS

| **No** | **t_R_**  **(min)** | **Formula** | **Selection ion** | **Measured mass (m/z)** | **Error (ppm)** | **Fragmentations** | **Identification** | **Source** |
| --- | --- | --- | --- | --- | --- | --- | --- | --- |
| 1 | 1.37 | C_7_H_12_O_6_ | [M-H]- | 191.055 | -3.14 | 191.0551, 184.6895, 179.6002, 173.0081, 142.6267, 129.0187 | Quinic acid | GFP |
| 2 | 2.45 | C_16_H_22_O_11_ | [M-H]- | 389.1066 | -4.63 | 389.1064, 303.0144, 273.8625, 227.0545, 209.0443, 183.0651, 165.0547, 147.0443, 139.0392 | Deacetylasperulosidic acid | GFP |
| 3 | 3.13 | C_16_H_22_O_10_ | [M-H]- | 373.1118 | -4.56 | N/A | Gardoside | GFP |
| 4 | 3.3 | C_16_H_24_O_11_ | [M-H]- | 391.1225 | -3.84 | 391.1232, 357.5748, 229.0714, 185.0814, 156.4031, 140.1040 | Shanzhiside isomer | GFP |
| 5 | 3.66 | C_9_H_8_O_4_ | [M-H]- | 179.034 | -2.79 | 179.0340, 161.0235, 151.0394, 134.9872, 133.0287, 122.0368 | Caffeic acid | WSR |
| 6 | 3.95 | C_17_H_26_O_11_ | [M-H]- | 405.138 | -4.20 | N/A | Shanzhiside methyl ester | GFP |
| 7 | 4.16 | C_7_H_6_O_4_ | [M-H]- | 153.0185 | -1.96 | 153.0185, 109.0289 | Protocatechuic acid | WSR |
| 8 | 4.16 | C_16_H_24_O_11_ | [M-H]- | 391.1223 | -4.35 | 391.1227, 369.0520, 260.1431, 229.0706, 193.0498, 185.0810, 179.0549, 167.0705, 149.0600, 127.0394, 101.0238 | Shanzhiside | GFP |
| 9 | 4.33 | C_16_H_22_O_10_ | [M-H]- | 373.1118 | -4.56 |  | Geniposidic acid | GFP |
| 10 | 5.34 | C_16_H_18_O_9_ | [M-H]- | 353.0865 | -2.27 | 353.0865, 305.7931, 229.0718, 191.0553, 177.0486, 161.0241, 135.0445 | Chlorogenic acid | WSR |
| 11 | 5.37 | C_17_H_24_O_11_ | [M+HCOO]- | 449.1282 | -2.89 | 376.7819, 339.2025, 282.1960, 241.0708, 223.0605, 205.0498, 191.0345, 177.0550, 163.0398, 149.0604, 139.0395, 133.0655, 121.0290, 101.0240 | 6α-hydroxygeniposide | GFP |
| 12 | 6.87 | C_17_H_24_O_11_ | [M+HCOO]- | 449.1283 | -2.67 | 449.1300, 424.3920, 388.3614, 373.1133, 321.3132, 247.4908, 241.0716, 223.0608, 193.0500, 185.0808, 167.0708, 149.0601, 135.0447, 127.0396, 123.0448, 109.0293, 101.0240 | Gardenoside | GFP |
| 13 | 7.04 | C_16_H_24_O_10_ | [M-H]- | 375.1282 | -2.40 | 375.1294, 302.6763, 242.5241, 222.7178, 213.0760, 195.0660, 169.0863, 151.0759, 133.0653, 125.0604, 113.0240, 107.0498, 101.0240 | Mussaenosidic acid | GFP |
| 14 | 7.22 | C_16_H_26_O_8_ | [M-H]- | 345.154 | -2.61 | 345.1176, 315.3869 | Jasminoside B | GFP |
| 15 | 8.99 | C_17_H_24_O_11_ | [M-H]- | 403.123 | -2.48 | 403.1612, 347.3356, 295.3862, 227.2952, 191.0552, 101.0239 | 6β-hydroxygeniposide | GFP |
| 16 | 9.55 | C_16_H_26_O_8_ | [M-H]- | 345.1541 | -2.32 | 315.9545, 282.9948, 240.4010, 218.1541, 201.3517, 181.0504, 165.0914, 144.0449, 121.1019, 119.0347, 113.0239, 101.0239 | Picrocrocinic acid | GFP |
| 17 | 10.68 | C_16_H_18_O_9_ | [M-H]- | 353.0862 | -3.12 | N/A | Neochlorogenic acid | GFP |
| 18 | 11.72 | C_16_H_18_O_9_ | [M-H]- | 353.0863 | -2.83 | N/A | Chlorogenic acid | GFP |
| 19 | 12.18 | C_7_H_6_O_4_ | [M-H]- | 153.0188 | 0.00 | 153.0187, 135.0082, 109.0290, 83.8022 | Protocatechuic acid-isomer | WSR |
| 20 | 12.33 | C_15_H_12_O_7_ | [M-H]- | 303.0497 | -2.64 | 303.0502, 285.0395, 275.0555, 259.0606, 241.0498, 227.2993, 217.0498, 193.0495, 177.0186, 165.0550, 149.0238, 133.0290, 125.0239, 107.0135 | 3,6,7,2',6 '-pentahydroxyflavanones | WSR |
| 21 | 12.48 | C_23_H_34_O_15_ | [M-H]- | 549.1806 | -2.37 | 595.1544, 505.1264, 475.1132, 385.0812, 355.0715, 299.0811, 264.9399, 225.0763, 207.0663, 192.5083, 147.0446, 123.0447, 101.0239 | Genipin 1-gentiobioside | GFP |
| 22 | 13.01 | C_17_H_22_O_10_ | [M-H]- | 385.1125 | -2.60 | 385.1138, 325.0915, 247.0602, 223.0603, 205.0498, 190.0263, 164.0472, 149.0238, 121.0290, 101.0240 | Sinapyglucoside | GFP |
| 23 | 13.49 | C_17_H_24_O_10_ | [M+HCOO]- | 433.1329 | -3.92 | 433.1132, 387.0013, 36.9926, 340.9966, 335.0128, 303.0509, 297.0074, 260.9717, 2509865, 225.0080, 206.9967, 177.0190, 169.0869, 137.6033, 125.0239, 107.0135 | Geniposide | GFP |
| 24 | 15.21 | C_15_H_10_O_7_ | [M-H]- | 301.034 | -2.99 | 301.0343, 283.0232, 257.0456, 229.0501, 215.0344, 205.0495, 193.0134, 187.0394, 163.0037, 155.0340, 151.0031, 139.0396, 125.0239, 121.0289, 107.0134 | Quercetin | WSR |
| 25 | 15.57 | C_26_H_28_O_14_ | [M-H]- | 563.1386 | -2.66 | 563.1385, 545.1282, 503.1179, 485.1079, 473.1072, 455.0969, 443.0969, 425.0857, 413.0868, 395.0759, 383.0760, 365.0652, 353.0654, 337.0708, 352.0708, 307.0602, 297.0758, 283.0602, 267.0652, 233.0448, 205.0496, 191.0340, 1661.0238, 135.0446, 117.0342 | Schaftoside | WSR |
| 26 | 15.64 | C_16_H_26_O_7_ | [M-H]- | 375.1646 | -2.40 | 241.3907, 208.7640, 197.0443, 167.1071, 161.0454, 152.0838, 130.8622, 113.0242, 101.0239 | Picrocrocin | GFP |
| 27 | 17.78 | C_27_H_30_O_16_ | [M-H]- | 609.1438 | -2.95 | 609.1428, 473.2955, 444.9907, 354.5961, 300.0265'271.0236, 255.0291, 227.2985, 136.7286, 113.0241 | Rutin or isomer | GFP |
| 28 | 17.9 | C_21_H_20_O_12_ | [M-H]- | 463.0861 | -3.46 | 301.0343, 287.0550, 227.2970, 213.5822, 191.0163, 166.9983, 153.0193, 139.0035, 119.0500 | Carthamidin-7-*O*-glucuronide | WSR |
| 29 | 18.62 | C_27_H_30_O_16_ | [M-H]- | 609.1437 | -3.12 | 609.1439, 563.2678, 343.0446, 300.0264, 271.0238, 255.0289, 243.0290, 227.0342, 211.0393, 178.9978, 151.0030, 135.0078, 107.0135 | Rutin or isomer | GFP |
| 30 | 19.32 | C_21_H_20_O_12_ | [M-H]- | 463.0866 | -2.38 | 463.0861, 300.0264, 287.0445, 271.0238, 255.0289, 243.0288, 227.0342, 199.0392, 178.9977, 163.0027, 151.0030, 108.0211 | Isoquercetin | GFP |
| 31 | 19.45 | C_21_H_18_O_12_ | [M-H]- | 461.0706 | -3.04 | 296.2546, 285.0394, 267.0299, 239.0340, 185.0604, 136.9878, 113.0239 | Scutellarin | WSR |
| 32 | 19.75 | C_26_H_28_O_13_ | [M-H]- | 547.1432 | -3.66 | 547.1438, 529.1321, 487.1230, 457.1125, 427.1020, 409.0920, 379.0815, 367.0811, 349.0703, 337.0708, 321.0761, 309.0760, 293.0807, 281.0809, 267.0653, 252.0786, 235.0758, 203.0340, 163.0395, 145.0288, 119.0498 | Chrysin 6-*C*-arabinoside 8-*C*-glucoside | WSR |
| 33 | 20 | C_23_H_24_O_13_ | [M-H]- | 507.1127 | -2.37 | 507.1126, 492.0889, 473.6878, 345.0605, 330.0372, 345.0137, 287.0186, 271.0244, 233.0083, 215.0337, 190.9972, 164.9823, 149.0238, 136.9877, 110.0005 | Viscidulin III-2′-*O*-glucoside | WSR |
| 34 | 20.07 | C_16_H_26_O_8_ | [M-H]- | 345.1543 | -1.74 | 345.1543, 306.9102, 285.3172, 227.2973, 216.7344, 207.8058, 165.0917, 138.6624, 123.4476, 119.0344, 101.0241 | Jasminoside G | GFP |
| 35 | 20.59 | C_21_H_20_O_12_ | [M-H]- | 463.0864 | -2.81 | 300.0273, 287.0443, 239.0340, 227.3049, 186.0638, 166.9980, 139.0032, 113.0241 | Carthamidin 7-*O*-glucuronide isomer | GFP |
| 36 | 21.7 | C_15_H_10_O_7_ | [M-H]- | 301.0344 | -1.33 | 301.0348, 283.0243, 273.0407, 269.6783, 269.6783, 255.2387, 227.3019, 217.0137, 201.0191, 180.0998, 166.9977, 151.0031, 149.0241, 139.0033, 135.0444, 125.0242, 119.0497, 111.0084, 107.0136 | 3,5,7,2',6'-Pentahydroxy flavones | GFP |
| 37 | 21.72 | C_29_H_36_O_15_ | [M-H]- | 623.1959 | -2.73 | 623.1967, 490.8506, 461.1646, 455.9095, 418.9419, 284.0324, 227.2933, 219.6621, 190.9978, 175.0390, 161.0238, 133.0289, 113.0241, 109.0289 | Verbascoside | WSR |
| 38 | 21.85 | C_23_H_24_O_12_ | [M-H]- | 491.1176 | -2.85 | 491.1164, 461.0717, 387.0730, 371.0746, 353.0651, 329.0656, 315.0500, 300.0265, 285.0395, 271.0241, 255.0293, 227.0339, 180.0059, 164.9823, 151.0033, 133.0289, 110.0004 | Trihydroxy-dimethoxy-flavone glucoside | WSR |
| 39 | 21.97 | C_26_H_28_O_13_ | [M-H]- | 547.1435 | -3.11 | 547.1437, 529.1326, 487.1214, 469.1104, 457.1126, 427.1026, 427.0912, 397.0913, 367.0811, 349.0704, 337.0708, 321.0756, 309.0758, 295.0602, 281.0810, 267.0656, 252.0782, 237.0911, 191.0343, 163.0394, 145.0291, 119.0497 | Chrysin 6-*C*-glucoside 8-*C*-arabinoside | WSR |
| 40 | 22.02 | C_25_H_24_O_12_ | [M-H]- | 515.1176 | -2.72 | 353.0879, 266.2159, 245.2538, 227.2979, 179.0341, 173.0451, 168.5729, 135.0448, 123.6833 | 3,4-Dicaffeoyl quinic acid | GFP |
| 41 | 22.28 | C_25_H_24_O_12_ | [M-H]- | 515.1179 | -2.14 | 353.0871, 335.0766, 227.2978, 205.0493, 191.0555, 173.0449, 161.0240, 155.02345, 135.0446, 122.6317, 111.0448 | 3,5-Dicaffeoyl quinic acid | GFP |
| 42 | 22.75 | C_26_H_28_O_13_ | [M-H]- | 547.1434 | -3.29 | 547.1439, 457.1124, 427.1021, 397.0815, 367.0811, 281.0810, 191.0342 | Chrysin 6-*C*-glucoside 8-*C*-arabinoside isomer | WSR |
| 43 | 22.86 | C_27_H_34_O_14_ | [M-H]- | 581.1852 | -3.27 | N/A | Trihydroxydihydrochalcone-3'-*C*-glucoside-6'-*O*-glucoside or isomer | WSR |
| 44 | 22.97 | C_16_H_12_O_7_ | [M-H]- | 315.05 | -1.59 | 315.0509, 300.0266, 243.0654, 227.2963, 165.9906, 146.5380, 121.6354, 110.0003 | Tetrahydroxymethoxy-flavone | WSR |
| 45 | 23.45 | C_29_H_36_O_15_ | [M-H]- | 623.1956 | -3.21 | 623.1617, 491.9513, 422.8155, 381.9150, 344.3590, 283.0243, 255.9695, 227.3043, 173.0597, 153.6253, 148.4463, 106.2644 | Isoacteoside | WSR |
| 46 | 23.57 | C_21_H_20_O_9_ | [M-H]- | 415.1017 | -2.89 | 415.1006, 295.0597, 279.4052, 267.0647, 256.0528, 227.3048, 216.7598, 156.3249, 149.0240, 119.0500, 104.7206 | Chrysin 8-*C*-glucoside | WSR |
| 47 | 23.57 | C_21_H_20_O_9_ | [M-H]- | 415.1017 | -2.89 | 415.1023, 379.0776, 337.0708, 325.0709, 295.0603, 267.0655, 253.0498, 223.0754, 181.0654, 145.0289, 121.0292 | Chrysin 6-*C*-glucoside | WSR |
| 48 | 23.65 | C_21_H_20_O_10_ | [M-H]- | 431.0969 | -2.09 | 431.0973, 385.0247, 269.0446, 241.0508, 197.0602, 165.9904, 139.0398, 110.0006 | Apigenin 7-glucoside | WSR |
| 49 | 24.3 | C_22_H_22_O_10_ | [M-H]- | 445.1123 | -2.70 | 445.1127, 430.0896, 359.1273, 283.0606, 267.0289, 239.0341, 227.2950, 195.0443, 171.0448, 145.0292, 136.9880 | Oroxylin A 7-*O*-*D*-glucuronide | WSR |
| 50 | 24.46 | C_16_H_12_O_7_ | [M-H]- | 315.0502 | -0.95 | 315.0524, 309.0291, 300.0257, 278.1763, 253.0500, 227.3002, 209.0600, 185.3759, 145.0291, | Tetrahydroxymethoxy flavone | WSR |
| 51 | 25.6 | C_17_H_14_O_8_ | [M-H]- | 345.0598 | -3.77 | 345.0606, 330.0372, 315.01387, 259.5018, 227.2945, 208.2302, 196.1604, 164.9824, 149.0239, 139.0037, 110.0005 | Viscidulin III | WSR |
| 52 | 25.97 | C_22_H_36_O_12_ | [M-H]- | 491.2117 | -2.44 | 537.1954, 429.0072, 369.1073, 347.1141, 285.0408, 256.6608, 227.2995, 205.0500, 167.0343, 152.0110, 123.0445, 108.0212 | Jasminoside Q | GFP |
| 53 | 26.03 | C_15_H_10_O_6_ | [M-H]- | 285.0394 | -1.75 | 285.0393, 267.0290, 239.0341, 213.0548, 185.0601, 166.9979, 137.0238, 117.0341, 111.0082 | Kaempfero or isomer | WSR |
| 54 | 26.15 | C_22_H_20_O_12_ | [M-H]- | 475.0859 | -3.79 | 475.1620, 299.0558, 281.0455, 269.0450, 229.6120, 166.7318, 153.0190, 143.4306, 109.8662, 96.9597 | Trihydroxy-methoxy-flavone-7-*O*-glucuronide | WSR |
| 55 | 26.27 | C_16_H_12_O_7_ | [M-H]- | 315.0501 | -1.27 | 315.0510, 309.3836, 300.0272, 290.4504, 272.0327, 250.1619, 243.0658, 227.0341, 199.0756, 165.0187, 149.0239, 139.0396, 121.0292, 109.0292 | Tetrahydroxymethoxy-flavone | WSR |
| 56 | 26.55 | C_32_H_40_O_17_ | [M-H]- | 695.2161 | -3.74 | 695.2153, 649.1996, 548.6833, 469.1333, 451.1226, 409.1119, 367.1021, 207.0811, 265.0706, 225.0762, 207.0656, 163.0394, 145.0289, 123.0447, 101.0240 | 6''-*O*-[(*E*)-*p*-Coumaroyl] genipin gentiobioside or isomer | GFP |
| 57 | 26.74 | C_31_H_32_O_16_ | [M-H]- | 659.1595 | -2.58 | 659.1941, 512.8961, 483.1477, 461.0767, 337.0900, 319.0794, 227.3023, 191.0557, 173.0450, 153.0554, 135.0451, 123.0448 | 3,5-Di-*O*-Caffeoyl-4-*O*-(3-hydroxy-3-methyl) glutaroylquinic acid | GFP |
| 58 | 27.16 | C_27_H_28_O_13_ | [M-H]- | 559.1442 | -1.79 | 559.1446, 541.1343, 511.0767, 485.1072, 443.0978, 367.0828, 353.0654, 323.0554, 295.0602, 267.0651, 227.2988, 191.0348, 169.0135, 151.0029, 111.0085, 101.0242 | 3-*O*-Sinapoyl-5-*O*-caffeoylquinic acid | GFP |
| 59 | 27.51 | C_34_H_44_O_19_ | [M-H]- | 755.2366 | -4.37 | N/A | 6''-*O*-[trans-Sinapoyl] genipin gentiobioside | GFP |
| 60 | 27.82 | C_33_H_42_O_18_ | [M-H]- | 725.2267 | -3.59 | 725.1806, 665.1620, 635.1504, 549.1507, 489.1300, 458.1158, 441.1099, 369.0874, 359.1488, 337.0710, 305.0435, 281.0805, 247.0599, 227.2987, 205.0502, 190.0266, 177.0548, 149.0244, 121.0291, 113.0242 | 6''-*O*-[trans-Feruloyl] genipin gentiobioside | GFP |
| 61 | 27.99 | C_27_H_36_O_12_ | [M-H]- | 551.2123 | -1.09 | 415.1868, 285.0385, 269.0453, 246.7270, 210.8760, 205.0502, 190.0264, 138.7590 | 6'-*O*-trans-Sinapoyljasminoside L | GFP |
| 62 | 28.78 | C_21_H_18_O_11_ | [M-H]- | 445.0756 | -3.37 | 445.1134, 398.2031, 283.0613, 269.0450, 239.0349, 197.0600, 169.0655, 113.0241, 85.0293 | Baicalin | WSR |
| 63 | 29.05 | C_27_H_28_O_13_ | [M-H]- | 559.1438 | -2.50 | 559.0349, 469.0726, 407.0349, 382.9981, 353.0236, 329.0239, 288.9774, 269.0446, 239.0336, 200.9617, 173.0448, 149.0239, 110.9753 | 4-Sinapoyl-5-caffeoylquinic acid | GFP |
| 64 | 29.85 | C_44_H_64_O_24_ | [M-H]- | 975.3672 | -3.79 | 975.3632, 872.7020, 693.2770, 383.1190, 327.1588, 283.1694, 221.0659, 179.0556 | Crocin I | GFP |
| 65 | 30.3 | C_21_H_20_O_11_ | [M-H]- | 447.0913 | -3.36 | 447.0828, 385.0822, 271.0499, 226.0581, 197.0601, 171.04444, 113.0239 | Dihydrobaicalin | WSR |
| 66 | 30.59 | C_16_H_12_O_7_ | [M-H]- | 315.0502 | -0.95 | 315.0493, 165.9904, 157.1244, 126.5801, 101.1389 | Tetrahydroxymethoxy-flavone | WSR |
| 67 | 32.29 | C_21_H_18_O_11_ | [M-H]- | 445.0754 | -3.82 | 445.0750, 404.2270, 269.0445, 225.0544, 197.0600, 171.0445, 113.0239 | Glychionide A | WSR |
| 68 | 32.98 | C_16_H_12_O_7_ | [M-H]- | 315.05 | -1.59 | 315.0506, 300.0269, 289.0386, 240.8519, 227.2971, 220.4055, 178.5604, 139.0034 | Tetrahydroxymethoxy-flavone | WSR |
| 69 | 33.01 | C_22_H_22_O_10_ | [M-H]- | 445.1109 | -5.84 | 445.1115, 230.0887, 269.0444, 239.0341, 223.0391, 197.0599 | Oroxylin A 7-*O*-*D*-glucuronide isomer |  |
| 70 | 33.98 | C_21_H_18_O_11_ | [M-H]- | 445.0756 | -3.37 | 445.0740, 356.2047, 299.0236, 269.0444, 241.0499, 223.0393, 197.0601, 136.9874, 136.9874, 113.0239 | Norwogonin-8-*O*-glucuronide | WSR |
| 71 | 34.63 | C_22_H_20_O_12_ | [M-H]- | 475.0861 | -3.37 | 475.1628, 299.0551, 284.0316, 253.0493, 181.9852, 153.9902, 124.9874, 85.0290 | 5,7,2'-Trihydroxy-6-methoxy-flavone-7-*O*-glucuronide | WSR |
| 72 | 34.97 | C_22_H_22_O_11_ | [M-H]- | 461.1073 | -2.39 | 461.1075, 446.0840, 299.0551, 283.0238, 239.0345, 211.0393, 183.0444, 173.0601, 155.0497, 127.0549 | 5, 7-Dihydroxy-6-methoxy-flavanone-7-*O*-glucuronide | WSR |
| 73 | 35.43 | C_21_H_18_O_10_ | [M-H]- | 429.0806 | -3.73 | 253.0496, 227.2999, 209.0601, 143.0495, 113.0239 | Chrysin-7-*O*-β-*D*-glucoronide | WSR |
| 74 | 36.73 | C_22_H_20_O_11_ | [M-H]- | 459.0911 | -3.70 | 283.0601, 268.0366, 239.0330, 211.0391, 165.9900, 113.0239 | Oroxylin A-7-*O*-glucuronide | WSR |
| 75 | 37.45 | C_38_H_54_O_19_ | [M-H]- | 859.3203 | -3.84 | 327.1587, 283.1686, 239.1799, 227.2934, 192.5854, 144.5188, 113.0241, 101.0242 | Crocin II | GFP |
| 76 | 38.47 | C_18_H_16_O_8_ | [M-H]- | 359.0758 | -2.51 | 359.0756, 344.0526, 329.0292, 314.0058, 386.0109, 258.0168, 214.0266, 214.0266, 194.9927, 179.9691, 170.0369, 146.0371, 133.0289, 123.9799 | 5,2',5'-Trihydroxy-6,7, 8-trimethoxy-flavonoids | WSR |
| 77 | 38.84 | C_21_H_20_O_10_ | [M-H]- | 431.0968 | -2.32 | N/A | Dihydroxyflavanone-*O*-glucuronide | WSR |
| 78 | 38.89 | C_21_H_20_O_10_ | [M-H]- | 431.0968 | -2.32 | N/A | Trihydroxydihydroflavone-*O*-glucuronide | WSR |
| 79 | 39.29 | C_15_H_10_O_6_ | [M-H]- | 285.0394 | -1.75 | 285.0394, 267.0288, 241.0498, 227.2992, 213.0549, 185.0599, 171.0445, 151.0031, 133.0289, 125.0239, 107.0134 | Kaempferol or isomer | WSR |
| 80 | 39.4 | C_21_H_18_O_11_ | [M-H]- | 445.0754 | -3.82 | 445.0740, 356.2047, 299.0236, 269.0444, 241.0499, 223.0393, 197.0601, 136.9874, 113.0239 | Baicalein-6-*O*-glucuronide isobaric conformation | WSR |
| 81 | 39.69 | C_22_H_22_O_11_ | [M-H]- | 461.1066 | -3.90 | 461.1071, 446.0840, 367.3221, 299.0550, 284.0315, 270.0531, 239.0343, 211.0392, 183.0442, 165.9900, 155.0495, 110.0004 | Dihydroxy-methoxy-flavanone-*O*-glucuronide | WSR |
| 82 | 39.98 | C_22_H_20_O_11_ | [M-H]- | 459.0912 | -3.49 | 283.0601, 268.0367, 240.0421, 211.0388, 129.0187, 113.0239 | Wogonoside | WSR |
| 83 | 40.95 | C_15_H_10_O_5_ | [M-H]- | 269.0443 | -2.60 | 269.0433, 265.0045, 241.0497, 223.0395, 197.0596, 169.0651, 164.0063, 136.9875, 110.0004 | Norwogonin | WSR |
| 84 | 41.59 | C_23_H_22_O_12_ | [M-H]- | 489.1017 | -3.27 | 489.2670, 458.8478, 394.1623, 322.8294, 298.0464, 291.1967, 227.3038, 211.1332, 110.6720 | 5, 7-Dihydroxy-6, 8-dimethoxy-flavone-7-*O*-glucuronide | WSR |
| 85 | 41.96 | C_16_H_12_O_6_ | [M-H]- | 299.0552 | -1.34 | 299.0545, 284.0307, 256.0365, 227.0343, 211.0390, 181.8736, 165.0186, 148.7258, 133.0290, 110.0006 | Trihydroxymethoxy-flavone or isomer | WSR |
| 86 | 42.13 | C_27_H_36_O_11_ | [M-H]- | 535.2168 | -2.06 | N/A | 6'-*O*-Trans-sinapoyl jasminoside A or isomer | GFP |
| 87 | 42.21 | C_18_H_16_O_8_ | [M-H]- | 359.076 | -1.95 | 359.0760, 344.0524, 329.0293, 301.0353, 285.8124, 180.9774, 152.9825, 133.7075 | 5,2 ',5 '-Trihydroxy-6,7, 8-trimethoxy-flavonoids | WSR |
| 88 | 42.47 | C_17_H_14_O_7_ | [M-H]- | 329.0654 | -2.43 | 271.0620, 253.0496, 227.2933 | Trihydroxy-dimethoxy-flavoneor isomer | WSR |
| 89 | 42.93 | C_16_H_12_O_6_ | [M-H]- | 299.0547 | -3.01 | 299.0551, 284.0316, 271.0605, 256.0367, 239.0342, 227.0705, 211.0393, 200.0470, 181.9849, 153.9908, 133.0290 | Trihydroxymethoxy-flavone | WSR |
| 90 | 43.45 | C_15_H_10_O_5_ | [M-H]- | 269.0442 | -2.97 | 269.0445, 241.0498, 223.0393, 197.0601, 169.0653, 136.9875, 123.0085 | Baicalein | WSR |
| 91 | 43.45 | C_28_H_34_O_14_ | [M-H]- | 593.1856 | -2.36 | 593.0120, 547.0079, 509.0227, 452.0304, 340.9738, 324.9785, 267.0290, 239.0342, 223.0602, 207.0654, 164.0472, 149.0238, 101.0240 | 6'-*O*-Sinapoylgeniposide | GFP |
| 92 | 43.62 | C_17_H_14_O_7_ | [M-H]- | 329.0656 | -1.82 | 329.0656, 314.0422, 299.0187, 285.0386, 271.0239, 255.0291, 243.0290, 227.0342, 215.0340, 183.0444, 155.0496, 143.0497, 124.9876 | Trihydroxy-dimethoxy-flavone | WSR |
| 93 | 44.13 | C_16_H_12_O_6_ | [M-H]- | 299.055 | -2.01 | 299.0549, 284.0313, 267.0285, 239.0343, 212.0469, 181.9848, 165.9900, 137.9953, 110.0004 | Trihydroxymethoxy-flavone or isomer | WSR |
| 94 | 44.76 | C_17_H_14_O_7_ | [M-H]- | 329.0657 | -1.52 | 329.0659, 314.0423, 299.0190, 227.2954, 220.2846, 156.8901, 135.0450 | Trihydroxy-dimethoxy-flavone or isomer | WSR |
| 95 | 45.38 | C_32_H_44_O_14_ | [M-H]- | 651.2638 | -2.30 | N/A | all-trans-Crocetin di-β-*D*-glucosyl eater | GFP |
| 96 | 46.08 | C_44_H_64_O_24_ | [M+HCOO]- | 975.367 | -4.00 | N/A | Crocin I isomer | GFP |
| 97 | 46.28 | C_17_H_14_O_7_ | [M-H]- | 329.0656 | -1.82 | 329.2325, 314.0417, 299.0190, 293.2122, 243.0294, 229.1436, 211.1331, 199.0387, 183.1381, 171.1020, 139.1123, 127.1124 | Trihydroxy-dimethoxy-flavone or isomer | WSR |
| 98 | 47.40 | C_18_H_16_O_7_ | [M-H]- | 343.0806 | -3.50 | 343.0818, 328.0581, 313.0345, 285.0398, 269.2064, 227.0984, 180.0054, 164.9822, 136.9876, 116.2833 | Skullcap flavone | WSR |
| 99 | 47.47 | C_18_H_16_O_8_ | [M-H]- | 359.0758 | -2.51 | 359.0759, 344.0527, 329.0292, 301.0340, 231.0308, 202.0274, 165.0184, 115.3844 | 5,2 ',5 '-Trihydroxy-6,7, 8-trimethoxy-flavonoids | WSR |
| 100 | 47.5 | C_27_H_36_O_11_ | [M-H]- | 535.2167 | -2.24 | N/A | 6'-*O*-trans-sinapoyl jasminoside A or isomer | GFP |
| 101 | 47.65 | C_16_H_12_O_5_ | [M-H]- | 283.0598 | -3.18 | 283.0602, 268.0366, 239.0340, 198.0313, 184.0522, 163.0030, 139.0547, 110.0005 | Wogonin | WSR |
| 102 | 47.99 | C_15_H_10_O_4_ | [M-H]- | 253.0496 | -1.98 | 253.0496, 227.2941, 209.0601, 167.1526, 143.0498, 119.0497, 107.0136 | Chrysin | WSR |
| 103 | 48.28 | C_17_H_14_O_6_ | [M-H]- | 313.0705 | -2.24 | 313.0710, 298.0478, 283.0239, 260.1814, 255.0300, 236.7636, 211.0391, 183.0449, 164.9818, 142.8834l136.9877, 117.0337 | Dihydroxy-dimethoxy-flavone | WSR |
| 104 | 48.68 | C_19_H_18_O_8_ | [M-H]- | 373.0911 | -3.48 | 373.0918, 358.0684, 343.0448, 328.0215, 300.0266, 285.0031, 257.0083, 228.0420, 213.0186, 194.9928, 151.9745, 133.0291 | Skullcapflavone II | WSR |
| 105 | 48.8 | C_16_H_12_O_5_ | [M-H]- | 283.0601 | -2.12 | 283.0603, 268.0368, 239.0339, 211.0394, 184.0522, 163.0031, 137.9953, 110.0005 | Oroxylin A | WSR |
| 106 | 49.08 | C_30_H_18_O_10_ | [M-H]- | 313.0706 | -1.92 | 313.0710, 298.0478, 283.0239, 260.1814, 255.0300, 236.7636, 211.0391, 183.0449, 164.9818, 142.8834, 136.9877, 117.0337 | 8,8''-Bibaicalein | WSR |
| 107 | 49.13 | C_17_H_14_O_6_ | [M-H]- | 537.0808 | -2.61 | 537.0809, 391.0447, 373.0341, 335.0544, 270.9873, 245.0083, 217.0134, 189.0185, 145.0290, 133.0291 | Dihydroxy-dimethoxy-flavone | WSR |
| 108 | 50.48 | C_18_H_16_O_7_ | [M-H]- | 343.0806 | -3.50 | 343.0816, 328.0580, 313.0345, 298.0108, 270.0160, 242.0212, 198.0318, 179.9693, 151.9747, 123.9799 | Tenaxin I | WSR |
| 109 | 50.65 | C_48_H_60_O_22_ | [M-H]- | 987.3462 | -3.65 | N/A | Neocrocin B/C/D/E | GFP |
| 110 | 51.47 | C_32_H_44_O_14_ | [M-H]- | 651.2634 | -2.92 | N/A | Crocin-III or isomer 1 | GFP |
| 111 | 51.68 | C_32_H_44_O_14_ | [M-H]- | 651.2633 | -3.07 | N/A | Crocin-III or isomer 2 | GFP |
| 112 | 52.2 | C_32_H_44_O_14_ | [M-H]- | 651.2632 | -3.22 | N/A | Crocin-III or isomer 3 | GFP |
| 113 | 56.55 | C_20_H_24_O_4_ | [M-H]- | 327.1591 | -1.53 | 327.1598, 265.7544, 247.3086, 227.2941, 185.0083, 146.9381, 102.9485 | Crocetin | GFP |

## **Table S5** Method Validation of characteristic spectrum

| Peaks | Retention time (min) | Peaks iedntification | RSD of Peak area (%) | | |  | RSD of Retention time (%) | | |
| --- | --- | --- | --- | --- | --- | --- | --- | --- | --- |
|  |  |  | Precision | Repeatability | Stability (48h) |  | Precision | Repeatability | Stability (48h) |
| A1 | 4.962 | Geniposidic acid | 0.65 | 0.37 | 0.54 |  | 0.38 | 0.54 | 0.56 |
| A2 | 7.254 | 6α-hydroxygeniposide | 0.63 | 0.56 | 0.81 |  | 0.32 | 0.25 | 0.38 |
| A3 | 8.967 | Gardenoside | 1.51 | 1.34 | 0.83 |  | 0.27 | 0.51 | 0.37 |
| A4 | 10.061 | 6β-hydroxygeniposide | 2.66 | 4.83 | 2.74 |  | 0.51 | 0.37 | 0.77 |
| A5 | 11.823 | Picrocrocinic acid | 0.59 | 0.64 | 0.87 |  | 0.23 | 0.50 | 0.41 |
| A6 | 13.955 | Neochlorogenic acid | 1.39 | 1.95 | 2.00 |  | 0.13 | 0.36 | 0.24 |
| A7 | 14.597 | Chlorogenic acid | 1.36 | 0.32 | 2.01 |  | 0.12 | 0.31 | 0.23 |
| A8 | 14.894 | Protocatechuic acid | 1.95 | 1.32 | 1.17 |  | 0.05 | 0.16 | 0.15 |
| A9 | 16.241 | Genipin 1-gentiobioside | 0.57 | 3.20 | 0.95 |  | 0.03 | 0.02 | 0.09 |
| A10 | 16.765 | sinapyglucoside | 2.21 | 4.64 | 2.99 |  | 0.03 | 0.03 | 0.09 |
| A11 | 17.226 | Geniposide | 4.27 | 2.56 | 3.86 |  | 0.02 | 0.03 | 0.07 |
| A12 | 18.698 | Picrocrocin | 1.97 | 1.05 | 2.81 |  | 0.02 | 0.04 | 0.04 |
| A13 | 19.001 | Rutin-isomer | 4.57 | 2.96 | 1.70 |  | 0.02 | 0.04 | 0.05 |
| A14 | 21.686 | Carthamidin-7-*O*-glucuronide | 2.31 | 1.88 | 2.29 |  | 0.07 | 0.04 | 0.05 |
| A15 | 22.244 | Isoquercetin | 4.18 | 4.14 | 3.03 |  | 0.06 | 0.03 | 0.04 |
| A16 | 22.547 | Scutellarin | 1.31 | 3.24 | 3.15 |  | 0.04 | 0.03 | 0.05 |
| A17 | 22.833 | Chrysin 6-*C*-arabinoside 8-*C*-glucoside | 4.17 | 0.50 | 2.88 |  | 0.05 | 0.03 | 0.04 |
| A18 | 24.98 | Chrysin 6-*C*-glucoside 8-*C*-arabinoside | 2.05 | 1.34 | 1.05 |  | 0.03 | 0.02 | 0.04 |
| A19 | 25.814 | Chrysin 6-*C*-glucoside 8-*C*-arabinoside isomer | 4.07 | 3.56 | 2.36 |  | 0.03 | 0.02 | 0.04 |
| A20 | 25.978 | Trihydroxydihydrochalcone-3'-*C*-glucoside-6'-*O*-glucoside or isomer | 1.83 | 4.80 | 2.43 |  | 0.03 | 0.02 | 0.05 |
| A21 | 27.985 | viscidulin III | 2.42 | 3.84 | 3.83 |  | 0.02 | 0.78 | 0.03 |
| A22 | 29.152 | Trihydroxy-methoxy-flavone-7-*O*-glucuronide | 3.83 | 2.76 | 3.15 |  | 0.02 | 0.02 | 0.02 |
| A23 | 29.656 | 6''-*O*-[(*E*)-p-Coumaroyl] genipin gentiobioside or isomer | 2.44 | 1.44 | 2.44 |  | 0.04 | 0.02 | 0.04 |
| A24 | 31.704 | Baicalin | 0.00 | 0.00 | 0.00 |  | 0.00 | 0.00 | 0.00 |
| A25 | 33.149 | Dihydrobaicalin | 0.40 | 0.50 | 1.58 |  | 0.02 | 0.01 | 0.01 |
| A26 | 34.949 | Glychionide A | 0.08 | 0.18 | 0.52 |  | 0.02 | 0.01 | 0.02 |
| A27 | 35.572 | Oroxylin A 7-*O*-*D*-glucuronide isomer | 0.99 | 1.08 | 2.00 |  | 0.03 | 0.01 | 0.03 |
| A28 | 36.472 | Norwogonin-8-*O*-glucuronide | 0.62 | 0.80 | 0.78 |  | 0.02 | 0.02 | 0.05 |
| A29 | 37.135 | 5,7,2 '-Trihydroxy-6-methoxy-flavone-7-*O*-glucuronide | 0.70 | 1.31 | 1.18 |  | 0.02 | 0.02 | 0.06 |
| A30 | 37.771 | Chrysin-7-*O*-β-*D*-glucoronide | 0.31 | 1.07 | 1.34 |  | 0.03 | 0.02 | 0.07 |
| A31 | 38.968 | Oroxylin A-7-*O*-*D*-glucuronide | 0.11 | 0.31 | 0.74 |  | 0.03 | 0.02 | 0.09 |
| A32 | 40.261 | Kaempferol isomer | 0.25 | 0.34 | 0.67 |  | 0.04 | 0.02 | 0.08 |
| A33 | 41.775 | Baicalein-6-*O*-glucuronide | 1.04 | 0.65 | 1.08 |  | 0.04 | 0.02 | 0.09 |
| A34 | 42.455 | Wogonoside | 0.07 | 0.08 | 0.49 |  | 0.03 | 0.02 | 0.08 |
| A35 | 44.474 | Norwogonin | 1.25 | 0.84 | 2.46 |  | 0.02 | 0.02 | 0.04 |
| A36 | 45.96 | Trihydroxymethoxy-flavone | 1.28 | 4.56 | 4.14 |  | 0.03 | 0.02 | 0.06 |
| A37 | 46.389 | Baicalein | 2.12 | 4.18 | 3.47 |  | 0.03 | 0.02 | 0.07 |
| A38 | 50.852 | Wogonin | 1.34 | 2.51 | 1.05 |  | 0.03 | 0.03 | 0.09 |
| A39 | 51.603 | Chrysin | 2.15 | 3.48 | 2.21 |  | 0.02 | 0.04 | 0.08 |
| A40 | 51.985 | Oroxylin A | 1.72 | 4.10 | 0.78 |  | 0.03 | 0.03 | 0.09 |
| B1 | 27.031 | Unknown | 4.13 | 2.80 | 3.80 |  | 0.02 | 0.02 | 0.05 |
| B2 | 28.512 | Unknown | 4.09 | 1.77 | 3.51 |  | 0.02 | 0.03 | 0.03 |
| B3 | 28.94 | Unknown | 2.49 | 2.83 | 2.24 |  | 0.02 | 0.03 | 0.03 |
| B4 | 29.244 | Unknown | 4.43 | 4.00 | 2.90 |  | 0.01 | 0.02 | 0.04 |
| B5 | 30.151 | Unknown | 3.07 | 2.70 | 4.46 |  | 0.02 | 0.01 | 0.02 |
| B6 | 32.792 | Crocin I | 0.00 | 0.00 | 0.00 |  | 0.00 | 0.00 | 0.00 |
| B7 | 34.101 | Unknown | 1.74 | 0.58 | 2.17 |  | 0.01 | 0.01 | 0.03 |
| B8 | 39.524 | Crocin II | 0.65 | 2.13 | 1.11 |  | 0.04 | 0.02 | 0.13 |
| B9 | 46.324 | Unknown | 0.60 | 4.20 | 0.84 |  | 0.02 | 0.02 | 0.09 |
| B10 | 46.975 | Unknown | 1.45 | 1.75 | 2.25 |  | 0.02 | 0.02 | 0.07 |
| B11 | 48.756 | all-trans-Crocetin di-β-*D*-glucosyl eater | 0.98 | 1.91 | 0.69 |  | 0.02 | 0.03 | 0.08 |
| B12 | 49.487 | Crocin I isomer | 0.09 | 2.79 | 0.40 |  | 0.02 | 0.04 | 0.08 |
| B13 | 50.215 | Neocrocin B/C/D/E | 4.57 | 3.85 | 3.54 |  | 0.03 | 0.03 | 0.06 |
| B14 | 54.913 | Crocin-III or isomer | 1.17 | 3.12 | 1.65 |  | 0.03 | 0.03 | 0.05 |
| B15 | 55.102 | Crocin-III or isomer | 2.97 | 4.45 | 1.86 |  | 0.03 | 0.03 | 0.05 |
| B16 | 55.627 | Crocin-III or isomer | 1.46 | 1.84 | 1.36 |  | 0.03 | 0.03 | 0.05 |
| B17 | 59.06 | Crocetin | 3.72 | 3.83 | 3.96 |  | 0.04 | 0.02 | 0.05 |

## **Table S6** The difference compounds between PW1-PW2-PW3-PW4

| Peak No. | Compound Name | VIP value |
| --- | --- | --- |
| A2 | 6α-hydroxygeniposide | 1.0872 |
| A3 | Gardenoside | 1.0719 |
| A9 | Genipin 1-gentiobioside | 1.3822 |
| A12 | Picrocrocin | 1.0936 |
| A13 | Rutin-isomer | 1.2123 |
| A19 | Chrysin 6-*C*-glucoside 8-*C*-arabinoside isomer | 1.0366 |
| A20 | Trihydroxydihydrochalcone-3'-*C*-glucoside-6'-*O*-glucoside or isomer | 1.1356 |
| A23 | 6''-*O*-[(*E*)-p-Coumaroyl] genipin gentiobioside or isomer | 1.0579 |
| A24 | Baicalin | 1.1275 |
| A26 | Glychionide A | 1.2167 |
| A27 | Oroxylin A 7-*O*-*D*-glucuronide isomer | 1.1747 |
| A30 | Chrysin-7-*O*-β-*D*-glucoronide | 1.2196 |
| A34 | Wogonoside | 1.0803 |
| A39 | Chrysin | 1.2227 |
| A40 | Oroxylin A | 1.2454 |
| B11 | all-trans-Crocetin di-β-*D*-glucosyl eater | 1.0747 |

## **Table S7** The difference compounds between PW1-PW2.

| Peak No. | Compound Name | VIP value |
| --- | --- | --- |
| A1 | Geniposidic acid | 1.2708 |
| A3 | Gardenoside | 1.0014 |
| A4 | 6β-hydroxygeniposide | 1.2433 |
| A5 | Picrocrocinic acid | 1.2412 |
| A6 | Neochlorogenic acid | 1.1868 |
| A7 | Chlorogenic acid | 1.0022 |
| A8 | Protocatechuic acid | 1.2704 |
| A9 | Genipin 1-gentiobioside | 1.0776 |
| A10 | sinapyglucoside | 1.1309 |
| A14 | Carthamidin-7-*O*-glucuronide | 1.2054 |
| A15 | Isoquercetin | 1.0814 |
| A23 | 6''-*O*-[(*E*)-p-Coumaroyl] genipin gentiobioside or isomer | 1.2538 |
| A26 | Glychionide A | 1.1354 |
| A27 | Oroxylin A 7-*O*-*D*-glucuronide isomer | 1.0147 |
| A34 | Wogonoside | 1.0091 |
| A39 | Chrysin | 1.1178 |
| B1 | Unknown | 1.2716 |
| B2 | Unknown | 1.2711 |
| B3 | Unknown | 1.2652 |
| B4 | Unknown | 1.2682 |
| B5 | Unknown | 1.2714 |
| B6 | Crocin I | 1.2707 |
| B7 | Unknown | 1.2690 |
| B8 | Crocin II | 1.2693 |
| B9 | Unknown | 1.2694 |
| B10 | Unknown | 1.2632 |
| B12 | Crocin I isomer | 1.2714 |
| B13 | Neocrocin B/C/D/E | 1.2711 |
| B15 | Crocin-III or isomer | 1.2678 |
| B17 | Crocetin | 1.2125 |

## **Table S8** The difference compounds between PW1-PW3

| Peak No. | Compound Name | VIP value |
| --- | --- | --- |
| A3 | Gardenoside | 1.2224 |
| A8 | Protocatechuic acid | 1.1895 |
| A9 | Genipin 1-gentiobioside | 1.3205 |
| A10 | sinapyglucoside | 1.1301 |
| A11 | Geniposide | 1.0724 |
| A15 | Isoquercetin | 1.1685 |
| A16 | Scutellarin | 1.0919 |
| A18 | Chrysin 6-*C*-glucoside 8-*C*-arabinoside | 1.0000 |
| A19 | Chrysin 6-*C*-glucoside 8-*C*-arabinoside isomer | 1.1433 |
| A20 | Trihydroxydihydrochalcone-3'-*C*-glucoside-6'-*O*-glucoside or isomer | 1.2028 |
| A27 | Oroxylin A 7-*O*-*D*-glucuronide isomer | 1.1051 |
| A32 | Kaempferol isomer | 1.0829 |
| A40 | Oroxylin A | 1.1922 |
| B1 | Unknown | 1.3456 |
| B2 | Unknown | 1.3732 |
| B3 | Unknown | 1.1400 |
| B4 | Unknown | 1.2808 |
| B5 | Unknown | 1.3391 |
| B6 | Crocin I | 1.3264 |
| B8 | Crocin II | 1.3405 |
| B9 | Unknown | 1.2494 |
| B10 | Unknown | 1.1708 |
| B11 | all-trans-Crocetin di-β-*D*-glucosyl eater | 1.1708 |
| B12 | Crocin I isomer | 1.3122 |
| B14 | Crocin-III or isomer | 1.0609 |
| B15 | Crocin-III or isomer | 1.0437 |
| B16 | Crocin-III or isomer | 1.0442 |

## **Table S9** The difference compounds between PW2-PW4

| Peak No. | Compound Name | VIP value |
| --- | --- | --- |
| A1 | Geniposidic acid | 1.0938 |
| A2 | 6α-hydroxygeniposide | 1.2416 |
| A3 | Gardenoside | 1.0153 |
| A8 | Protocatechuic acid | 1.0871 |
| A12 | Picrocrocin | 1.3055 |
| A13 | Rutin-isomer | 1.4153 |
| A14 | Carthamidin-7-*O*-glucuronide | 1.1117 |
| A17 | Chrysin 6-*C*-arabinoside 8-*C*-glucoside | 1.0162 |
| A23 | 6''-*O*-[(*E*)-p-Coumaroyl] genipin gentiobioside or isomer | 1.2887 |
| A24 | Baicalin | 1.3013 |
| A26 | Glychionide A | 1.3717 |
| A27 | Oroxylin A 7-*O*-*D*-glucuronide isomer | 1.1906 |
| A30 | Chrysin-7-O-β-*D*-glucoronide | 1.5061 |
| A31 | Oroxylin A-7-*O*-*D*-glucuronide | 1.1174 |
| A34 | Wogonoside | 1.2146 |
| A36 | Trihydroxymethoxy-flavone | 1.1816 |
| A39 | Chrysin | 1.4085 |

## **Table S10** The difference compounds between PW1-PW4

| Peak No. | Compound Name | VIP value |
| --- | --- | --- |
| A1 | Geniposidic acid | 1.2438 |
| A2 | 6α-hydroxygeniposide | 1.1623 |
| A4 | 6β-hydroxygeniposide | 1.0678 |
| A5 | Picrocrocinic acid | 1.2123 |
| A8 | Protocatechuic acid | 1.2037 |
| A10 | sinapyglucoside | 1.1148 |
| A11 | Geniposide | 1.1051 |
| A12 | Picrocrocin | 1.1815 |
| A13 | Rutin-isomer | 1.1485 |
| A14 | Carthamidin-7-*O*-glucuronide | 1.1697 |
| A15 | Isoquercetin | 1.1634 |
| A16 | Scutellarin | 1.0627 |
| A22 | Trihydroxy-methoxy-flavone-7-*O*-glucuronide | 1.0287 |
| A23 | 6''-*O*-[(*E*)-*p*-Coumaroyl] genipin gentiobioside or isomer | 1.1140 |
| A27 | Oroxylin A 7-*O*-*D*-glucuronide isomer | 1.1239 |
| A30 | Chrysin-7-*O*-β-*D*-glucoronide | 1.0165 |
| B1 | Unknown | 1.2449 |
| B2 | Unknown | 1.2445 |
| B3 | Unknown | 1.2386 |
| B4 | Unknown | 1.2411 |
| B5 | Unknown | 1.2445 |
| B6 | Crocin I | 1.2436 |
| B7 | Unknown | 1.2428 |
| B8 | Crocin II | 1.2424 |
| B9 | Unknown | 1.2424 |
| B10 | Unknown | 1.2362 |
| B12 | Crocin I isomer | 1.2446 |
| B13 | Neocrocin B/C/D/E | 1.2445 |
| B14 | Crocin-III or isomer | 1.0098 |
| B15 | Crocin-III or isomer | 1.2406 |
| B16 | Crocin-III or isomer | 1.0273 |
| B17 | Crocetin | 1.2271 |

## **Table S11** The difference compounds between PW2-PW3

| Peak No. | Compound Name | VIP value |
| --- | --- | --- |
| A1 | Geniposidic acid | 1.2183 |
| A4 | 6β-hydroxygeniposide | 1.1289 |
| A5 | Picrocrocinic acid | 1.2080 |
| A6 | Neochlorogenic acid | 1.1572 |
| A8 | Protocatechuic acid | 1.2124 |
| A10 | sinapyglucoside | 1.1803 |
| A14 | Carthamidin-7-*O*-glucuronide | 1.1641 |
| A17 | Chrysin 6-*C*-arabinoside 8-*C*-glucoside | 1.0040 |
| A18 | Chrysin 6-*C*-glucoside 8-*C*-arabinoside | 1.2023 |
| A19 | Chrysin 6-*C*-glucoside 8-*C*-arabinoside isomer | 1.1208 |
| A23 | 6''-*O*-[(*E*)-*p*-Coumaroyl] genipin gentiobioside or isomer | 1.2052 |
| A26 | Glychionide A | 1.0353 |
| B1 | Unknown | 1.2187 |
| B2 | Unknown | 1.2201 |
| B3 | Unknown | 1.2134 |
| B4 | Unknown | 1.2060 |
| B5 | Unknown | 1.2185 |
| B6 | Crocin I | 1.2170 |
| B7 | Unknown | 1.2098 |
| B8 | Crocin II | 1.2178 |
| B9 | Unknown | 1.2049 |
| B10 | Unknown | 1.2006 |
| B11 | all-trans-Crocetin di-β-*D*-glucosyl eater | 1.0782 |
| B12 | Crocin I isomer | 1.2173 |
| B13 | Neocrocin B/C/D/E | 1.2143 |
| B15 | Crocin-III or isomer | 1.1872 |
| B17 | Crocetin | 1.1634 |

## **Table S12** The difference compounds between PW3-PW4

| Peak No. | Compound Name | VIP value |
| --- | --- | --- |
| A1 | Geniposidic acid | 1.2274 |
| A2 | 6α-hydroxygeniposide | 1.0568 |
| A4 | 6β-hydroxygeniposide | 1.0433 |
| A5 | Picrocrocinic acid | 1.2157 |
| A8 | Protocatechuic acid | 1.1942 |
| A10 | sinapyglucoside | 1.1801 |
| A12 | Picrocrocin | 1.1701 |
| A13 | Rutin-isomer | 1.1360 |
| A14 | Carthamidin-7-*O*-glucuronide | 1.1504 |
| A15 | Isoquercetin | 1.0791 |
| A23 | 6''-*O*-[(*E*)-*p*-Coumaroyl] genipin gentiobioside or isomer | 1.1320 |
| B1 | Unknown | 1.2279 |
| B2 | Unknown | 1.2291 |
| B3 | Unknown | 1.2235 |
| B4 | Unknown | 1.2182 |
| B5 | Unknown | 1.2268 |
| B6 | Crocin I | 1.2268 |
| B7 | Unknown | 1.2194 |
| B8 | Crocin II | 1.2265 |
| B9 | Unknown | 1.2152 |
| B10 | Unknown | 1.2156 |
| B12 | Crocin I isomer | 1.2267 |
| B13 | Neocrocin B/C/D/E | 1.2248 |
| B15 | Crocin-III or isomer | 1.1900 |
| B17 | Crocetin | 1.2107 |

## **Table S13** Result of the pharmacodynamic study

| Item | Lung injury score (n=3) | W/D ratio of Lung (n=6) | TNF-α (pg/mL, n=6) | IL-6 (pg/mL, n=6) | IL-1β (pg/mL, n=6) | Protein in BALF (mg/mL, n=6) | Neutrophils in BALF (10^9/L, n=6) |
| --- | --- | --- | --- | --- | --- | --- | --- |
| Con | 0.01±0.00 | 4.06±0.06 | 60.28±16.78 | 517.50±78.05 | 197.92±6.65 | 0.25±0.04 | 1.98±0.52 |
| LPS | 0.86±0.05 | 4.92±0.07 | 395.72±155.90 | 875.42±132.35 | 1565.25±323.98 | 0.71±0.11 | 26.28±4.53 |
| LPS_DEX | 0.21±0.03 | 4.33±0.08 | 143.61±89.66 | 560.42±37.31 | 239.17±73.59 | 0.32±0.07 | 1.61±0.73 |
| LPS_LPW1 | 0.63±0.01 | 4.74±0.04 | 258.06±66.79 | 816.67±109.29 | 589.75±323.76 | 0.54±0.09 | 16.32±13.72 |
| LPS_PW1 | 0.55±0.08 | 4.68±0.07 | 138.34±81.49 | 637.92±29.90 | 437.50±209.93 | 0.45±0.02 | 9.29±2.53 |
| LPS_PW2 | 0.47±0.06 | 4.59±0.08 | 78.67±29.76 | 528.25±63.80 | 480.00±194.21 | 0.48±0.03 | 13.22±9.89 |
| LPS_PW3 | 0.40±0.07 | 4.59±0.06 | 174.45±57.01 | 559.17±29.34 | 757.92±178.33 | 0.50±0.08 | 8.23±4.82 |
| LPS_PW4 | 0.42±0.02 | 4.51±0.13 | 166.11±76.17 | 480.00±68.73 | 650.75±315.56 | 0.47±0.03 | 6.57±3.58 |

## **Table S14** Judgment matrix of AHP assessment factors

| Inedx | W/D ratio of Lung | Lung injury score | Neutrophils in BALF | TNF-α | IL-6 | IL-1β | Protein in BALF |
| --- | --- | --- | --- | --- | --- | --- | --- |
| W/D ratio of Lung | 1 | 0.2 | 0.333 | 0.5 | 0.5 | 0.5 | 1 |
| Lung injury score | 5 | 1 | 1.667 | 2.5 | 2.5 | 2.5 | 5 |
| Neutrophils in BALF | 3 | 0.6 | 1 | 1.5 | 1.5 | 1.5 | 3 |
| TNF-α | 2 | 0.4 | 0.667 | 1 | 1 | 1 | 2 |
| IL-6 | 2 | 0.4 | 0.667 | 1 | 1 | 1 | 2 |
| IL-1β | 2 | 0.4 | 0.667 | 1 | 1 | 1 | 2 |
| Protein in BALF | 1 | 0.2 | 0.333 | 0.5 | 0.5 | 0.5 | 1 |

## **Table S15** Result of AHP analysis

| Inedx | *W_i_* | *w_j_* | *λ_max_* | *CI* | *RI* | *CR* |
| --- | --- | --- | --- | --- | --- | --- |
| W/D ratio of Lung | 0.438 | 6.25% | 7 | 0 | 1.36 | 0 |
| Lung injury score | 2.188 | 31.25% |  |  |  |  |
| Neutrophils in BALF | 1.313 | 18.75% |  |  |  |  |
| TNF-α | 0.875 | 12.50% |  |  |  |  |
| IL-6 | 0.875 | 12.50% |  |  |  |  |
| IL-1β | 0.875 | 12.50% |  |  |  |  |
| Protein in BALF | 0.438 | 6.25% |  |  |  |  |

## **Table S16** Result of EWM analysis

| Inedx | e*_j_* | d*_j_* | *v*_j_ |
| --- | --- | --- | --- |
| W/D ratio of Lung | 0.9876 | 0.0124 | 22.47% |
| Lung injury score | 0.9922 | 0.0078 | 14.19% |
| Neutrophils in BALF | 0.9922 | 0.0078 | 14.17% |
| TNF-α | 0.9925 | 0.0075 | 13.61% |
| IL-6 | 0.9883 | 0.0117 | 21.22% |
| IL-1β | 0.9942 | 0.0058 | 10.47% |
| protein in BALF | 0.9979 | 0.0021 | 3.86% |

## **Table S17** Combined weight results for each index

| Inedx | *v*_j_ | *w*_j_ | *w*_j_*v*_j_ | ∑*w*_j_*v*_j_ | *T_j_* |
| --- | --- | --- | --- | --- | --- |
| W/D ratio of Lung | 22.47% | 6.25% | 0.0140 | 0.1440 | 0.0975 |
| Lung injury score | 14.19% | 31.25% | 0.0443 |  | 0.3080 |
| Neutrophils in BALF | 14.17% | 18.75% | 0.0266 |  | 0.1845 |
| TNF-α | 13.61% | 12.50% | 0.0170 |  | 0.1181 |
| IL-6 | 21.22% | 12.50% | 0.0265 |  | 0.1842 |
| IL-1β | 10.47% | 12.50% | 0.0131 |  | 0.0909 |
| protein in BALF | 3.86% | 6.25% | 0.0024 |  | 0.0168 |

## **Table S18** Pearson analysis results of characteristic peaks and each pharmacodynamic index of HQQFD

| **No.** | **Lung injury score** | |  | **TNF-α** | |  | **IL-6** | |  | **IL-1β** | |  | **W/D ratio of Lung** | |  | **Protein in BALF** | |  | **Neutrophils in BALF** | |  | **Overall rating** | |
| --- | --- | --- | --- | --- | --- | --- | --- | --- | --- | --- | --- | --- | --- | --- | --- | --- | --- | --- | --- | --- | --- | --- | --- |
|  | **Peaks** | **Correlation coefficien** |  | **Peaks** | **Correlation coefficien** |  | **Peaks** | **Correlation coefficien** |  | **Peaks** | **Correlation coefficien** |  | **Peaks** | **Correlation coefficien** |  | **Peaks** | **Correlation coefficien** |  | **Peaks** | **Correlation coefficien** |  | **Peaks** | **Correlation coefficien** |
| 1 | A40 | 0.928 |  | A7 | 0.944 |  | A27 | 0.974 |  | A20 | 0.984 |  | B16 | 0.998 |  | A9 | 0.967 |  | A24 | 0.994 |  | A11 | 0.994 |
| 2 | A27 | 0.876 |  | A32 | 0.924 |  | B16 | 0.974 |  | A18 | 0.965 |  | B14 | 0.992 |  | A20 | 0.877 |  | A26 | 0.967 |  | B14 | 0.990 |
| 3 | A11 | 0.830 |  | A31 | 0.847 |  | B14 | 0.948 |  | A19 | 0.839 |  | A11 | 0.982 |  | A19 | 0.804 |  | A30 | 0.959 |  | B16 | 0.986 |
| 4 | B14 | 0.781 |  | A36 | 0.844 |  | A11 | 0.919 |  | A32 | 0.78 |  | A27 | 0.974 |  | A18 | 0.752 |  | A34 | 0.928 |  | A27 | 0.968 |
| 5 | B16 | 0.774 |  | A2 | 0.821 |  | A1 | 0.830 |  | A9 | 0.685 |  | A1 | 0.720 |  | A32 | 0.463 |  | A17 | 0.695 |  | A30 | 0.659 |
| 6 | B11 | 0.618 |  | A18 | 0.778 |  | B17 | 0.814 |  | A16 | 0.661 |  | B17 | 0.691 |  | A34 | 0.384 |  | B11 | 0.598 |  | A1 | 0.630 |
| 7 | A30 | 0.500 |  | A39 | 0.763 |  | A6 | 0.734 |  | A36 | 0.642 |  | A30 | 0.603 |  | A16 | 0.319 |  | A11 | 0.536 |  | A40 | 0.613 |
| 8 | A17 | 0.480 |  | A6 | 0.630 |  | A40 | 0.482 |  | A2 | 0.572 |  | A6 | 0.580 |  | A26 | 0.271 |  | A23 | 0.530 |  | B17 | 0.598 |
| 9 | A24 | 0.276 |  | A20 | 0.622 |  | A30 | 0.428 |  | A7 | 0.457 |  | A40 | 0.543 |  | A15 | 0.212 |  | B14 | 0.475 |  | A6 | 0.480 |
| 10 | A1 | 0.262 |  | A19 | 0.607 |  | A26 | 0.147 |  | A13 | 0.271 |  | A26 | 0.331 |  | A24 | 0.122 |  | B16 | 0.386 |  | A26 | 0.387 |
| 11 | B17 | 0.249 |  | B17 | 0.480 |  | A34 | 0.119 |  | A15 | 0.229 |  | A34 | 0.292 |  | A36 | 0.120 |  | B4 | 0.266 |  | A24 | 0.349 |
| 12 | A26 | 0.199 |  | A16 | 0.472 |  | A31 | 0.103 |  | A31 | 0.188 |  | A24 | 0.270 |  | A22 | 0.077 |  | B9 | 0.263 |  | A34 | 0.336 |
| 13 | A6 | 0.179 |  | A13 | 0.441 |  | A24 | 0.066 |  | A6 | 0.139 |  | B11 | 0.134 |  | A23 | 0.053 |  | B2 | 0.261 |  | B11 | 0.251 |
| 14 | A3 | 0.163 |  | A1 | 0.422 |  | A32 | 0.044 |  | A12 | 0.098 |  | A17 | 0.034 |  | A14 | 0.039 |  | B8 | 0.261 |  | A17 | 0.155 |
| 15 | A34 | 0.098 |  | A3 | 0.360 |  | A39 | 0.039 |  | A14 | 0.095 |  | A31 | -0.089 |  | A2 | 0.034 |  | B10 | 0.261 |  | A31 | -0.153 |
| 16 | A39 | 0.036 |  | A12 | 0.161 |  | A19 | 0.007 |  | A39 | 0.093 |  | A19 | -0.111 |  | A1 | 0.019 |  | B12 | 0.259 |  | A39 | -0.182 |
| 17 | A10 | 0.035 |  | A9 | 0.022 |  | B11 | -0.032 |  | A22 | 0.084 |  | A39 | -0.135 |  | B17 | -0.006 |  | B5 | 0.258 |  | A19 | -0.218 |
| 18 | B4 | 0.013 |  | A27 | -0.150 |  | A7 | -0.095 |  | B17 | 0.063 |  | A32 | -0.149 |  | A30 | -0.007 |  | B6 | 0.257 |  | A9 | -0.250 |
| 19 | B9 | -0.013 |  | A4 | -0.192 |  | A17 | -0.154 |  | A1 | 0.045 |  | A9 | -0.203 |  | B13 | -0.035 |  | B1 | 0.256 |  | A32 | -0.268 |
| 20 | A31 | -0.018 |  | A15 | -0.239 |  | A3 | -0.209 |  | A4 | 0.036 |  | A3 | -0.294 |  | B7 | -0.040 |  | B15 | 0.256 |  | A3 | -0.275 |
| 21 | B2 | -0.031 |  | B16 | -0.243 |  | A9 | -0.219 |  | B13 | -0.084 |  | A7 | -0.299 |  | A6 | -0.042 |  | B3 | 0.248 |  | A7 | -0.377 |
| 22 | B10 | -0.031 |  | A14 | -0.259 |  | A18 | -0.291 |  | B7 | -0.09 |  | A18 | -0.440 |  | A4 | -0.093 |  | B7 | 0.238 |  | A23 | -0.409 |
| 23 | B8 | -0.034 |  | A22 | -0.323 |  | A20 | -0.381 |  | A8 | -0.102 |  | A20 | -0.492 |  | A7 | -0.103 |  | B13 | 0.236 |  | B4 | -0.421 |
| 24 | B5 | -0.069 |  | B14 | -0.334 |  | A2 | -0.491 |  | A5 | -0.159 |  | A23 | -0.511 |  | B3 | -0.121 |  | A40 | 0.231 |  | A10 | -0.430 |
| 25 | B6 | -0.078 |  | A8 | -0.350 |  | A36 | -0.505 |  | B3 | -0.165 |  | B4 | -0.526 |  | A5 | -0.127 |  | A5 | 0.230 |  | B9 | -0.441 |
| 26 | B1 | -0.083 |  | A40 | -0.410 |  | A10 | -0.638 |  | A23 | -0.181 |  | A10 | -0.531 |  | A8 | -0.154 |  | A9 | 0.226 |  | B2 | -0.455 |
| 27 | B15 | -0.085 |  | A11 | -0.422 |  | B4 | -0.652 |  | A3 | -0.194 |  | B9 | -0.545 |  | B12 | -0.154 |  | A27 | 0.221 |  | B10 | -0.455 |
| 28 | B12 | -0.109 |  | A10 | -0.451 |  | B9 | -0.671 |  | A34 | -0.195 |  | B2 | -0.559 |  | B15 | -0.181 |  | A10 | 0.167 |  | B8 | -0.457 |
| 29 | A23 | -0.136 |  | B13 | -0.465 |  | A23 | -0.680 |  | B12 | -0.199 |  | B10 | -0.559 |  | B1 | -0.184 |  | A22 | 0.107 |  | B5 | -0.483 |
| 30 | B3 | -0.144 |  | B7 | -0.468 |  | A13 | -0.681 |  | B15 | -0.221 |  | B8 | -0.560 |  | A13 | -0.188 |  | A8 | 0.086 |  | B6 | -0.490 |
| 31 | A5 | -0.148 |  | A5 | -0.476 |  | B2 | -0.684 |  | B1 | -0.223 |  | B5 | -0.585 |  | B6 | -0.188 |  | A15 | 0.051 |  | B1 | -0.494 |
| 32 | A8 | -0.190 |  | B3 | -0.492 |  | B10 | -0.684 |  | B6 | -0.228 |  | B6 | -0.591 |  | B5 | -0.198 |  | A14 | 0.029 |  | B15 | -0.495 |
| 33 | B7 | -0.221 |  | B12 | -0.507 |  | B8 | -0.685 |  | B5 | -0.237 |  | B1 | -0.595 |  | B8 | -0.233 |  | A4 | -0.067 |  | B12 | -0.511 |
| 34 | B13 | -0.226 |  | B15 | -0.508 |  | B5 | -0.709 |  | B8 | -0.269 |  | B15 | -0.597 |  | B2 | -0.236 |  | A1 | -0.192 |  | B3 | -0.538 |
| 35 | A12 | -0.283 |  | B1 | -0.509 |  | B6 | -0.716 |  | B2 | -0.272 |  | B12 | -0.611 |  | B10 | -0.236 |  | B17 | -0.262 |  | A18 | -0.543 |
| 36 | A7 | -0.302 |  | B6 | -0.511 |  | B1 | -0.719 |  | B10 | -0.272 |  | B3 | -0.636 |  | A12 | -0.249 |  | A19 | -0.347 |  | A5 | -0.546 |
| 37 | A4 | -0.309 |  | B5 | -0.513 |  | B15 | -0.721 |  | B9 | -0.289 |  | A5 | -0.643 |  | B9 | -0.254 |  | A12 | -0.439 |  | A20 | -0.582 |
| 38 | A13 | -0.378 |  | B8 | -0.521 |  | B12 | -0.735 |  | A26 | -0.31 |  | A2 | -0.657 |  | B4 | -0.280 |  | A20 | -0.442 |  | B7 | -0.590 |
| 39 | A22 | -0.382 |  | B2 | -0.522 |  | A12 | -0.748 |  | B4 | -0.312 |  | A36 | -0.674 |  | B14 | -0.348 |  | A6 | -0.443 |  | B13 | -0.594 |
| 40 | A14 | -0.384 |  | B10 | -0.522 |  | B3 | -0.758 |  | A10 | -0.32 |  | B7 | -0.684 |  | A10 | -0.353 |  | A16 | -0.605 |  | A8 | -0.614 |
| 41 | A15 | -0.514 |  | B9 | -0.526 |  | A5 | -0.762 |  | A24 | -0.438 |  | B13 | -0.687 |  | B16 | -0.365 |  | A18 | -0.613 |  | A2 | -0.709 |
| 42 | A2 | -0.537 |  | B4 | -0.531 |  | A8 | -0.795 |  | A30 | -0.556 |  | A8 | -0.703 |  | A31 | -0.371 |  | A3 | -0.627 |  | A4 | -0.724 |
| 43 | A32 | -0.551 |  | A23 | -0.699 |  | B7 | -0.803 |  | B16 | -0.589 |  | A13 | -0.764 |  | A11 | -0.405 |  | A13 | -0.680 |  | A22 | -0.725 |
| 44 | A36 | -0.600 |  | A34 | -0.779 |  | B13 | -0.806 |  | B14 | -0.62 |  | A12 | -0.770 |  | A17 | -0.457 |  | A32 | -0.760 |  | A12 | -0.727 |
| 45 | A19 | -0.659 |  | B11 | -0.812 |  | A4 | -0.858 |  | A27 | -0.689 |  | A4 | -0.798 |  | A39 | -0.472 |  | A39 | -0.898 |  | A36 | -0.732 |
| 46 | A9 | -0.671 |  | A26 | -0.847 |  | A16 | -0.887 |  | A11 | -0.698 |  | A22 | -0.803 |  | A27 | -0.596 |  | A31 | -0.932 |  | A14 | -0.748 |
| 47 | A16 | -0.781 |  | A30 | -0.852 |  | A22 | -0.896 |  | A17 | -0.73 |  | A14 | -0.823 |  | B11 | -0.634 |  | A36 | -0.936 |  | A13 | -0.755 |
| 48 | A18 | -0.835 |  | A17 | -0.886 |  | A14 | -0.901 |  | B11 | -0.833 |  | A15 | -0.872 |  | A3 | -0.674 |  | A2 | -0.937 |  | A15 | -0.807 |
| 49 | A20 | -0.899 |  | A24 | -0.943 |  | A15 | -0.950 |  | A40 | -0.95 |  | A16 | -0.963 |  | A40 | -0.963 |  | A7 | -0.996 |  | A16 | -0.976 |

## **Table S19** Gray relational analysis results of characteristic peaks and each pharmacodynamic index of HQQFD

| **No.** | **Lung injury score** | |  | **TNF-α** | |  | **IL-6** | |  | **IL-1β** | |  | **W/D ratio of Lung** | |  | **protein in BALF** | |  | **Neutrophils in BALF** | |  | **Overall rating** | |
| --- | --- | --- | --- | --- | --- | --- | --- | --- | --- | --- | --- | --- | --- | --- | --- | --- | --- | --- | --- | --- | --- | --- | --- |
|  | **Peaks** | **Correlation degree** |  | **Peaks** | **Correlation degree** |  | **Peaks** | **Correlation degree** |  | **Peaks** | **Correlation degree** |  | **Peaks** | **Correlation degree** |  | **Peaks** | **Correlation degree** |  | **Peaks** | **Correlation degree** |  | **Peaks** | **Correlation degree** |
| 1 | B14 | 0.886 |  | A19 | 0.936 |  | A27 | 0.865 |  | A19 | 0.906 |  | A27 | 0.966 |  | A19 | 0.962 |  | A24 | 0.875 |  | A27 | 0.962 |
| 2 | A27 | 0.882 |  | A20 | 0.934 |  | A40 | 0.864 |  | A20 | 0.904 |  | B16 | 0.945 |  | A20 | 0.958 |  | A23 | 0.874 |  | A40 | 0.959 |
| 3 | B16 | 0.880 |  | A9 | 0.934 |  | B16 | 0.861 |  | A18 | 0.904 |  | A11 | 0.941 |  | A18 | 0.958 |  | A26 | 0.874 |  | B16 | 0.959 |
| 4 | A11 | 0.879 |  | A18 | 0.934 |  | A11 | 0.861 |  | A32 | 0.904 |  | B17 | 0.919 |  | A32 | 0.957 |  | A34 | 0.873 |  | A11 | 0.958 |
| 5 | A40 | 0.879 |  | A32 | 0.933 |  | A6 | 0.856 |  | A9 | 0.903 |  | A1 | 0.917 |  | A9 | 0.956 |  | A17 | 0.873 |  | B17 | 0.954 |
| 6 | B17 | 0.877 |  | A1 | 0.930 |  | B17 | 0.856 |  | B14 | 0.902 |  | A6 | 0.907 |  | A1 | 0.951 |  | A30 | 0.872 |  | A6 | 0.954 |
| 7 | A1 | 0.877 |  | B17 | 0.930 |  | A1 | 0.856 |  | A1 | 0.902 |  | A39 | 0.904 |  | B14 | 0.951 |  | B7 | 0.871 |  | A1 | 0.954 |
| 8 | A6 | 0.877 |  | A6 | 0.930 |  | A39 | 0.855 |  | B17 | 0.901 |  | A40 | 0.898 |  | B17 | 0.951 |  | B13 | 0.871 |  | A39 | 0.951 |
| 9 | A31 | 0.872 |  | A34 | 0.926 |  | A31 | 0.854 |  | A6 | 0.901 |  | A3 | 0.897 |  | A6 | 0.951 |  | B11 | 0.871 |  | A31 | 0.951 |
| 10 | A39 | 0.872 |  | A7 | 0.924 |  | A3 | 0.854 |  | A7 | 0.895 |  | A31 | 0.894 |  | A7 | 0.941 |  | B12 | 0.871 |  | A3 | 0.950 |
| 11 | A3 | 0.869 |  | A36 | 0.924 |  | B11 | 0.851 |  | A36 | 0.894 |  | B11 | 0.885 |  | A36 | 0.940 |  | B3 | 0.871 |  | B11 | 0.948 |
| 12 | A30 | 0.868 |  | A26 | 0.924 |  | A7 | 0.848 |  | A34 | 0.894 |  | A7 | 0.873 |  | A2 | 0.937 |  | B15 | 0.871 |  | A30 | 0.946 |
| 13 | B11 | 0.868 |  | B16 | 0.923 |  | A30 | 0.847 |  | B16 | 0.894 |  | A30 | 0.867 |  | A34 | 0.936 |  | B6 | 0.871 |  | A7 | 0.946 |
| 14 | A7 | 0.868 |  | A2 | 0.923 |  | A17 | 0.847 |  | A2 | 0.893 |  | A17 | 0.860 |  | B16 | 0.936 |  | B1 | 0.871 |  | A17 | 0.945 |
| 15 | A17 | 0.864 |  | A16 | 0.923 |  | B14 | 0.845 |  | A31 | 0.893 |  | B14 | 0.851 |  | A31 | 0.936 |  | B5 | 0.871 |  | B14 | 0.944 |
| 16 | A32 | 0.864 |  | A30 | 0.922 |  | A32 | 0.842 |  | A26 | 0.893 |  | A32 | 0.837 |  | A16 | 0.935 |  | A5 | 0.871 |  | A32 | 0.941 |
| 17 | A26 | 0.862 |  | A11 | 0.922 |  | A2 | 0.841 |  | A11 | 0.892 |  | A2 | 0.833 |  | A26 | 0.933 |  | B8 | 0.871 |  | A24 | 0.940 |
| 18 | A24 | 0.862 |  | A31 | 0.922 |  | A13 | 0.841 |  | A16 | 0.892 |  | A13 | 0.830 |  | A11 | 0.932 |  | B10 | 0.871 |  | A26 | 0.940 |
| 19 | A2 | 0.861 |  | A24 | 0.921 |  | A10 | 0.841 |  | A27 | 0.892 |  | A24 | 0.830 |  | A39 | 0.932 |  | B2 | 0.871 |  | A2 | 0.939 |
| 20 | A34 | 0.861 |  | A27 | 0.921 |  | A24 | 0.841 |  | A30 | 0.891 |  | A10 | 0.829 |  | A27 | 0.932 |  | B9 | 0.871 |  | A10 | 0.939 |
| 21 | A36 | 0.860 |  | B14 | 0.920 |  | A12 | 0.840 |  | A39 | 0.891 |  | A36 | 0.826 |  | A30 | 0.930 |  | B4 | 0.871 |  | A13 | 0.939 |
| 22 | A19 | 0.860 |  | A39 | 0.920 |  | A36 | 0.840 |  | A24 | 0.890 |  | A26 | 0.826 |  | A24 | 0.927 |  | A22 | 0.870 |  | A34 | 0.938 |
| 23 | A13 | 0.859 |  | A15 | 0.918 |  | A26 | 0.840 |  | A13 | 0.887 |  | A12 | 0.825 |  | A13 | 0.924 |  | A15 | 0.869 |  | A36 | 0.938 |
| 24 | A10 | 0.859 |  | A13 | 0.917 |  | B4 | 0.840 |  | A15 | 0.886 |  | B4 | 0.821 |  | A15 | 0.923 |  | A10 | 0.869 |  | A12 | 0.938 |
| 25 | A12 | 0.858 |  | A22 | 0.916 |  | B9 | 0.839 |  | A22 | 0.885 |  | B9 | 0.817 |  | A22 | 0.919 |  | A8 | 0.869 |  | B4 | 0.938 |
| 26 | B4 | 0.857 |  | A14 | 0.916 |  | B2 | 0.839 |  | A14 | 0.884 |  | A34 | 0.816 |  | A14 | 0.919 |  | A14 | 0.869 |  | B9 | 0.937 |
| 27 | A18 | 0.857 |  | A23 | 0.916 |  | B10 | 0.839 |  | A12 | 0.884 |  | B2 | 0.815 |  | A12 | 0.919 |  | A4 | 0.868 |  | B2 | 0.937 |
| 28 | B9 | 0.857 |  | B13 | 0.915 |  | B8 | 0.839 |  | A23 | 0.884 |  | B10 | 0.815 |  | A3 | 0.918 |  | A9 | 0.866 |  | B10 | 0.937 |
| 29 | B2 | 0.857 |  | A12 | 0.915 |  | A34 | 0.838 |  | A3 | 0.884 |  | B8 | 0.815 |  | A23 | 0.917 |  | A11 | 0.863 |  | B8 | 0.937 |
| 30 | B10 | 0.857 |  | B7 | 0.915 |  | B5 | 0.838 |  | A4 | 0.883 |  | B5 | 0.811 |  | A4 | 0.917 |  | A40 | 0.863 |  | B5 | 0.936 |
| 31 | B8 | 0.857 |  | A4 | 0.915 |  | B6 | 0.838 |  | B13 | 0.883 |  | B6 | 0.810 |  | B13 | 0.916 |  | A12 | 0.862 |  | B6 | 0.936 |
| 32 | B5 | 0.856 |  | B3 | 0.914 |  | B1 | 0.837 |  | B7 | 0.883 |  | B1 | 0.809 |  | B7 | 0.916 |  | B16 | 0.861 |  | A19 | 0.936 |
| 33 | B6 | 0.856 |  | A5 | 0.914 |  | B15 | 0.837 |  | A8 | 0.882 |  | B15 | 0.809 |  | A8 | 0.914 |  | A16 | 0.861 |  | B1 | 0.936 |
| 34 | B1 | 0.856 |  | A8 | 0.914 |  | B12 | 0.837 |  | B3 | 0.882 |  | B12 | 0.806 |  | B3 | 0.914 |  | A27 | 0.859 |  | B15 | 0.936 |
| 35 | B15 | 0.856 |  | B12 | 0.914 |  | A8 | 0.837 |  | A5 | 0.882 |  | A8 | 0.805 |  | A5 | 0.914 |  | A13 | 0.859 |  | B12 | 0.935 |
| 36 | B12 | 0.855 |  | A3 | 0.913 |  | A5 | 0.836 |  | B12 | 0.882 |  | A5 | 0.803 |  | B12 | 0.913 |  | A20 | 0.858 |  | A8 | 0.935 |
| 37 | A8 | 0.855 |  | B15 | 0.913 |  | B3 | 0.836 |  | A40 | 0.882 |  | B3 | 0.802 |  | B15 | 0.913 |  | A19 | 0.857 |  | A5 | 0.935 |
| 38 | A5 | 0.855 |  | B1 | 0.913 |  | A4 | 0.836 |  | B15 | 0.882 |  | A19 | 0.801 |  | B1 | 0.913 |  | A3 | 0.857 |  | B3 | 0.935 |
| 39 | B3 | 0.855 |  | B6 | 0.913 |  | A19 | 0.835 |  | B1 | 0.882 |  | A4 | 0.800 |  | B6 | 0.913 |  | A18 | 0.856 |  | A4 | 0.934 |
| 40 | A20 | 0.854 |  | B5 | 0.913 |  | B7 | 0.835 |  | B6 | 0.882 |  | B7 | 0.793 |  | B5 | 0.913 |  | A1 | 0.854 |  | A18 | 0.933 |
| 41 | A4 | 0.854 |  | B8 | 0.913 |  | B13 | 0.834 |  | B5 | 0.881 |  | B13 | 0.793 |  | A40 | 0.912 |  | A36 | 0.854 |  | B7 | 0.933 |
| 42 | A9 | 0.854 |  | B10 | 0.913 |  | A23 | 0.834 |  | B8 | 0.881 |  | A23 | 0.789 |  | B8 | 0.912 |  | A2 | 0.854 |  | B13 | 0.933 |
| 43 | A23 | 0.854 |  | B2 | 0.913 |  | A18 | 0.833 |  | B10 | 0.881 |  | A18 | 0.788 |  | B10 | 0.912 |  | B17 | 0.854 |  | A23 | 0.933 |
| 44 | B7 | 0.854 |  | B9 | 0.913 |  | A14 | 0.833 |  | B2 | 0.881 |  | A14 | 0.785 |  | B2 | 0.912 |  | B14 | 0.853 |  | A14 | 0.932 |
| 45 | B13 | 0.853 |  | B4 | 0.912 |  | A22 | 0.832 |  | B9 | 0.881 |  | A22 | 0.781 |  | B9 | 0.912 |  | A32 | 0.852 |  | A22 | 0.931 |
| 46 | A16 | 0.852 |  | A17 | 0.912 |  | A16 | 0.831 |  | B4 | 0.881 |  | A16 | 0.779 |  | B4 | 0.911 |  | A6 | 0.852 |  | A16 | 0.931 |
| 47 | A14 | 0.852 |  | A10 | 0.912 |  | A15 | 0.830 |  | A17 | 0.881 |  | A15 | 0.769 |  | A10 | 0.911 |  | A39 | 0.851 |  | A20 | 0.930 |
| 48 | A22 | 0.852 |  | A40 | 0.911 |  | A20 | 0.830 |  | A10 | 0.880 |  | A20 | 0.769 |  | A17 | 0.910 |  | A7 | 0.851 |  | A9 | 0.930 |
| 49 | A15 | 0.850 |  | B11 | 0.911 |  | A9 | 0.828 |  | B11 | 0.880 |  | A9 | 0.759 |  | B11 | 0.908 |  | A31 | 0.850 |  | A15 | 0.929 |

## **Table S20** OPLS-DA analysis results of characteristic peaks and each pharmacodynamic index of HQQFD

| **No.** | **Lung injury score** | |  | **TNF-α** | |  | **IL-6** | |  | **IL-1β** | |  | **W/D ratio of Lung** | |  | **Protein in BALF** | |  | **Neutrophils in BALF** | |  | **Overall rating** | |
| --- | --- | --- | --- | --- | --- | --- | --- | --- | --- | --- | --- | --- | --- | --- | --- | --- | --- | --- | --- | --- | --- | --- | --- |
|  | **Peaks** | **VIP** |  | **Peaks** | **VIP** |  | **Peaks** | **VIP** |  | **Peaks** | **VIP** |  | **Peaks** | **VIP** |  | **Peaks** | **VIP** |  | **Peaks** | **VIP** |  | **Peaks** | **VIP** |
| 1 | A20 | 1.604 |  | A7 | 1.352 |  | B16 | 1.321 |  | A18 | 1.587 |  | B14 | 1.367 |  | A40 | 1.946 |  | A7 | 1.500 |  | A11 | 1.413 |
| 2 | A18 | 1.569 |  | A24 | 1.347 |  | B14 | 1.304 |  | A20 | 1.552 |  | B16 | 1.361 |  | A9 | 1.868 |  | A24 | 1.493 |  | A16 | 1.412 |
| 3 | A40 | 1.560 |  | A32 | 1.342 |  | A11 | 1.280 |  | A40 | 1.446 |  | A11 | 1.358 |  | A20 | 1.813 |  | A30 | 1.483 |  | B14 | 1.409 |
| 4 | A11 | 1.516 |  | A17 | 1.305 |  | A27 | 1.275 |  | A32 | 1.408 |  | A16 | 1.354 |  | A19 | 1.685 |  | A36 | 1.465 |  | B16 | 1.392 |
| 5 | A16 | 1.477 |  | A30 | 1.276 |  | A15 | 1.263 |  | B11 | 1.402 |  | A27 | 1.270 |  | A18 | 1.607 |  | A2 | 1.462 |  | A27 | 1.298 |
| 6 | A27 | 1.453 |  | A36 | 1.274 |  | A16 | 1.256 |  | A11 | 1.372 |  | A15 | 1.209 |  | B11 | 1.431 |  | A26 | 1.444 |  | A13 | 1.207 |
| 7 | B14 | 1.442 |  | A2 | 1.249 |  | A14 | 1.245 |  | A16 | 1.332 |  | A4 | 1.200 |  | A27 | 1.368 |  | A34 | 1.376 |  | A36 | 1.188 |
| 8 | B16 | 1.404 |  | A26 | 1.230 |  | A22 | 1.227 |  | A17 | 1.327 |  | A14 | 1.198 |  | A3 | 1.216 |  | A31 | 1.364 |  | A12 | 1.186 |
| 9 | A19 | 1.323 |  | B11 | 1.202 |  | A4 | 1.226 |  | A27 | 1.316 |  | A12 | 1.183 |  | A17 | 1.166 |  | A39 | 1.307 |  | A15 | 1.178 |
| 10 | B11 | 1.311 |  | A31 | 1.201 |  | A1 | 1.172 |  | A36 | 1.308 |  | A13 | 1.167 |  | A32 | 1.158 |  | A32 | 1.271 |  | A4 | 1.167 |
| 11 | A36 | 1.291 |  | A34 | 1.139 |  | A8 | 1.168 |  | A19 | 1.292 |  | A22 | 1.166 |  | A10 | 1.020 |  | A17 | 1.203 |  | A14 | 1.161 |
| 12 | A32 | 1.230 |  | A18 | 1.130 |  | B13 | 1.148 |  | B14 | 1.280 |  | A8 | 1.115 |  | A11 | 1.018 |  | A13 | 1.179 |  | A2 | 1.159 |
| 13 | A2 | 1.207 |  | A23 | 1.117 |  | B7 | 1.146 |  | B16 | 1.240 |  | A1 | 1.100 |  | B16 | 0.994 |  | A16 | 1.088 |  | A22 | 1.123 |
| 14 | A30 | 1.154 |  | A39 | 1.092 |  | B17 | 1.145 |  | A2 | 1.212 |  | A36 | 1.091 |  | B4 | 0.965 |  | B11 | 1.086 |  | A30 | 1.106 |
| 15 | A17 | 1.143 |  | A6 | 1.058 |  | A5 | 1.125 |  | A30 | 1.191 |  | B13 | 1.070 |  | B14 | 0.946 |  | A18 | 1.062 |  | A8 | 1.066 |
| 16 | A9 | 1.092 |  | B4 | 0.995 |  | A12 | 1.123 |  | A7 | 1.073 |  | B7 | 1.068 |  | B9 | 0.942 |  | A11 | 1.000 |  | A1 | 1.047 |
| 17 | A13 | 1.036 |  | B9 | 0.992 |  | B3 | 1.119 |  | A24 | 1.049 |  | A2 | 1.067 |  | B2 | 0.927 |  | A3 | 0.993 |  | B13 | 1.014 |
| 18 | A15 | 0.969 |  | B2 | 0.989 |  | B12 | 1.104 |  | A15 | 0.892 |  | B17 | 1.062 |  | B10 | 0.927 |  | A23 | 0.984 |  | B7 | 1.012 |
| 19 | A12 | 0.950 |  | B10 | 0.989 |  | B15 | 1.096 |  | A9 | 0.872 |  | A5 | 1.051 |  | B8 | 0.925 |  | A12 | 0.960 |  | B17 | 1.006 |
| 20 | A14 | 0.895 |  | B8 | 0.988 |  | B1 | 1.095 |  | A23 | 0.858 |  | B3 | 1.042 |  | A15 | 0.908 |  | B14 | 0.947 |  | A5 | 0.994 |
| 21 | A4 | 0.895 |  | B5 | 0.982 |  | B6 | 1.093 |  | A26 | 0.856 |  | B12 | 1.026 |  | B5 | 0.898 |  | A6 | 0.911 |  | B3 | 0.984 |
| 22 | A22 | 0.855 |  | B6 | 0.981 |  | B5 | 1.089 |  | A6 | 0.833 |  | B15 | 1.020 |  | B6 | 0.891 |  | B16 | 0.856 |  | A18 | 0.978 |
| 23 | A7 | 0.843 |  | B1 | 0.979 |  | B8 | 1.074 |  | B4 | 0.821 |  | B1 | 1.019 |  | B1 | 0.888 |  | B4 | 0.843 |  | B12 | 0.968 |
| 24 | A10 | 0.805 |  | B15 | 0.979 |  | B10 | 1.073 |  | B9 | 0.812 |  | B6 | 1.017 |  | B15 | 0.887 |  | B9 | 0.840 |  | B15 | 0.962 |
| 25 | A24 | 0.802 |  | B12 | 0.977 |  | B2 | 1.073 |  | A22 | 0.808 |  | B5 | 1.013 |  | B12 | 0.871 |  | B2 | 0.838 |  | B1 | 0.961 |
| 26 | A8 | 0.800 |  | B3 | 0.965 |  | B9 | 1.065 |  | B2 | 0.806 |  | A30 | 1.011 |  | A16 | 0.861 |  | B10 | 0.838 |  | B6 | 0.959 |
| 27 | B4 | 0.771 |  | A5 | 0.954 |  | A10 | 1.054 |  | B10 | 0.806 |  | B8 | 0.999 |  | A5 | 0.854 |  | B8 | 0.837 |  | B5 | 0.955 |
| 28 | A1 | 0.765 |  | B17 | 0.945 |  | B4 | 1.053 |  | B8 | 0.806 |  | B10 | 0.998 |  | B3 | 0.854 |  | B5 | 0.832 |  | A20 | 0.944 |
| 29 | B9 | 0.760 |  | B7 | 0.942 |  | A6 | 1.038 |  | A14 | 0.799 |  | B2 | 0.998 |  | A8 | 0.837 |  | B6 | 0.831 |  | B8 | 0.941 |
| 30 | B2 | 0.754 |  | A19 | 0.941 |  | A13 | 1.038 |  | A13 | 0.798 |  | A10 | 0.992 |  | A6 | 0.833 |  | B1 | 0.829 |  | B10 | 0.940 |
| 31 | B10 | 0.753 |  | B13 | 0.939 |  | A23 | 0.971 |  | B5 | 0.797 |  | B9 | 0.990 |  | B17 | 0.832 |  | B15 | 0.829 |  | B2 | 0.940 |
| 32 | B8 | 0.753 |  | A10 | 0.928 |  | A36 | 0.891 |  | B6 | 0.795 |  | B4 | 0.979 |  | A39 | 0.832 |  | B12 | 0.827 |  | A10 | 0.938 |
| 33 | B5 | 0.742 |  | A16 | 0.926 |  | A2 | 0.861 |  | B1 | 0.794 |  | A6 | 0.937 |  | B7 | 0.831 |  | A20 | 0.823 |  | B9 | 0.932 |
| 34 | B6 | 0.740 |  | A13 | 0.915 |  | A20 | 0.854 |  | B15 | 0.793 |  | A18 | 0.896 |  | B13 | 0.830 |  | B3 | 0.817 |  | B4 | 0.922 |
| 35 | B1 | 0.739 |  | A20 | 0.911 |  | A18 | 0.846 |  | B17 | 0.793 |  | A20 | 0.868 |  | A22 | 0.829 |  | A5 | 0.809 |  | A6 | 0.875 |
| 36 | A5 | 0.739 |  | A1 | 0.908 |  | A40 | 0.839 |  | B12 | 0.792 |  | A23 | 0.867 |  | A1 | 0.824 |  | A19 | 0.801 |  | A40 | 0.874 |
| 37 | B15 | 0.739 |  | A8 | 0.871 |  | A30 | 0.798 |  | B3 | 0.785 |  | A40 | 0.810 |  | A23 | 0.821 |  | A10 | 0.798 |  | A32 | 0.843 |
| 38 | B13 | 0.736 |  | A11 | 0.869 |  | B11 | 0.786 |  | B13 | 0.784 |  | A32 | 0.792 |  | A14 | 0.800 |  | B17 | 0.797 |  | B11 | 0.823 |
| 39 | B7 | 0.735 |  | A22 | 0.836 |  | A17 | 0.775 |  | B7 | 0.784 |  | B11 | 0.785 |  | A4 | 0.766 |  | B7 | 0.796 |  | A7 | 0.812 |
| 40 | B12 | 0.732 |  | B14 | 0.819 |  | A19 | 0.764 |  | A1 | 0.778 |  | A17 | 0.772 |  | A31 | 0.700 |  | B13 | 0.794 |  | A23 | 0.806 |
| 41 | B3 | 0.731 |  | A14 | 0.811 |  | A32 | 0.755 |  | A5 | 0.778 |  | A19 | 0.724 |  | A12 | 0.662 |  | A1 | 0.772 |  | A17 | 0.797 |
| 42 | B17 | 0.724 |  | A4 | 0.796 |  | A7 | 0.513 |  | A10 | 0.771 |  | A7 | 0.721 |  | A34 | 0.626 |  | A8 | 0.767 |  | A24 | 0.782 |
| 43 | A26 | 0.708 |  | A12 | 0.771 |  | A3 | 0.508 |  | A31 | 0.737 |  | A26 | 0.696 |  | A26 | 0.428 |  | A4 | 0.762 |  | A26 | 0.781 |
| 44 | A3 | 0.620 |  | A15 | 0.768 |  | A9 | 0.502 |  | A4 | 0.732 |  | A24 | 0.694 |  | A13 | 0.421 |  | A14 | 0.727 |  | A19 | 0.746 |
| 45 | A6 | 0.605 |  | B16 | 0.760 |  | A24 | 0.490 |  | A8 | 0.727 |  | A34 | 0.624 |  | A36 | 0.409 |  | A22 | 0.723 |  | A34 | 0.701 |
| 46 | A34 | 0.581 |  | A3 | 0.687 |  | A26 | 0.460 |  | A34 | 0.686 |  | A3 | 0.615 |  | A24 | 0.360 |  | A15 | 0.673 |  | A3 | 0.634 |
| 47 | A23 | 0.554 |  | A27 | 0.613 |  | A34 | 0.388 |  | A12 | 0.636 |  | A31 | 0.460 |  | A7 | 0.336 |  | A27 | 0.619 |  | A31 | 0.526 |
| 48 | A31 | 0.396 |  | A40 | 0.611 |  | A31 | 0.280 |  | A39 | 0.581 |  | A39 | 0.442 |  | A2 | 0.239 |  | A40 | 0.511 |  | A39 | 0.511 |
| 49 | A39 | 0.372 |  | A9 | 0.232 |  | A39 | 0.213 |  | A3 | 0.025 |  | A9 | 0.397 |  | A30 | 0.196 |  | A9 | 0.398 |  | A9 | 0.410 |

## **Fig. S1** Optimization of UPLC separation conditions (column brands) of HQQFD. **A** 254 nm **B** 440 nm. (**a**. Shimadzu; **b**. Waters; **c**; Agilent)

## **Fig. S2** Optimization of UPLC separation conditions (types of acids in the aqueous phase) of HQQFD. **A** 254 nm. **B** 440 nm. (a 0.15% Acetic acid-water; b 0.15%Formic acid-water; c 0.15% Phosphoric acid)

## **Fig. S3** Optimization of UPLC separation conditions (organic phase) of HQQFD. **A** 254 nm. **B** 440 nm (a acetonitrile; b acetonitrile: methanol=5:1; c acetonitrile: methanol=4:1; d acetonitrile: methanol=2:1; e acetonitrile: methanol=1:1; f methanol)

## **Fig. S4** Optimization of UPLC separation conditions (acid concentration) of HQQFD. **A** under 254 nm. **B** 440 nm (a 0.10% Formic acid-water acetonitrile; b 0.15% Formic acid-water; c 0.30% Formic acid-water acetonitrile)

## **Fig. S5** Optimization of UPLC separation conditions (flow rate) of HQQFD. (**A** 254 nm. **B** 440 nm (a 0.15 mL/min; b 0.20 mL/min; c 0.25 mL/min)

## **Fig. S6** Optimization of UPLC separation conditions (column temperature) of HQQFD. **A** under 254 nm. **B** 440 nm (a 40℃; b 45℃; c 50℃)

## **Fig. S7** Total ion chromatogram (TIC) of HQQFD. (A TIC of PW2. B TIC of PW3. C TIC of PW4

**Structural identification of representative components**

**Flavonoids**

The identification processes of baicalin (peak 62, C₂₁H₁₈O₁₁) and baicalein (peak 90, C₁₅H₁₀O₅) were taken as examples for illustration. The precursor ion of baicalin was m/z 445.07 [M-H]⁻, and its key fragment ion m/z 269.04 originated from the loss of C₆H₈O₆ (glucuronic acid, GluA). The precursor ion of baicalein was m/z 269.04 [M-H]⁻, and its fragmentation pathway showed that the loss of H₂O to form m/z 251.03 was followed by further loss of CO₂ (generating m/z 207.04) or two CO molecules (generating m/z 223.04). The latter (m/z 223.04) could continue to lose CO to produce m/z 195.04 or lose both CO and CO₂ to form m/z 179.05. In addition, m/z 241.05, generated by the loss of CO, could further eliminate CO₂ to form m/z 197.06, which then lost another CO molecule to produce m/z 169.06, as seen in Fig. S8 and Fig. S9.

Fig. S8 Identification results of baicalin by mass spectrometry. A Extracted ion chromatogram (EIC). B Full-scan mass spectrum MS¹. C Tandem mass spectrum (MS²). D Detailed fragmentations and proposed fragment pathway for baicalin

## **Fig. S9** Identification results of baicalein by mass spectrometry. **A** EIC. **B** MS¹. **C** MS². **D** Detailed fragmentations and proposed fragment pathway for baicalein

**Terpenoids**

Taking geniposide (peak 23, C₁₇H₂₄O₁₀) and crocin I (peak 64, C₄₄H₆₄O₂₄) as examples, their fragmentation processes were illustrated. Their precursor ions were m/z 433.13 [M+HCOO]⁻ and m/z 1021.37 [M+HCOO]⁻, respectively. The key fragment ion of geniposide, m/z 225.07 [M-H-Glu]⁻, could further lose H₂O to form m/z 207.06 [M-H-Glu-H₂O]⁻ or lose C₄H₆O₃ to generate m/z 123.04 [M-H-Glu-C₄H₆O₃]⁻. Additionally, the fragment m/z 207.06 could undergo further fragmentation by losing C₄H₄O₄ to produce m/z 123.04 [M-H-Glu-H₂O-C₄H₄O₄]⁻ or losing C₇H₆O to form m/z 101.02 [M-H-C₆H₁₀O₅-H₂O-C₇H₆O]⁻. For crocin I, the characteristic fragment ion m/z 651.26 [M-2Glu]⁻ continued to lose two glucosyl groups to form m/z 327.26 [M-4Glu]⁻, which could further eliminate CO₂ to generate m/z 283.17 [M-4Glu-CO₂]⁻ or sequentially lose two CO₂ molecules to produce m/z 239.18 [M-4Glu-2CO₂]⁻, as seen in Fig. S10 and Fig. S11.

## **Fig. S10** Identification results of geniposide by mass spectrometry. **A** EIC. **B** MS¹. C MS². D Detailed fragmentations and proposed fragment pathway for geniposide

Fig. S11 Identification results of C-I by mass spectrometry. A EIC. B MS¹. C MS². D Detailed fragmentations and proposed fragment pathway for C-I

## **Fig. S12** PCA analysis of chemical variations of of HQQFD across four groups. A score plot. B loading plot

## **Fig. S13** Permutation test results (200 iterations) of OPLS-DA models for different comparison groups. **A** OPLS-DA permutation test for PW1 vs. PW2 vs. PW3 vs. PW4. **B** OPLS-DA permutation test for PW1 vs. PW2. **C** OPLS-DA permutation test for PW1 vs. PW3. **D** OPLS-DA permutation test for PW2 vs. PW4. E OPLS-DA permutation test for PW3 vs. PW4. **F** OPLS-DA permutation test for PW1 vs. PW4. **G** OPLS-DA permutation test for PW2 vs. PW4

## **The calculation procedure of AHP-EWM**

The analytic hierarchy process-entropy weight method (AHP-EWM) are used to determine the weight of factors. AHP is based on subjective information and EWM is based on the degree of information disorder in the assessment system, AHP is vulnerable to the perceptions of interviewees and EWM is vulnerable to extreme values. To reduce the potential bias caused by AHP, EWM was employed for objectively weighting. The procedure unfolds as follows:

### Step1. AHP analysis

The sum-product method is used to calculate the AHP weights, and the calculation process is shown in Eq. S1.

1. Construction of judgement matrix

Based on recent pharmacological studies on acute lung injury, a judgement matrix was constructed using a two-by-two comparison method to rank the importance of the seven pharmacodynamic indicators (Table S1).

1. Calculation of weights for indicators

Calculate the maximum characteristic root λ_max_ of each judgement matrix and the weight vector W to get the relative importance of each element of this level relative to the quality markers of the previous level. Take judgement matrix A as an example, according to Eq. S2, the columns of judgement matrix A will be normalised, and then according to Eq. S3, the sum of each row of judgement matrix A will be obtained, which is the weight vector W, and then according to Eq. S4, W will be normalised, which is the weight of each evaluation index, according to Eq. S5, λ_max_ will be obtained.

1. Consistency test

Because AHP is subjectively underpinned, the judgment matrixes for consistency need to be tested. In Eq.S6, CI is the consistency test index, λ_max_ is the maximum characteristic root obtained according to Eq. S5, and CR is the consistency test ratio in Eq.S7, where RI is the random consistency index (n=1, RI=0, n=2, RI=0, n=3, RI=0.52, n=4, RI=0.89, n=5, RI=1.12, n=6, RI=1.26, n=7, RI=1.36, n=8, RI=1.41, n=9, RI=1.46, n=10, RI=1.49, n=11, RI=1.52, n=12, RI=1.54, n=13, RI=1.56, n=14, RI=1.58, n=15, RI=1.59, n=16, RI=1.594). A random consistency ratio (CR) below 0.1 indicates satisfactory consistency, otherwise, the matrix must be adjusted until it conforms. The result of AHP analysis was shown in Table S15.

|  | Eq. S1 |
| --- | --- |
| $\bar{a_{ij}}=\frac{a_{ij}}{\sum_{i=1}^{n} a_{ij}}$(i, j=1, 2, …., n) | Eq. S2 |
| $W_{i}=\sum_{j=1}^{n} \bar{a_{ij}}$ (i=1, 2, …., n) | Eq. S3 |
| $w_{i}=\frac{W_{i}}{\sum_{i=1}^{n} W_{i}}$ (i=1, 2, …., n) | Eq. S4 |
| $\lambda_{max}=\frac{1}{n}\sum_{i=1}^{n} \frac{AW}{W_{i}}$ (i=1, 2, …., n) | Eq. S5 |
| $CI=\frac{\lambda_{max}-n}{n-1}$ | Eq. S6 |
| $CR=\frac{CI}{RI}$ | Eq. S7 |

### Step2. EWM analysis

EWM is an important information weighting model that has been widely studied, and its advantage is that the indicator weights can avoid the interference of human factors and enhance the objectivity of the comprehensive evaluation results. For data with *j* indicators and *i* samples, the entropy weight method calculates the weights as follows:

In equation Eq. S8, y*_ij_* is the standardised value of the *j* indicator in the *i* sample, In Eq. S9, p*_ij_* is the probability of the *j* indicator in the *i* experiment after standardization, in Eq. S10, e*_j_* is the entropy value of the *j* indicator, in Eq. S11, d*_j_* is the coefficient of variation of the j indicator, and in Eq. S12, v*_j_* is the weight value of the j indicator. The result of EWM analysis was shown in Table S16.

| $y_{ij}=\frac{x_{ij}-\min x_{ij}}{\max x_{ij}-\min x_{ij}}$ | Eq. S8 |
| --- | --- |
| $P_{ij}=\frac{y_{ij}}{\sum_{i=1}^{m} y_{ij}}$ | Eq. S9 |
| $e_{j}=-\frac{1}{\ln m}\sum_{i=1}^{m} P_{ij}\ln P_{ij}$ | Eq. S10 |
| $d_{j}=1-e_{j}$ | Eq. S11 |
| $vj=\frac{d_{j}}{\sum_{j=1}^{n} d_{j}}$ | Eq. S12 |

### Step3. Calculation of combined weight

In order to avoid the calculation deviation caused by the separate calculation of the two weight calculation methods, this paper used the AHP-EWM coupling evaluation model to synthesize the calculation results of the two weighting methods, and the calculation formula of final weight of factor *j* (T*_j_*) was shown in Eq. S13.

The result of combined weight was shown in Table S17.

| $T_{j}=\frac{w_{j}\times v_{j}}{\sum_{i=1}^{n} (w_{j}\times v_{j})}$ | Eq. S13 |
| --- | --- |

### Step4. Calculation of overall rating

The overall rating of efficacy indicators was shown in Eq. S14. To ensure data normalization, it was necessary to convert various efficacy indicators into a non-dimensional criterion (*Z_i_*), which was conducted by IBM SPSS Statistics 29.0, where *T_i_* is the comprehensive weight of the evaluation index. The result was shown in Table 5.

| $Overall rating=\sum_{i=1}^{n} Z_{i}\times T_{i}$ | Eq. S14 |
| --- | --- |
